# Genetically predicted phosphate and cardiovascular disease: A Mendelian randomization study

**DOI:** 10.3389/fcvm.2022.973338

**Published:** 2022-10-05

**Authors:** Jiniu Huang, Chenyun Zhang, Qinyan Gong, Ying Gao, Xiaojie Xie, Jun Jiang

**Affiliations:** Department of Cardiology, The Second Affiliated Hospital, Zhejiang University School of Medicine, Hangzhou, China

**Keywords:** phosphate, cardiovascular disease, Mendelian randomization study, valvular heart disease, causality

## Abstract

**Background:**

Extensive epidemiological studies have highlighted the correlation between serum phosphate and cardiovascular diseases. The present study aims to determine whether genetically predicted serum phosphate is causally associated with the distinct subtypes of cardiovascular events through the use of Mendelian randomization (MR) analysis.

**Methods:**

Independent and strongly correlated single-nucleotide polymorphisms (SNPs) for serum phosphate were extracted from publicly available genome-wide association studies. Summary statistics of cardiovascular diseases were derived from large-scale consortiums, including HERMES and FinnGen biobank. MR-Egger, weighted median, inverse variance weighted, pleiotropy residual sum and outlier (MR-PRESSO) methods and MR using robust adjusted profile score (MR-RAPS) were employed to analyze causality. The sensitivity analyses comprised heterogeneity, horizontal pleiotropy, and leave-one-out approaches; these were used to ensure the stability of the results.

**Results:**

Our study demonstrated that increased genetically predicted serum phosphate is causally associated with a higher risk of valvular heart disease (VHD) [For VHD including rheumatic fever: odds ratio (OR) = 2.45; 95% confidence interval (CI), 1.52–3.94; *p* = 0.0002; for non-rheumatic VHD: OR = 6.58; 95% CI, 2.50–17.32; *p* = 0.0001]. However, no causal association was detected between serum phosphate and other common cardiovascular diseases (including coronary heart disease, heart failure, atrial fibrillation, and essential hypertension).

**Conclusions:**

The results indicate strong causality between serum phosphate and valvular heart disease. Serum phosphate-lowering therapy within the physiological range may represent a novel therapeutic method for valvular heart disease.

## Introduction

Cardiovascular disease (CVD), a predominant cause of death worldwide, largely contributes to the global burden of disease (1–3). Despite advancements in diagnosis and treatment, further exploration of causative factors is required (4, 5).

Phosphate plays an essential role in various physiological and pathological processes involved in energy metabolism, cellular structure, and signal transduction (6–8). Extensive studies have discussed the epidemiological link between serum phosphate and cardiovascular events, including atherosclerosis (9–11), ischemic heart disease (12, 13), hypertension (14), heart failure (15), and valvular heart disease (VHD) (16–18). However, according to the IMPROVE-CKD study and the LANDMARK randomized clinical trial, treatment with lanthanum carbonate, an intestinal phosphate binder, does not result in a significant difference in the occurrence of composite cardiovascular events in chronic kidney disease with normophosphatemia or hyperphosphatemia (19, 20). The paradoxical role of phosphate in CVDs is a pressing issue that must be addressed, and additional evidence is needed to demonstrate that phosphate clearly precedes CVDs. Furthermore, traditional epidemiology is subject to reverse causation and residual biases. It is unable to ascertain whether serum phosphate is an important preventable cause of CVDs.

Numerous studies have attempted to determine the causality between exposures and outcomes through the utilization of Mendelian randomization (MR) analysis (21, 22). With the natural and random distribution of genetic variants, MR is known to be less vulnerable to confounding and reverse causation (23). Using the Mendelian randomization approach, we intend to explore the potential causal relationship between phosphate and five CVDs: coronary heart disease (CHD), heart failure (HF), atrial fibrillation (AF), essential hypertension (EH), and valvular heart disease (VHD).

## Materials and methods

### Study design

**Figure 1:**
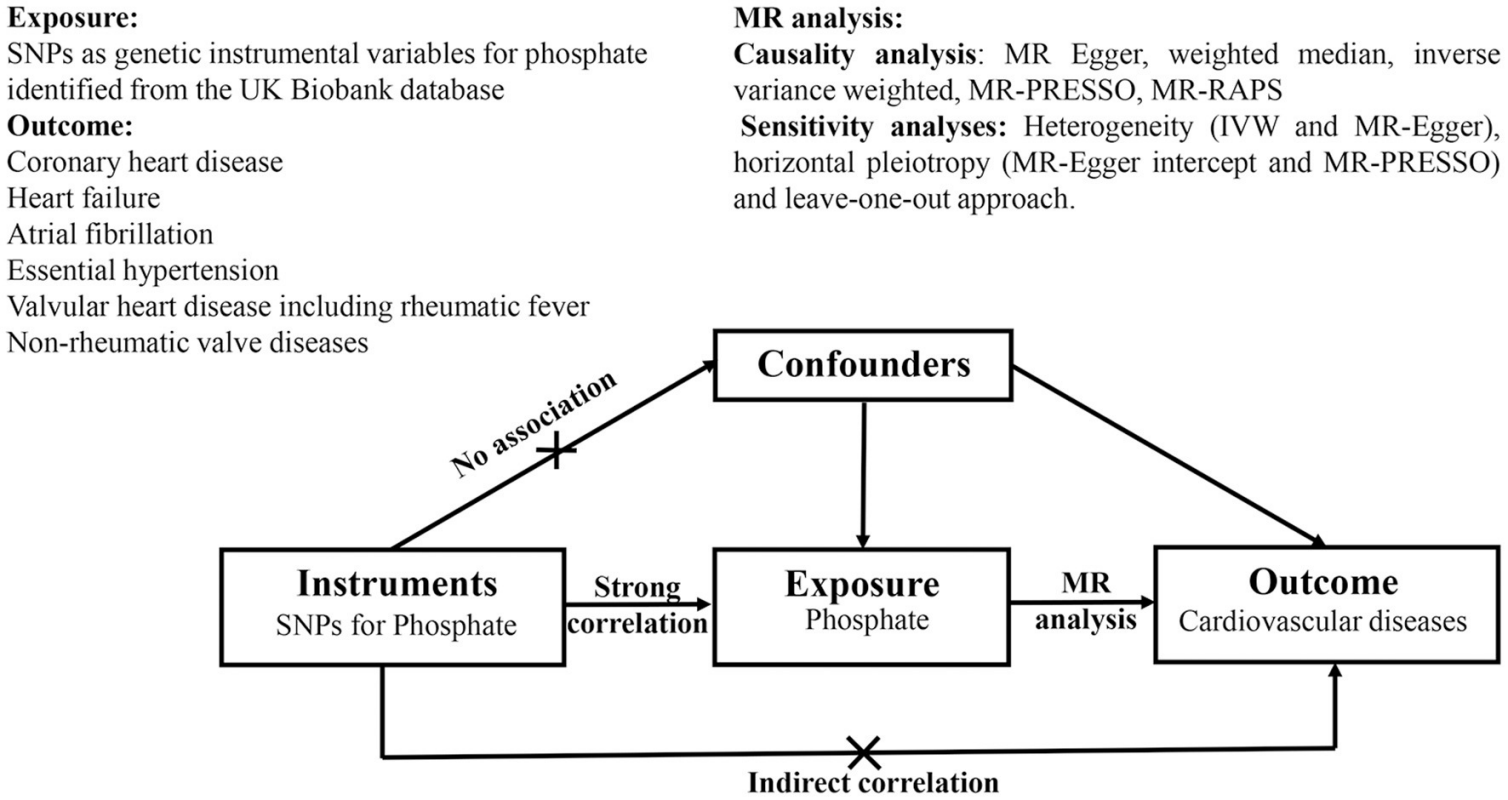
Schematic diagram of Mendelian randomization analyses. SNPs as genetic instruments are used to estimate the causal relationship between phosphate and cardiovascular disease. The genetic variables are not associated with potential confounders. Furthermore, there is no existence of a direct correlation between genetic instruments and outcomes. SNPs, single-nucleotide polymorphisms; MR-PRESSO, MR pleiotropy residual sum and outlier; MR-RAPS, MR using robust adjusted profile score; IVW, inverse variance weighted.

The data used in the two-sample MR analysis are publicly available from the Genome-Wide Association Study (GWAS) Catalog (https://www.ebi.ac.uk/gwas). Ethical approval for the studies and the informed consent of all participants were obtained. An overview of the study design is shown in Figure 1.

### Selection of genetic instrumental variables

We extracted the single-nucleotide polymorphisms (SNPs) from the GWAS data according to two criteria for strong correlation and independence as follows: genome-wide level of statistical significance (5 × 10^−8^) and linkage disequilibrium (LD) with r^2^ < 0.001 and clump window > 10,000 kb. This study collected 159 SNPs as genetic instrumental variables for serum phosphate from the UK Biobank database at (https://gwas.mrcieu.ac.uk/datasets/ukb-d-30810_raw/), which included 431,448 participants of European ancestry. Furthermore, all SNPs were cross-referenced with the PhenoScanner database (http://www.phenoscanner.medschl.cam.ac.uk/) to identify associations with confounders and outcomes (24).

### Outcome data sources

The CVDs-associated SNPs were derived from HERMES, the FinnGen biobank, and other large-scale consortiums. The detailed characterization of each CVDs is shown in Table 1.

**Table 1:** Information on outcome data sources.

**Cardiovascular diseases**	**Consortium**	**Population**	**Cases**	**Controls**
Coronary heart disease	FinnGen biobank	European	21,012	197,780
Heart failure	HERMES	European	47,309	930,014
Atrial fibrillation	AFGen, HUNT, MGI, deCODE, DiscovEHR and UK Biobank	European	60,620	970,216
Essential hypertension	FinnGen biobank	European	42,857	162,837
Valvular heart disease including rheumatic fever	FinnGen biobank	European	38,209	156,711
Non-rheumatic valve diseases	FinnGen biobank	European	10,235	156,711

HERMES, Heart Failure Molecular Epidemiology for Therapeutic Targets; HUNT, the Nord-Trøndelag Health Study; MGI, the Michigan Genomics Initiative.

### Statistical analysis

We performed two-sample MR, which harmonized the SNPs of phosphate and the common CVDs in independent datasets and removed all palindromic SNPs from the analysis. F-statistics was used to assess the strength of genetic variants (25). We estimated the causal effects using five methods: MR Egger, weighted median, inverse variance weighted (IVW), pleiotropy residual sum and outlier (MR-PRESSO) (26), and MR using robust adjusted profile score (MR-RAPS) (27). IVW was regarded as the principal approach (28). Results were presented as odds ratios (ORs) and 95% confidence intervals (CIs) on phosphate risk for common CVDs. The sensitivity analyses comprised three approaches: heterogeneity (IVW and MR-Egger), horizontal pleiotropy (MR-Egger intercept and MR-PRESSO), and leave-one-out. Heterogeneity was measured through Cochran's Q test (29), and outliers were detected *via* MR-PRESSO analysis (26). The intercept in the MR-Egger regression showed evidence for pleiotropic bias and was visualized using funnel plots (30). The leave-one-out SNP analysis was applied to examine the sensitivity of each genetic variant, which was generally used in MR (31, 32). The MR analyses were conducted in R version 4.1.3 (http://www.r-project.org) using the TwoSampleMR package (33).

## Results

### Genetic instrumental variables for phosphate

Initially, 159 SNPs were identified from the GWAS catalog at the genome-wide significance level (*p* < 5 × 10^−8^) and linkage disequilibrium (LD) with r^2^ < 0.001 and clump window >10,000 kb, as shown in Supplementary Table 1. Based on PhenoScanner, several genetic instrumental variables were removed for their associations with confounders of CVDs (including body mass index, blood pressure, smoking, and lipid levels) and direct connections to outcomes (Supplementary Table 2). After harmonizing the SNPs of phosphate and the common CVDs in independent datasets and removing all palindromic SNPs, the final datasets were obtained (Supplementary Table 3). The F-statistics for phosphate were higher than the threshold of 10, which indicates no evidence of weak instrument bias (Supplementary Table 3).

### Causal association of phosphate with CVDs

**Figure 2:**
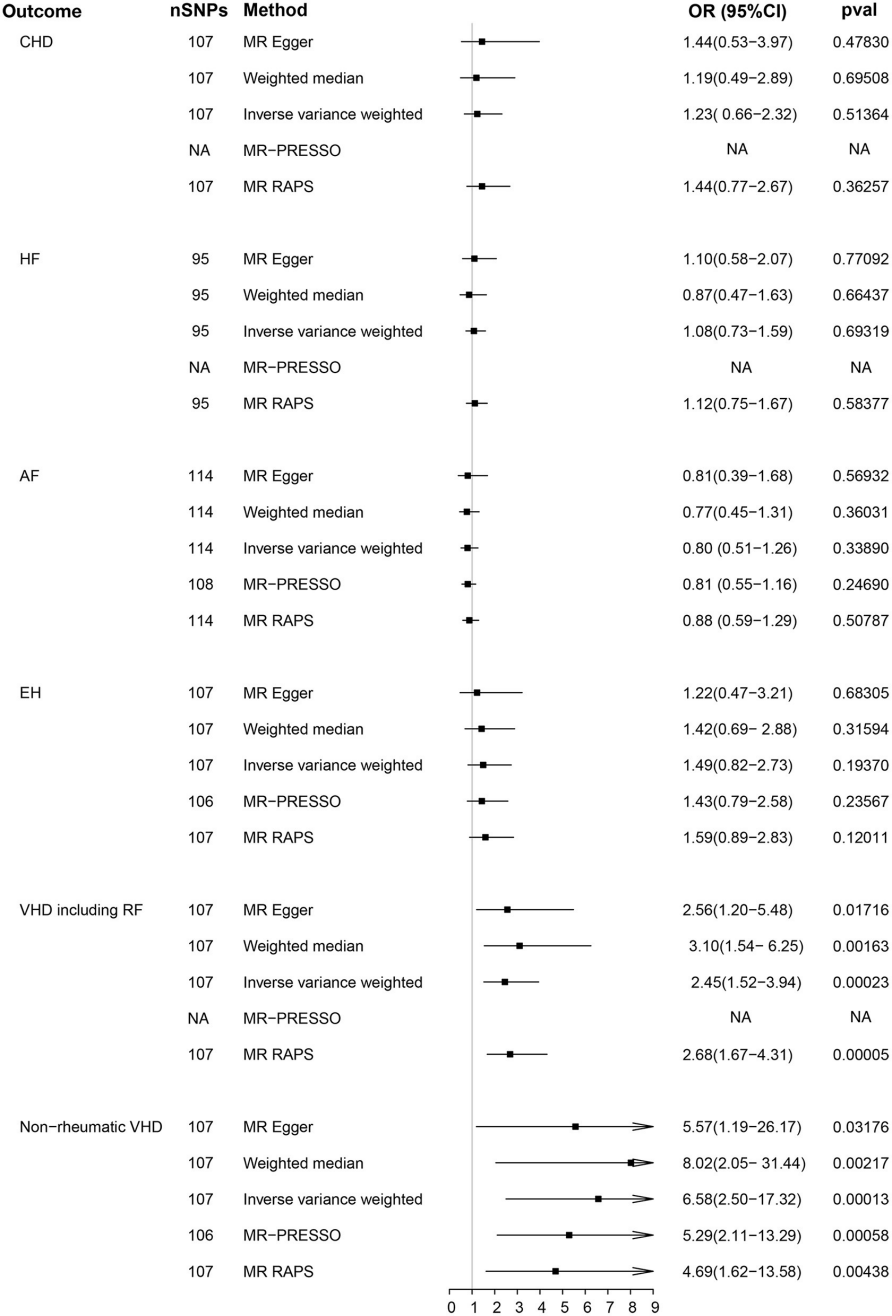
Causality analysis between genetically predicted phosphate and common cardiovascular diseases. *P*-value, OR and 95% CI of five methods [MR Egger, weighted median, inverse variance weighted (IVW), MR-PRESSO, and MR RAPS] are shown in the results. CHD, coronary heart disease; HF, heart failure; AF, atrial fibrillation; EH, essential hypertension; VDH, valvular heart disease; RF, rheumatic fever; MR-PRESSO, MR pleiotropy residual sum and outlier; MR-RAPS, MR using robust adjusted profile score; OR, odds ratio; CI, confidence interval; NA, not available.

The main results of the causal analysis are presented in Figure 2. The IVW method showed that genetically predicted serum phosphate levels were positively associated with VHD [For VHD including rheumatic fever: odds ratio (OR) = 2.45; 95% confidence interval (CI), 1.52–3.94; *p* = 0.0002; for non-rheumatic VHD: OR = 6.58; 95% CI, 2.50–17.32; *p* = 0.0001]. However, no significant difference was detected in other common CVDs (including CHD, HF, AF, and EH). We utilized a scatter diagram and forest plot to visualize the relationship between each genetic variant and CVDs (Supplementary Figures 1, 2). Cochran's Q statistical and MR-PRESSO analyses revealed the existence of heterogeneity and outliers in our study (Supplementary Tables 4, 5). The analyses using two additional methods (MR-PRESSO and MR-RAPS) highlighted a significant association between serum phosphate and VHD, which is directionally consistent with previous methods (Figure 2). No evidence of horizontal pleiotropy was noted in the MR-Egger regression intercept analysis; the results were visualized through a funnel plot (Supplementary Table 4 and Supplementary Figure 3). Based on the results of the leave-one-out analysis, there was no single genetic variant that altered the causality (Supplementary Figure 4).

## Discussion

Numerous epidemiological studies have highlighted the correlation between serum phosphate and CVDs. However, few in-depth investigations about the causality of serum phosphate on different subtypes of cardiovascular events have been conducted. Furthermore, the majority of randomized controlled trials have targeted phosphate-related cardiovascular endpoints in chronic kidney diseases rather than independent cardiovascular events (19, 20, 34). Our study demonstrates a causal relationship between genetically predicted serum phosphate and valvular heart disease. The sensitivity analyses (heterogeneity, horizontal pleiotropy, and leave-one-out approaches) proved the stability of the results.

VHD mainly manifests as valve stenosis or incomplete closure, which results in poor quality of life. The epidemiology of VHD presents substantial regional differences: degenerative diseases predominate in high-income countries, while rheumatic heart diseases predominate in low-and middle-income countries (35, 36). Current Mendelian randomization studies of VHD are mainly centered on lipid (37, 38) and blood pressure (39). Our study provides convincing evidence of the causality between genetically predicted serum phosphate and valvular heart disease, which is consistent with prior epidemiological research (18, 40). However, there is no evidence that suggests a causal association of serum phosphate with other common CVDs (including CHD, HF, AF, and EH) in the present MR studies. This discrepancy may suggest the existence of correlation rather than causality between phosphate and these CVDs, which warrants further research to elucidate the underlying relationship.

Mechanistically, increased phosphate promotes hydroxyapatite deposition in the valves and the osteogenic differentiation of vascular smooth muscle and valvular interstitial cells, which accelerates the process of valve calcification (41–43). Furthermore, an extensive number of studies have revealed that phosphate is correlated with inflammation, which provides suggestive evidence for the causality between serum phosphate and VHD (44–46). Taken together, serum phosphate may play a critical role in the pathogenesis of VHD.

Our study has several strengths. Based on the random distribution of genetic variations in the population, our study minimizes reverse causation and residual biases. Similar assessment results across various approaches for causality ensure the credibility of causality. Furthermore, we performed sensitivity analyses through the combined use of diversified approaches. To avoid potential bias from population stratification, our sample was restricted to individuals of European ancestries. The confirmed causality between serum phosphate and VHD suggests a novel therapeutic method for VHD.

This study has several limitations. First, we had no access to comprehensive information regarding the participants (including age, sex, and other influencing factors), thereby causing inevitable heterogeneity. Second, based on the summary-level data, our study was unable to exclude the presence of non-linear relationships. Finally, the causality between serum phosphate and the specific subgroup of VHD remains to be explored due to the absence of classifications in GWAS databases at present.

## Conclusions

Our results strongly indicate a causal relationship between serum phosphate and valvular heart disease using the MR method. Targeting the serum phosphate homeostasis as a potential therapeutic approach may provide profound implications for valvular heart disease.

## Data availability statement

The datasets presented in this study can be found in online repositories. The names of the repository/repositories and accession number(s) can be found in the article/Supplementary materials.

## Ethics statement

The studies involving human participants were reviewed and approved by Local Ethics Committees of consortia in the respective studies. The patients/participants provided their written informed consent to participate in this study. Written informed consent was obtained from the individual(s) for the publication of any potentially identifiable images or data included in this article.

## Author contributions

JH, XX, and JJ designed the study and wrote the manuscript. JH, CZ, QG, YG, XX, and JJ contributed to the data acquisition and revision of the manuscript. All authors approved the final manuscript.

## Funding

This work was supported by the National Natural Science Foundation of China (Grant No. 82170332).

## Conflict of interest

The authors declare that the research was conducted in the absence of any commercial or financial relationships that could be construed as a potential conflict of interest.

## Publisher's note

All claims expressed in this article are solely those of the authors and do not necessarily represent those of their affiliated organizations, or those of the publisher, the editors and the reviewers. Any product that may be evaluated in this article, or claim that may be made by its manufacturer, is not guaranteed or endorsed by the publisher.

## Abbreviations

CVD: cardiovascular disease
CHD: coronary heart disease
HF: heart failure
AF: atrial fibrillation
EH: essential hypertension
VHD: valvular heart disease
RF: rheumatic fever
MR: Mendelian randomization
SNP: single-nucleotide polymorphism
GWAS: genome-wide association study
IVW: inverse variance weighted
LD: linkage disequilibrium
OR: odds ratio
CI: confidence interval
MR-PRESSO: MR pleiotropy residual sum and outlier
MR-RAPS: MR using robust adjusted profile score.
