# Supplementary material for: Genetically predicted phosphate and cardiovascular disease: A Mendelian randomization study
Source: Front Cardiovasc Med. 2022 Oct 5;9:973338. doi: 10.3389/fcvm.2022.973338 (PMC9579538; doi:10.3389/fcvm.2022.973338)
Supplement: Supplementary file 1 [file Data_Sheet_1.docx]

**Table S1: Characteristics of independent and strongly correlated instrumental variables for phosphate**

| **SNP** | **TRAIT** | **Chromosome:position** | **EA** | **OA** | **EAF** | **Beta** | **SE** | **pval.exposure** |
| --- | --- | --- | --- | --- | --- | --- | --- | --- |
| rs113487566 | Phosphate | 1:51599038 | T | C | 0.0254 | -0.0102 | 0.0013 | 5.87E-16 |
| rs12044944 | Phosphate | 1:240581653 | T | C | 0.1925 | 0.0029 | 0.0005 | 4.29E-09 |
| rs11801716 | Phosphate | 1:42143899 | T | C | 0.0462 | -0.0054 | 0.0009 | 9.47E-09 |
| rs4072537 | Phosphate | 1:1065296 | C | T | 0.6811 | -0.0023 | 0.0004 | 3.95E-08 |
| rs71641333 | Phosphate | 1:78743005 | A | T | 0.0745 | -0.0045 | 0.0008 | 2.73E-09 |
| rs72660359 | Phosphate | 1:21989655 | T | G | 0.0373 | 0.0148 | 0.0010 | 1.30E-45 |
| rs7529925 | Phosphate | 1:199007208 | T | C | 0.7383 | 0.0031 | 0.0004 | 7.01E-12 |
| rs79472736 | Phosphate | 1:22108543 | A | G | 0.0354 | -0.0070 | 0.0011 | 3.45E-11 |
| rs12409158 | Phosphate | 1:68277744 | C | G | 0.0319 | -0.0064 | 0.0011 | 1.70E-08 |
| rs12132412 | Phosphate | 1:21820042 | G | A | 0.3884 | 0.0174 | 0.0004 | 1.00E-200 |
| rs7555660 | Phosphate | 1:21731734 | T | C | 0.0853 | 0.0090 | 0.0007 | 4.91E-38 |
| rs12727188 | Phosphate | 1:159486328 | G | T | 0.0894 | -0.0054 | 0.0007 | 6.82E-15 |
| rs12059422 | Phosphate | 1:159550748 | C | T | 0.0230 | 0.0146 | 0.0013 | 3.32E-29 |
| rs838717 | Phosphate | 2:234296444 | A | G | 0.5656 | 0.0049 | 0.0004 | 3.04E-35 |
| rs6751217 | Phosphate | 2:191360094 | T | C | 0.6316 | 0.0029 | 0.0004 | 6.03E-13 |
| rs55923451 | Phosphate | 2:230111334 | A | G | 0.1791 | -0.0034 | 0.0005 | 5.39E-11 |
| rs77149268 | Phosphate | 2:158113579 | T | C | 0.1051 | 0.0066 | 0.0006 | 5.82E-25 |
| rs72974112 | Phosphate | 2:234889515 | T | C | 0.2373 | -0.0029 | 0.0005 | 4.78E-10 |
| rs2303917 | Phosphate | 2:10092133 | A | G | 0.1077 | -0.0037 | 0.0006 | 6.18E-09 |
| rs878919 | Phosphate | 2:97437990 | C | T | 0.3970 | 0.0032 | 0.0004 | 3.10E-15 |
| rs11684211 | Phosphate | 2:85481524 | A | G | 0.3533 | 0.0026 | 0.0004 | 3.81E-10 |
| rs896788 | Phosphate | 2:7149155 | T | C | 0.1552 | 0.0042 | 0.0005 | 6.52E-15 |
| rs371380082 | Phosphate | 2:234937560 | CA | C | 0.2462 | 0.0027 | 0.0005 | 1.07E-08 |
| rs4855880 | Phosphate | 3:49725685 | A | G | 0.5164 | -0.0022 | 0.0004 | 4.18E-08 |
| rs56328339 | Phosphate | 3:188400557 | T | C | 0.1628 | 0.0052 | 0.0005 | 1.05E-22 |
| rs6780593 | Phosphate | 3:121927927 | A | G | 0.1528 | -0.0045 | 0.0005 | 8.55E-17 |
| rs17036160 | Phosphate | 3:12329783 | T | C | 0.1180 | -0.0040 | 0.0006 | 5.67E-11 |
| rs7625643 | Phosphate | 3:141150026 | G | A | 0.4482 | 0.0026 | 0.0004 | 7.39E-11 |
| rs11705850 | Phosphate | 3:114317538 | C | T | 0.0787 | -0.0044 | 0.0007 | 9.86E-10 |
| rs73186030 | Phosphate | 3:122013465 | T | C | 0.1276 | -0.0116 | 0.0006 | 5.39E-87 |
| rs1483842 | Phosphate | 3:25407695 | C | T | 0.4808 | -0.0028 | 0.0004 | 1.42E-12 |
| rs4690098 | Phosphate | 4:3447156 | T | C | 0.2349 | -0.0026 | 0.0005 | 2.08E-08 |
| rs7672820 | Phosphate | 4:88715259 | C | G | 0.1863 | 0.0043 | 0.0005 | 6.26E-18 |
| rs10013062 | Phosphate | 4:103930220 | G | A | 0.3524 | 0.0023 | 0.0004 | 3.46E-08 |
| rs9799552 | Phosphate | 4:88632117 | A | C | 0.2073 | 0.0036 | 0.0005 | 1.49E-13 |
| rs62289280 | Phosphate | 4:7812818 | C | T | 0.3053 | -0.0029 | 0.0004 | 1.09E-11 |
| rs6841258 | Phosphate | 4:40565426 | T | C | 0.1663 | 0.0031 | 0.0005 | 5.29E-09 |
| rs62383568 | Phosphate | 5:141713195 | C | T | 0.3497 | -0.0024 | 0.0004 | 3.35E-09 |
| rs7726961 | Phosphate | 5:158504140 | T | C | 0.4261 | -0.0023 | 0.0004 | 6.85E-09 |
| rs706300 | Phosphate | 5:14984221 | G | A | 0.8575 | -0.0054 | 0.0006 | 2.96E-22 |
| rs10900829 | Phosphate | 5:133848917 | G | A | 0.5817 | 0.0029 | 0.0004 | 1.92E-13 |
| rs10051765 | Phosphate | 5:176799992 | C | T | 0.3324 | -0.0082 | 0.0004 | 3.03E-87 |
| rs12518871 | Phosphate | 5:36634332 | T | C | 0.4095 | -0.0025 | 0.0004 | 2.57E-10 |
| rs1432679 | Phosphate | 5:158244083 | T | C | 0.5529 | 0.0024 | 0.0004 | 1.62E-09 |
| rs56795888 | Phosphate | 5:88339400 | GT | G | 0.4612 | -0.0022 | 0.0004 | 1.78E-08 |
| rs7705189 | Phosphate | 5:131623358 | G | A | 0.4671 | -0.0025 | 0.0004 | 4.64E-10 |
| rs6911602 | Phosphate | 6:132088762 | A | G | 0.1318 | 0.0082 | 0.0006 | 5.07E-46 |
| rs912981 | Phosphate | 6:18914211 | C | A | 0.5810 | -0.0026 | 0.0004 | 3.22E-11 |
| rs805288 | Phosphate | 6:31678028 | T | C | 0.2491 | 0.0025 | 0.0005 | 4.98E-08 |
| rs60447213 | Phosphate | 6:33715159 | T | G | 0.0699 | -0.0299 | 0.0008 | 1.00E-200 |
| rs145330264 | Phosphate | 6:33689778 | C | A | 0.0054 | -0.0321 | 0.0028 | 5.75E-30 |
| rs4706554 | Phosphate | 6:74481076 | A | G | 0.4763 | 0.0029 | 0.0004 | 2.22E-13 |
| rs4709746 | Phosphate | 6:164133001 | T | C | 0.1341 | 0.0035 | 0.0006 | 1.97E-09 |
| rs2982572 | Phosphate | 6:152010561 | T | C | 0.4208 | -0.0041 | 0.0004 | 1.83E-25 |
| rs74551305 | Phosphate | 6:45283663 | C | T | 0.0560 | 0.0074 | 0.0009 | 1.23E-16 |
| rs375640583 | Phosphate | 6:163860688 | CTT | C | 0.2487 | -0.0028 | 0.0005 | 1.70E-09 |
| rs453639 | Phosphate | 6:132049657 | A | C | 0.6405 | -0.0107 | 0.0004 | 2.10E-150 |
| rs3813498 | Phosphate | 6:108944165 | T | C | 0.8128 | 0.0051 | 0.0005 | 3.42E-24 |
| rs4869745 | Phosphate | 6:151908076 | T | C | 0.2871 | 0.0031 | 0.0004 | 6.73E-13 |
| rs35552719 | Phosphate | 6:136087428 | T | G | 0.2855 | 0.0049 | 0.0004 | 2.08E-28 |
| rs56078331 | Phosphate | 6:44647753 | T | C | 0.0544 | 0.0084 | 0.0009 | 2.07E-22 |
| rs9689096 | Phosphate | 6:34188892 | C | A | 0.0639 | -0.0070 | 0.0008 | 4.31E-18 |
| rs1889208 | Phosphate | 6:132239500 | A | G | 0.3359 | 0.0033 | 0.0004 | 3.76E-15 |
| rs9388490 | Phosphate | 6:126704795 | T | C | 0.4373 | 0.0027 | 0.0004 | 4.59E-12 |
| rs17288460 | Phosphate | 6:45729130 | A | G | 0.1803 | -0.0033 | 0.0005 | 7.84E-11 |
| rs13208790 | Phosphate | 6:121784588 | C | T | 0.6506 | 0.0024 | 0.0004 | 9.66E-09 |
| rs370250672 | Phosphate | 6:163861903 | GT | G | 0.2606 | -0.0025 | 0.0005 | 1.92E-08 |
| rs2177470 | Phosphate | 7:38152697 | A | T | 0.6819 | -0.0027 | 0.0004 | 2.60E-10 |
| rs801013 | Phosphate | 7:74237612 | T | G | 0.1572 | -0.0033 | 0.0005 | 2.35E-09 |
| rs4410790 | Phosphate | 7:17284577 | C | T | 0.6342 | 0.0027 | 0.0004 | 2.41E-11 |
| rs2460421 | Phosphate | 7:65491123 | T | A | 0.5019 | -0.0022 | 0.0004 | 2.85E-08 |
| rs10279171 | Phosphate | 7:101663639 | T | A | 0.7941 | 0.0029 | 0.0005 | 2.04E-09 |
| rs3757677 | Phosphate | 7:28474745 | G | A | 0.3721 | 0.0030 | 0.0004 | 6.84E-14 |
| rs10282449 | Phosphate | 7:20055003 | A | T | 0.1663 | 0.0037 | 0.0005 | 2.15E-12 |
| rs13232861 | Phosphate | 7:97929081 | G | A | 0.8125 | 0.0031 | 0.0005 | 3.76E-10 |
| rs144214505 | Phosphate | 7:6102499 | T | C | 0.0416 | -0.0057 | 0.0010 | 7.05E-09 |
| rs2941483 | Phosphate | 8:76478616 | G | A | 0.5710 | 0.0029 | 0.0004 | 1.27E-13 |
| rs7017252 | Phosphate | 8:129950844 | T | C | 0.3830 | 0.0022 | 0.0004 | 3.53E-08 |
| rs6983239 | Phosphate | 8:72507296 | T | G | 0.2186 | 0.0039 | 0.0005 | 3.52E-16 |
| rs13273161 | Phosphate | 8:8205234 | T | A | 0.3177 | 0.0030 | 0.0004 | 9.94E-13 |
| rs7860558 | Phosphate | 9:129300073 | C | T | 0.7013 | -0.0029 | 0.0004 | 3.13E-11 |
| rs72716022 | Phosphate | 9:71179615 | T | C | 0.2021 | -0.0033 | 0.0005 | 1.09E-10 |
| rs296847 | Phosphate | 9:4793798 | T | G | 0.6643 | 0.0024 | 0.0004 | 8.87E-09 |
| rs28542318 | Phosphate | 9:140130606 | T | A | 0.8344 | 0.0032 | 0.0005 | 1.75E-09 |
| rs7033278 | Phosphate | 9:71417662 | T | C | 0.2930 | 0.0035 | 0.0004 | 5.96E-16 |
| rs11144134 | Phosphate | 9:77499796 | C | T | 0.0845 | 0.0043 | 0.0007 | 7.16E-10 |
| rs138893847 | Phosphate | 10:9329356 | GTCAT | G | 0.0945 | -0.0066 | 0.0007 | 2.13E-22 |
| rs4509682 | Phosphate | 10:50313621 | A | G | 0.3919 | -0.0031 | 0.0004 | 5.48E-15 |
| rs11191643 | Phosphate | 10:105057561 | G | C | 0.4380 | 0.0028 | 0.0004 | 1.76E-12 |
| rs77282753 | Phosphate | 10:101812499 | C | T | 0.0480 | -0.0069 | 0.0009 | 4.06E-14 |
| rs6650130 | Phosphate | 10:22280664 | T | A | 0.7132 | 0.0032 | 0.0004 | 2.78E-13 |
| rs2420055 | Phosphate | 10:116080355 | C | T | 0.8426 | -0.0035 | 0.0005 | 4.21E-11 |
| rs12770024 | Phosphate | 10:130922153 | G | C | 0.1936 | 0.0037 | 0.0005 | 4.92E-14 |
| rs485411 | Phosphate | 10:8093185 | C | T | 0.7484 | 0.0032 | 0.0005 | 6.17E-13 |
| rs10896012 | Phosphate | 11:65278461 | C | T | 0.2179 | -0.0041 | 0.0005 | 6.47E-18 |
| rs200358011 | Phosphate | 11:13497473 | TA | T | 0.1512 | -0.0039 | 0.0005 | 7.91E-13 |
| rs2959652 | Phosphate | 11:64520942 | T | G | 0.9080 | -0.0037 | 0.0007 | 3.13E-08 |
| rs273587 | Phosphate | 11:30883876 | T | A | 0.6778 | -0.0030 | 0.0004 | 7.53E-13 |
| rs11021221 | Phosphate | 11:95308854 | A | T | 0.1712 | -0.0029 | 0.0005 | 2.18E-08 |
| rs73632745 | Phosphate | 11:126229617 | T | C | 0.0736 | 0.0041 | 0.0007 | 3.37E-08 |
| rs4938642 | Phosphate | 11:119099906 | C | G | 0.0740 | 0.0041 | 0.0007 | 4.74E-08 |
| rs61870274 | Phosphate | 11:11431043 | A | C | 0.0781 | 0.0059 | 0.0007 | 7.92E-16 |
| rs61909254 | Phosphate | 12:4486891 | C | T | 0.8598 | 0.0101 | 0.0006 | 8.49E-72 |
| rs17884869 | Phosphate | 12:123519112 | A | G | 0.0250 | 0.0079 | 0.0013 | 2.58E-10 |
| rs145978363 | Phosphate | 12:4818287 | G | A | 0.0301 | -0.0072 | 0.0012 | 1.89E-09 |
| rs2970818 | Phosphate | 12:4606168 | A | T | 0.1038 | 0.0206 | 0.0006 | 1.00E-200 |
| rs10743976 | Phosphate | 12:12166475 | C | A | 0.8512 | 0.0067 | 0.0005 | 7.00E-34 |
| rs596940 | Phosphate | 12:123560731 | C | T | 0.0716 | 0.0052 | 0.0008 | 1.16E-11 |
| rs138278885 | Phosphate | 12:94311258 | CA | C | 0.1007 | -0.0057 | 0.0007 | 3.68E-18 |
| rs1468908 | Phosphate | 12:107298317 | G | A | 0.4920 | 0.0050 | 0.0004 | 6.28E-37 |
| rs653178 | Phosphate | 12:112007756 | T | C | 0.5174 | -0.0040 | 0.0004 | 3.60E-24 |
| rs4759844 | Phosphate | 12:131578055 | G | A | 0.3509 | 0.0038 | 0.0004 | 1.22E-20 |
| rs35026779 | Phosphate | 12:113563105 | T | C | 0.0997 | -0.0042 | 0.0007 | 9.54E-11 |
| rs7324259 | Phosphate | 13:33509030 | T | G | 0.1259 | 0.0037 | 0.0006 | 4.73E-10 |
| rs1570603 | Phosphate | 13:51385422 | A | G | 0.5720 | -0.0022 | 0.0004 | 3.69E-08 |
| rs9574586 | Phosphate | 13:80749431 | C | T | 0.3311 | -0.0023 | 0.0004 | 2.35E-08 |
| rs4982711 | Phosphate | 14:23398990 | G | T | 0.5710 | 0.0023 | 0.0004 | 1.05E-08 |
| rs1286070 | Phosphate | 14:91449394 | C | T | 0.1721 | -0.0041 | 0.0005 | 2.43E-15 |
| rs71413981 | Phosphate | 14:23774916 | A | G | 0.1637 | -0.0031 | 0.0005 | 6.93E-09 |
| rs34933034 | Phosphate | 15:75079474 | A | G | 0.1529 | -0.0057 | 0.0005 | 7.19E-26 |
| rs12902894 | Phosphate | 15:51243964 | C | A | 0.4580 | 0.0044 | 0.0004 | 8.14E-29 |
| rs339969 | Phosphate | 15:60883281 | A | C | 0.6148 | 0.0027 | 0.0004 | 2.35E-11 |
| rs4077450 | Phosphate | 16:79931595 | T | G | 0.8280 | -0.0042 | 0.0005 | 1.15E-15 |
| rs11646780 | Phosphate | 16:80022802 | A | T | 0.5841 | -0.0026 | 0.0004 | 1.09E-10 |
| rs76819459 | Phosphate | 16:79718917 | G | A | 0.3270 | -0.0051 | 0.0004 | 1.15E-34 |
| rs3213473 | Phosphate | 16:16256767 | A | T | 0.0343 | 0.0132 | 0.0011 | 6.75E-31 |
| rs41278174 | Phosphate | 16:16259596 | A | G | 0.0273 | 0.0170 | 0.0012 | 1.31E-45 |
| rs2230742 | Phosphate | 16:4016676 | G | A | 0.8457 | -0.0041 | 0.0005 | 6.74E-14 |
| rs9745989 | Phosphate | 16:49888931 | C | T | 0.6281 | 0.0025 | 0.0004 | 9.31E-10 |
| rs2120222 | Phosphate | 17:58996208 | C | A | 0.1485 | -0.0092 | 0.0005 | 9.73E-63 |
| rs55938136 | Phosphate | 17:43798360 | G | A | 0.2257 | -0.0044 | 0.0005 | 6.77E-21 |
| rs71368113 | Phosphate | 17:27278573 | G | C | 0.0709 | -0.0045 | 0.0008 | 4.44E-08 |
| rs8072297 | Phosphate | 17:37478048 | T | A | 0.2310 | -0.0057 | 0.0005 | 3.29E-33 |
| rs4795607 | Phosphate | 17:29726603 | T | C | 0.6140 | 0.0024 | 0.0004 | 1.58E-09 |
| rs5435 | Phosphate | 17:7187123 | C | T | 0.6121 | -0.0022 | 0.0004 | 4.08E-08 |
| rs11078597 | Phosphate | 17:1618363 | C | T | 0.1864 | 0.0055 | 0.0005 | 4.83E-28 |
| rs12941512 | Phosphate | 17:76932762 | T | C | 0.4422 | -0.0032 | 0.0004 | 2.07E-16 |
| rs2955382 | Phosphate | 17:17947710 | T | C | 0.6121 | -0.0031 | 0.0004 | 4.82E-15 |
| rs8075449 | Phosphate | 17:66632565 | T | C | 0.2590 | -0.0028 | 0.0004 | 5.03E-10 |
| rs2909212 | Phosphate | 17:66458006 | G | A | 0.2966 | 0.0024 | 0.0004 | 2.31E-08 |
| rs872386 | Phosphate | 17:66702640 | A | G | 0.5310 | -0.0039 | 0.0004 | 2.60E-23 |
| rs76527351 | Phosphate | 17:44212527 | G | A | 0.2257 | -0.0044 | 0.0005 | 7.59E-21 |
| rs111723017 | Phosphate | 17:57787431 | C | G | 0.0875 | 0.0053 | 0.0007 | 1.89E-14 |
| rs58468752 | Phosphate | 17:45661289 | TA | T | 0.4864 | -0.0026 | 0.0004 | 6.98E-11 |
| rs3897629 | Phosphate | 18:44340086 | A | T | 0.4963 | -0.0024 | 0.0004 | 4.76E-10 |
| rs139796809 | Phosphate | 19:3069499 | T | C | 0.5570 | -0.0028 | 0.0004 | 1.53E-11 |
| rs12710029 | Phosphate | 19:35664583 | C | T | 0.7762 | -0.0029 | 0.0005 | 7.06E-10 |
| rs10401230 | Phosphate | 19:53405497 | G | A | 0.4937 | -0.0022 | 0.0004 | 9.47E-09 |
| rs8105161 | Phosphate | 19:41839631 | C | T | 0.1553 | -0.0036 | 0.0006 | 1.14E-10 |
| rs78030362 | Phosphate | 19:18575193 | G | A | 0.0736 | 0.0063 | 0.0008 | 6.50E-17 |
| rs10415758 | Phosphate | 19:14172921 | T | A | 0.4225 | -0.0026 | 0.0004 | 5.15E-11 |
| rs12985272 | Phosphate | 19:17543681 | A | G | 0.0494 | 0.0064 | 0.0009 | 4.69E-12 |
| rs308032 | Phosphate | 19:3102748 | T | G | 0.2368 | -0.0029 | 0.0005 | 1.03E-09 |
| rs209961 | Phosphate | 20:52715154 | C | T | 0.7219 | 0.0037 | 0.0004 | 1.38E-17 |
| rs17265513 | Phosphate | 20:39832628 | C | T | 0.1991 | 0.0043 | 0.0005 | 6.74E-19 |
| rs3091842 | Phosphate | 20:39344272 | A | G | 0.0441 | -0.0086 | 0.0010 | 1.49E-18 |
| rs35666081 | Phosphate | 20:45590298 | A | G | 0.4002 | 0.0022 | 0.0004 | 4.52E-08 |
| rs6136492 | Phosphate | 20:1923972 | T | C | 0.2259 | -0.0027 | 0.0005 | 5.88E-09 |
| rs4820324 | Phosphate | 22:38599857 | C | G | 0.5810 | 0.0030 | 0.0004 | 3.78E-14 |

**Abbreviations:** **SNP**: single-nucleotide polymorphism; **EA**, effect allele; **OA**, other allele; **EAF**, effect allele frequency; **SE**, standard error.

**Table S2: Associations of used SNPs for phosphate in the PhenoScanner**

| **rsID** | **Chromosome:position** | **EA** | **OA** | **Trait** | **PMID** | **Beta** | **pval** | **N** | **Unit** |
| --- | --- | --- | --- | --- | --- | --- | --- | --- | --- |
| rs71641333 | chr1:78743005 | A | T | Body mass index | UKBB | 0.0230 | 1.01E-06 | 336107 | IVNT |
| rs11705850 | chr3:114317538 | C | T | Body mass index | UKBB | -0.0224 | 5.33E-07 | 336107 | IVNT |
| rs73186030 | chr3:122013465 | C | T | Diastolic blood pressure | UKBB | 0.0220 | 4.17E-09 | 317756 | IVNT |
| rs4690098 | chr4:3447156 | C | T | Triglycerides | 24097068 | 0.0207 | 1.32E-06 | 172007 | IVNT |
|  | | | | Total cholesterol | 24097068 | 0.0215 | 1.41E-06 | 181445 | IVNT |
| rs10013062 | chr4:103930220 | A | G | hypertension | UKBB | 0.0057 | 5.05E-07 | 336683 | risk diff |
| rs10900829 | chr5:133848917 | A | G | Body mass index | UKBB | 0.0112 | 4.55E-06 | 336107 | IVNT |
| rs1432679 | chr5:158244083 | C | T | Systolic blood pressure | UKBB | -0.0172 | 3.92E-12 | 317754 | IVNT |
| rs7705189 | chr5:131623358 | A | G | hypertension | 28443625 | -0.0211 | 3.99E-07 | 152141 | IVNT |
| rs4709746 | chr6:164133001 | C | T | Body mass index males | 28892062 | -0.0327 | 5.29E-06 | 90992 | IVNT |
| rs9388490 | chr6:126704795 | C | T | Coronary artery disease | 29212778 | 0.0341 | 3.37E-09 | 547261 | log OR |
| rs13208790 | chr6:121784588 | C | T | Diastolic blood pressure | UKBB | -0.0121 | 3.48E-06 | 317756 | IVNT |
| rs4410790 | chr7:17284577 | C | T | Body mass index | 29273807 | 0.0120 | 3.70E-07 | 449889 | IVNT |
| rs2941483 | chr8:76478616 | A | G | Body mass index | UKBB | -0.0129 | 9.66E-08 | 336107 | IVNT |
| rs11191643 | chr10:105057561 | C | G | Systolic blood pressure | UKBB | 0.0153 | 6.35E-10 | 317754 | IVNT |
| rs6650130 | chr10:22280664 | A | T | Body mass index | UKBB | 0.0199 | 7.86E-14 | 336107 | IVNT |
| rs485411 | chr10:8093185 | C | T | Body mass index | 26426971 | 0.0280 | 5.30E-06 | 80961 | IVNT |
| rs10896012 | chr11:65278461 | C | T | Body mass index | UKBB | 0.0174 | 2.49E-09 | 336107 | IVNT |
| rs11021221 | chr11:95308854 | A | T | Diastolic blood pressure | UKBB | -0.0217 | 3.48E-11 | 317756 | IVNT |
| rs73632745 | chr11:126229617 | C | T | Low density lipoprotein | 24097068 | -0.0615 | 3.21E-10 | 83090 | IVNT |
|  | | | | High density lipoprotein | 24097068 | 0.0455 | 1.98E-06 | 92731 | IVNT |
| rs653178 | chr12:112007756 | T | C | Coronary artery disease | 29212778 | -0.0546 | 1.13E-23 | 547261 | log OR |
|  | | | | Hypertension | 21347282 | NA | 3.00E-18 | 8090 | - |
|  |  |  |  | Total cholesterol | 20686565 | 0.0325 | 3.18E-10 | 100184 | Z-score |
| rs34933034 | chr15:75079474 | A | G | Systolic blood pressure | UKBB | 0.0172 | 5.56E-07 | 317754 | IVNT |
|  | | | | Myocardial infarction | 26343387 | 0.0685 | 9.87E-06 | 167181 | log OR |
| rs2230742 | chr16:4016676 | G | A | Body mass index | UKBB | 0.0184 | 3.01E-08 | 336107 | IVNT |
| rs2120222 | chr17:58996208 | C | A | Coronary artery disease | 29212778 | 0.0455 | 1.38E-08 | 547261 | log OR |
| rs55938136 | chr17:43798360 | A | G | Alcohol intake frequency | UKBB | -0.0267 | 3.00E-10 | 336965 | - |
| rs8072297 | chr17:37478048 | A | T | Rheumatoid arthritis | 24390342 | 0.0770 | 3.60E-07 | 80799 | log OR |
| rs11078597 | chr17:1618363 | C | T | Body mass index | 28892062 | -0.0205 | 3.53E-06 | 173430 | IVNT |
| rs2955382 | chr17:17947710 | C | T | Coronary artery disease | 29212778 | 0.0301 | 3.05E-07 | 547261 | log OR |
|  | |  |  | Body mass index | 28892062 | -0.0326 | 2.68E-06 | 173430 | IVNT |
| rs8105161 | chr19:41839631 | C | T | Coronary artery disease | 29212778 | -0.0501 | 2.92E-10 | 547261 | log OR |
| rs78030362 | chr19:18575193 | A | G | Coronary artery disease | 29212778 | -0.0836 | 3.01E-13 | 547261 | log OR |
| rs17265513 | chr20:39832628 | C | T | Diastolic blood pressure | UKBB | -0.0141 | 4.66E-06 | 317756 | IVNT |

**Abbreviations:** **EA**, effect allele; **OA**, other allele.

**Table S3-1: Characteristics of instrumental variables for phosphate and CHD in the MR analysis**

| **SNP** | **EA** | **OA** | **Exposure** | | | | **CHD** | | | |
| --- | --- | --- | --- | --- | --- | --- | --- | --- | --- | --- |
|  |  |  | **EAF** | **Beta** | **SE** | **pval** | **EAF** | **Beta** | **SE** | **pval** |
| rs10051765 | C | T | 0.3324 | -0.0082 | 0.0004 | 3.03E-87 | 0.4009 | 0.0088 | 0.0132 | 0.5030 |
| rs10279171 | T | A | 0.7941 | 0.0029 | 0.0005 | 2.04E-09 | 0.8680 | -0.0149 | 0.0191 | 0.4359 |
| rs10282449 | A | T | 0.1663 | 0.0037 | 0.0005 | 2.15E-12 | 0.1303 | 0.0054 | 0.0194 | 0.7798 |
| rs10401230 | G | A | 0.4937 | -0.0022 | 0.0004 | 9.47E-09 | 0.4656 | -0.0168 | 0.0130 | 0.1973 |
| rs10743976 | C | A | 0.8512 | 0.0067 | 0.0005 | 7.00E-34 | 0.8817 | -0.0304 | 0.0201 | 0.1313 |
| rs11144134 | C | T | 0.0845 | 0.0043 | 0.0007 | 7.16E-10 | 0.0635 | -0.0129 | 0.0269 | 0.6321 |
| rs111723017 | C | G | 0.0875 | 0.0053 | 0.0007 | 1.89E-14 | 0.1204 | 0.0168 | 0.0198 | 0.3955 |
| rs113487566 | T | C | 0.0254 | -0.0102 | 0.0013 | 5.87E-16 | 0.0591 | 0.0129 | 0.0275 | 0.6387 |
| rs11684211 | A | G | 0.3533 | 0.0026 | 0.0004 | 3.81E-10 | 0.2693 | -0.0119 | 0.0146 | 0.4164 |
| rs11801716 | T | C | 0.0462 | -0.0054 | 0.0009 | 9.47E-09 | 0.0851 | -0.0229 | 0.0233 | 0.3248 |
| rs12044944 | T | C | 0.1925 | 0.0029 | 0.0005 | 4.29E-09 | 0.1394 | 0.0101 | 0.0188 | 0.5906 |
| rs12059422 | C | T | 0.0230 | 0.0146 | 0.0013 | 3.32E-29 | 0.0337 | 0.0382 | 0.0359 | 0.2874 |
| rs12132412 | G | A | 0.3884 | 0.0174 | 0.0004 | 1.00E-200 | 0.4041 | 0.0025 | 0.0132 | 0.8522 |
| rs12409158 | C | G | 0.0319 | -0.0064 | 0.0011 | 1.70E-08 | 0.0146 | 0.0144 | 0.0546 | 0.7921 |
| rs12518871 | T | C | 0.4095 | -0.0025 | 0.0004 | 2.57E-10 | 0.4467 | -0.0119 | 0.0130 | 0.3607 |
| rs12710029 | C | T | 0.7762 | -0.0029 | 0.0005 | 7.06E-10 | 0.7284 | -0.0149 | 0.0147 | 0.3106 |
| rs12727188 | G | T | 0.0894 | -0.0054 | 0.0007 | 6.82E-15 | 0.1204 | 0.0072 | 0.0198 | 0.7154 |
| rs12770024 | G | C | 0.1936 | 0.0037 | 0.0005 | 4.92E-14 | 0.1712 | 0.0233 | 0.0172 | 0.1747 |
| rs1286070 | C | T | 0.1721 | -0.0041 | 0.0005 | 2.43E-15 | 0.1539 | 0.0066 | 0.0180 | 0.7154 |
| rs12941512 | T | C | 0.4422 | -0.0032 | 0.0004 | 2.07E-16 | 0.5194 | 0.0305 | 0.0131 | 0.0196 |
| rs12985272 | A | G | 0.0494 | 0.0064 | 0.0009 | 4.69E-12 | 0.0364 | -0.0374 | 0.0353 | 0.2890 |
| rs13232861 | G | A | 0.8125 | 0.0031 | 0.0005 | 3.76E-10 | 0.8447 | 0.0226 | 0.0179 | 0.2073 |
| rs13273161 | T | A | 0.3177 | 0.0030 | 0.0004 | 9.94E-13 | 0.3596 | -0.0078 | 0.0147 | 0.5970 |
| rs138278885 | CA | C | 0.1007 | -0.0057 | 0.0007 | 3.68E-18 | 0.0883 | -0.0501 | 0.0228 | 0.0281 |
| rs139796809 | T | C | 0.5570 | -0.0028 | 0.0004 | 1.53E-11 | 0.5512 | 0.0162 | 0.0133 | 0.2240 |
| rs144214505 | T | C | 0.0416 | -0.0057 | 0.0010 | 7.05E-09 | 0.0365 | -0.0416 | 0.0345 | 0.2277 |
| rs145330264 | C | A | 0.0054 | -0.0321 | 0.0028 | 5.75E-30 | 0.0009 | -0.3085 | 0.2157 | 0.1526 |
| rs145978363 | G | A | 0.0301 | -0.0072 | 0.0012 | 1.89E-09 | 0.0178 | -0.0188 | 0.0497 | 0.7059 |
| rs1468908 | G | A | 0.4920 | 0.0050 | 0.0004 | 6.28E-37 | 0.5382 | 0.0054 | 0.0130 | 0.6781 |
| rs1483842 | C | T | 0.4808 | -0.0028 | 0.0004 | 1.42E-12 | 0.4856 | 0.0076 | 0.0129 | 0.5561 |
| rs1570603 | A | G | 0.5720 | -0.0022 | 0.0004 | 3.69E-08 | 0.5822 | -0.0060 | 0.0131 | 0.6470 |
| rs17036160 | T | C | 0.1180 | -0.0040 | 0.0006 | 5.67E-11 | 0.1713 | 0.0153 | 0.0172 | 0.3748 |
| rs17288460 | A | G | 0.1803 | -0.0033 | 0.0005 | 7.84E-11 | 0.1763 | -0.0193 | 0.0169 | 0.2540 |
| rs17884869 | A | G | 0.0250 | 0.0079 | 0.0013 | 2.58E-10 | 0.0360 | -0.0020 | 0.0348 | 0.9550 |
| rs1889208 | A | G | 0.3359 | 0.0033 | 0.0004 | 3.76E-15 | 0.3857 | -0.0259 | 0.0133 | 0.0509 |
| rs209961 | C | T | 0.7219 | 0.0037 | 0.0004 | 1.38E-17 | 0.7850 | -0.0372 | 0.0158 | 0.0183 |
| rs2177470 | A | T | 0.6819 | -0.0027 | 0.0004 | 2.60E-10 | 0.7257 | -0.0173 | 0.0146 | 0.2361 |
| rs2420055 | C | T | 0.8426 | -0.0035 | 0.0005 | 4.21E-11 | 0.8673 | -0.0221 | 0.0190 | 0.2450 |
| rs273587 | T | A | 0.6778 | -0.0030 | 0.0004 | 7.53E-13 | 0.6246 | 0.0095 | 0.0133 | 0.4765 |
| rs28542318 | T | A | 0.8344 | 0.0032 | 0.0005 | 1.75E-09 | 0.7487 | -0.0259 | 0.0157 | 0.0988 |
| rs2909212 | G | A | 0.2966 | 0.0024 | 0.0004 | 2.31E-08 | 0.2808 | -0.0009 | 0.0144 | 0.9506 |
| rs2959652 | T | G | 0.9080 | -0.0037 | 0.0007 | 3.13E-08 | 0.8945 | 0.0127 | 0.0209 | 0.5436 |
| rs296847 | T | G | 0.6643 | 0.0024 | 0.0004 | 8.87E-09 | 0.6397 | 0.0073 | 0.0135 | 0.5871 |
| rs2970818 | A | T | 0.1038 | 0.0206 | 0.0006 | 1.00E-200 | 0.0622 | 0.0433 | 0.0267 | 0.1043 |
| rs2982572 | T | C | 0.4208 | -0.0041 | 0.0004 | 1.83E-25 | 0.4626 | 0.0061 | 0.0129 | 0.6387 |
| rs308032 | T | G | 0.2368 | -0.0029 | 0.0005 | 1.03E-09 | 0.1668 | -0.0170 | 0.0174 | 0.3293 |
| rs3091842 | A | G | 0.0441 | -0.0086 | 0.0010 | 1.49E-18 | 0.0887 | 0.0205 | 0.0229 | 0.3712 |
| rs339969 | A | C | 0.6148 | 0.0027 | 0.0004 | 2.35E-11 | 0.7197 | -0.0322 | 0.0144 | 0.0252 |
| rs35026779 | T | C | 0.0997 | -0.0042 | 0.0007 | 9.54E-11 | 0.0981 | 0.0054 | 0.0219 | 0.8041 |
| rs35666081 | A | G | 0.4002 | 0.0022 | 0.0004 | 4.52E-08 | 0.4776 | 0.0080 | 0.0130 | 0.5378 |
| rs3813498 | T | C | 0.8128 | 0.0051 | 0.0005 | 3.42E-24 | 0.7763 | 0.0003 | 0.0155 | 0.9854 |
| rs4072537 | C | T | 0.6811 | -0.0023 | 0.0004 | 3.95E-08 | 0.6385 | -0.0045 | 0.0136 | 0.7382 |
| rs4077450 | T | G | 0.8280 | -0.0042 | 0.0005 | 1.15E-15 | 0.7979 | 0.0006 | 0.0161 | 0.9710 |
| rs41278174 | A | G | 0.0273 | 0.0170 | 0.0012 | 1.31E-45 | 0.0332 | 0.0170 | 0.0358 | 0.6341 |
| rs4509682 | A | G | 0.3919 | -0.0031 | 0.0004 | 5.48E-15 | 0.3744 | -0.0051 | 0.0133 | 0.7031 |
| rs453639 | A | C | 0.6405 | -0.0107 | 0.0004 | 2.10E-150 | 0.6662 | 0.0084 | 0.0139 | 0.5448 |
| rs4706554 | A | G | 0.4763 | 0.0029 | 0.0004 | 2.22E-13 | 0.4132 | 0.0151 | 0.0131 | 0.2482 |
| rs4759844 | G | A | 0.3509 | 0.0038 | 0.0004 | 1.22E-20 | 0.2864 | 0.0275 | 0.0143 | 0.0544 |
| rs4795607 | T | C | 0.6140 | 0.0024 | 0.0004 | 1.58E-09 | 0.5965 | 0.0053 | 0.0132 | 0.6880 |
| rs4820324 | C | G | 0.5810 | 0.0030 | 0.0004 | 3.78E-14 | 0.5819 | -0.0080 | 0.0131 | 0.5383 |
| rs4869745 | T | C | 0.2871 | 0.0031 | 0.0004 | 6.73E-13 | 0.2945 | 0.0233 | 0.0142 | 0.1014 |
| rs4938642 | C | G | 0.0740 | 0.0041 | 0.0007 | 4.74E-08 | 0.0273 | 0.0327 | 0.0401 | 0.4139 |
| rs4982711 | G | T | 0.5710 | 0.0023 | 0.0004 | 1.05E-08 | 0.5676 | 0.0362 | 0.0130 | 0.0055 |
| rs5435 | C | T | 0.6121 | -0.0022 | 0.0004 | 4.08E-08 | 0.6211 | 0.0002 | 0.0134 | 0.9902 |
| rs55923451 | A | G | 0.1791 | -0.0034 | 0.0005 | 5.39E-11 | 0.1407 | 0.0039 | 0.0186 | 0.8330 |
| rs56078331 | T | C | 0.0544 | 0.0084 | 0.0009 | 2.07E-22 | 0.0410 | 0.0362 | 0.0330 | 0.2724 |
| rs56328339 | T | C | 0.1628 | 0.0052 | 0.0005 | 1.05E-22 | 0.2086 | 0.0092 | 0.0160 | 0.5650 |
| rs596940 | C | T | 0.0716 | 0.0052 | 0.0008 | 1.16E-11 | 0.0756 | -0.0351 | 0.0244 | 0.1497 |
| rs60447213 | T | G | 0.0699 | -0.0299 | 0.0008 | 1.00E-200 | 0.1086 | -0.0217 | 0.0211 | 0.3055 |
| rs6136492 | T | C | 0.2259 | -0.0027 | 0.0005 | 5.88E-09 | 0.1897 | 0.0161 | 0.0165 | 0.3292 |
| rs61870274 | A | C | 0.0781 | 0.0059 | 0.0007 | 7.92E-16 | 0.0812 | 0.0390 | 0.0236 | 0.0990 |
| rs61909254 | C | T | 0.8598 | 0.0101 | 0.0006 | 8.49E-72 | 0.8418 | 0.0165 | 0.0178 | 0.3540 |
| rs62289280 | C | T | 0.3053 | -0.0029 | 0.0004 | 1.09E-11 | 0.2725 | 0.0214 | 0.0145 | 0.1400 |
| rs62383568 | C | T | 0.3497 | -0.0024 | 0.0004 | 3.35E-09 | 0.3252 | -0.0244 | 0.0138 | 0.0772 |
| rs6751217 | T | C | 0.6316 | 0.0029 | 0.0004 | 6.03E-13 | 0.6197 | 0.0026 | 0.0133 | 0.8472 |
| rs6780593 | A | G | 0.1528 | -0.0045 | 0.0005 | 8.55E-17 | 0.1769 | 0.0281 | 0.0169 | 0.0965 |
| rs6841258 | T | C | 0.1663 | 0.0031 | 0.0005 | 5.29E-09 | 0.1304 | 0.0075 | 0.0193 | 0.6962 |
| rs6911602 | A | G | 0.1318 | 0.0082 | 0.0006 | 5.07E-46 | 0.1113 | -0.0040 | 0.0206 | 0.8473 |
| rs6983239 | T | G | 0.2186 | 0.0039 | 0.0005 | 3.52E-16 | 0.1873 | 0.0043 | 0.0165 | 0.7960 |
| rs7017252 | T | C | 0.3830 | 0.0022 | 0.0004 | 3.53E-08 | 0.2948 | 0.0065 | 0.0142 | 0.6455 |
| rs7033278 | T | C | 0.2930 | 0.0035 | 0.0004 | 5.96E-16 | 0.3156 | -0.0068 | 0.0139 | 0.6271 |
| rs706300 | G | A | 0.8575 | -0.0054 | 0.0006 | 2.96E-22 | 0.8817 | 0.0026 | 0.0202 | 0.8986 |
| rs71368113 | G | C | 0.0709 | -0.0045 | 0.0008 | 4.44E-08 | 0.0578 | -0.0184 | 0.0280 | 0.5114 |
| rs71413981 | A | G | 0.1637 | -0.0031 | 0.0005 | 6.93E-09 | 0.1375 | -0.0387 | 0.0189 | 0.0403 |
| rs72660359 | T | G | 0.0373 | 0.0148 | 0.0010 | 1.30E-45 | 0.0356 | 0.0211 | 0.0353 | 0.5497 |
| rs72716022 | T | C | 0.2021 | -0.0033 | 0.0005 | 1.09E-10 | 0.1881 | -0.0037 | 0.0166 | 0.8246 |
| rs72974112 | T | C | 0.2373 | -0.0029 | 0.0005 | 4.78E-10 | 0.1811 | 0.0139 | 0.0170 | 0.4118 |
| rs7324259 | T | G | 0.1259 | 0.0037 | 0.0006 | 4.73E-10 | 0.1636 | -0.0054 | 0.0178 | 0.7623 |
| rs7529925 | T | C | 0.7383 | 0.0031 | 0.0004 | 7.01E-12 | 0.7223 | -0.0023 | 0.0144 | 0.8733 |
| rs7555660 | T | C | 0.0853 | 0.0090 | 0.0007 | 4.91E-38 | 0.0732 | -0.0041 | 0.0252 | 0.8717 |
| rs7625643 | G | A | 0.4482 | 0.0026 | 0.0004 | 7.39E-11 | 0.4595 | -0.0062 | 0.0130 | 0.6329 |
| rs7672820 | C | G | 0.1863 | 0.0043 | 0.0005 | 6.26E-18 | 0.1878 | 0.0008 | 0.0166 | 0.9602 |
| rs76819459 | G | A | 0.3270 | -0.0051 | 0.0004 | 1.15E-34 | 0.3089 | 0.0088 | 0.0140 | 0.5287 |
| rs77149268 | T | C | 0.1051 | 0.0066 | 0.0006 | 5.82E-25 | 0.0670 | -0.0181 | 0.0259 | 0.4839 |
| rs7726961 | T | C | 0.4261 | -0.0023 | 0.0004 | 6.85E-09 | 0.4067 | 0.0005 | 0.0132 | 0.9727 |
| rs77282753 | C | T | 0.0480 | -0.0069 | 0.0009 | 4.06E-14 | 0.0533 | 0.0165 | 0.0286 | 0.5633 |
| rs7860558 | C | T | 0.7013 | -0.0029 | 0.0004 | 3.13E-11 | 0.7081 | -0.0121 | 0.0143 | 0.3994 |
| rs79472736 | A | G | 0.0354 | -0.0070 | 0.0011 | 3.45E-11 | 0.0335 | -0.0086 | 0.0362 | 0.8132 |
| rs805288 | T | C | 0.2491 | 0.0025 | 0.0005 | 4.98E-08 | 0.3847 | -0.0268 | 0.0148 | 0.0689 |
| rs8075449 | T | C | 0.2590 | -0.0028 | 0.0004 | 5.03E-10 | 0.3388 | -0.0089 | 0.0137 | 0.5156 |
| rs838717 | A | G | 0.5656 | 0.0049 | 0.0004 | 3.04E-35 | 0.5842 | -0.0135 | 0.0131 | 0.3018 |
| rs872386 | A | G | 0.5310 | -0.0039 | 0.0004 | 2.60E-23 | 0.5868 | 0.0168 | 0.0132 | 0.2014 |
| rs896788 | T | C | 0.1552 | 0.0042 | 0.0005 | 6.52E-15 | 0.2141 | -0.0086 | 0.0158 | 0.5836 |
| rs912981 | C | A | 0.5810 | -0.0026 | 0.0004 | 3.22E-11 | 0.6546 | -0.0106 | 0.0137 | 0.4388 |
| rs9574586 | C | T | 0.3311 | -0.0023 | 0.0004 | 2.35E-08 | 0.3286 | -0.0042 | 0.0137 | 0.7617 |
| rs9689096 | C | A | 0.0639 | -0.0070 | 0.0008 | 4.31E-18 | 0.0323 | 0.1276 | 0.0370 | 0.0006 |
| rs9745989 | C | T | 0.6281 | 0.0025 | 0.0004 | 9.31E-10 | 0.6891 | 0.0182 | 0.0141 | 0.1983 |

The F-statistics for phosphate was 34.23. **Abbreviations: MR,** Mendelian randomization; **CHD**, coronary heart diseases; **SNP**, single-nucleotide polymorphism; **EA**, effect allele; **OA**, other allele; **EAF**, effect allele frequency; **SE**, standard error.

**Table S3-2: Characteristics of instrumental variables for phosphate and HF in the MR analysis**

| **SNP** | **EA** | **OA** | **Exposure** | | | | **HF** | | | |
| --- | --- | --- | --- | --- | --- | --- | --- | --- | --- | --- |
|  |  |  | **EAF** | **Beta** | **SE** | **pval** | **EAF** | **Beta** | **SE** | **pval** |
| rs10051765 | C | T | 0.3324 | -0.0082 | 0.0004 | 3.03E-87 | 0.0083 | -0.0048 | 0.0083 | 0.5669 |
| rs10401230 | G | A | 0.4937 | -0.0022 | 0.0004 | 9.47E-09 | 0.0082 | 0.0003 | 0.0082 | 0.9686 |
| rs10743976 | C | A | 0.8512 | 0.0067 | 0.0005 | 7.00E-34 | 0.0114 | -0.0061 | 0.0114 | 0.5903 |
| rs11144134 | C | T | 0.0845 | 0.0043 | 0.0007 | 7.16E-10 | 0.0151 | 0.0151 | 0.0151 | 0.3161 |
| rs113487566 | T | C | 0.0254 | -0.0102 | 0.0013 | 5.87E-16 | 0.0265 | -0.0468 | 0.0265 | 0.0775 |
| rs11684211 | A | G | 0.3533 | 0.0026 | 0.0004 | 3.81E-10 | 0.0082 | -0.0031 | 0.0082 | 0.7074 |
| rs11801716 | T | C | 0.0462 | -0.0054 | 0.0009 | 9.47E-09 | 0.0176 | -0.0132 | 0.0176 | 0.4541 |
| rs12044944 | T | C | 0.1925 | 0.0029 | 0.0005 | 4.29E-09 | 0.0100 | -0.0002 | 0.0100 | 0.9816 |
| rs12059422 | C | T | 0.0230 | 0.0146 | 0.0013 | 3.32E-29 | 0.0244 | 0.0187 | 0.0244 | 0.4451 |
| rs12132412 | G | A | 0.3884 | 0.0174 | 0.0004 | 1.00E-200 | 0.0083 | -0.0095 | 0.0083 | 0.2548 |
| rs12518871 | T | C | 0.4095 | -0.0025 | 0.0004 | 2.57E-10 | 0.0080 | 0.0055 | 0.0080 | 0.4894 |
| rs12710029 | C | T | 0.7762 | -0.0029 | 0.0005 | 7.06E-10 | 0.0093 | -0.0136 | 0.0093 | 0.1426 |
| rs12727188 | G | T | 0.0894 | -0.0054 | 0.0007 | 6.82E-15 | 0.0131 | -0.0099 | 0.0131 | 0.4502 |
| rs1286070 | C | T | 0.1721 | -0.0041 | 0.0005 | 2.43E-15 | 0.0104 | -0.0092 | 0.0104 | 0.3772 |
| rs12902894 | C | A | 0.4580 | 0.0044 | 0.0004 | 8.14E-29 | 0.0079 | -0.0036 | 0.0079 | 0.6486 |
| rs12941512 | T | C | 0.4422 | -0.0032 | 0.0004 | 2.07E-16 | 0.0079 | -0.0020 | 0.0079 | 0.8049 |
| rs12985272 | A | G | 0.0494 | 0.0064 | 0.0009 | 4.69E-12 | 0.0190 | -0.0133 | 0.0190 | 0.4831 |
| rs13232861 | G | A | 0.8125 | 0.0031 | 0.0005 | 3.76E-10 | 0.0102 | 0.0124 | 0.0102 | 0.2245 |
| rs139796809 | T | C | 0.5570 | -0.0028 | 0.0004 | 1.53E-11 | 0.0141 | 0.0128 | 0.0141 | 0.3621 |
| rs144214505 | T | C | 0.0416 | -0.0057 | 0.0010 | 7.05E-09 | 0.0225 | 0.0008 | 0.0225 | 0.9714 |
| rs145978363 | G | A | 0.0301 | -0.0072 | 0.0012 | 1.89E-09 | 0.0251 | 0.0038 | 0.0251 | 0.8799 |
| rs1468908 | G | A | 0.4920 | 0.0050 | 0.0004 | 6.28E-37 | 0.0078 | -0.0230 | 0.0078 | 0.0033 |
| rs1483842 | C | T | 0.4808 | -0.0028 | 0.0004 | 1.42E-12 | 0.0078 | 0.0087 | 0.0078 | 0.2629 |
| rs1570603 | A | G | 0.5720 | -0.0022 | 0.0004 | 3.69E-08 | 0.0080 | -0.0060 | 0.0080 | 0.4529 |
| rs17036160 | T | C | 0.1180 | -0.0040 | 0.0006 | 5.67E-11 | 0.0119 | -0.0094 | 0.0119 | 0.4286 |
| rs17288460 | A | G | 0.1803 | -0.0033 | 0.0005 | 7.84E-11 | 0.0119 | -0.0027 | 0.0119 | 0.8195 |
| rs17884869 | A | G | 0.0250 | 0.0079 | 0.0013 | 2.58E-10 | 0.0239 | 0.0020 | 0.0239 | 0.9327 |
| rs1889208 | A | G | 0.3359 | 0.0033 | 0.0004 | 3.76E-15 | 0.0083 | 0.0041 | 0.0083 | 0.6206 |
| rs209961 | C | T | 0.7219 | 0.0037 | 0.0004 | 1.38E-17 | 0.0088 | 0.0117 | 0.0088 | 0.1866 |
| rs2303917 | A | G | 0.1077 | -0.0037 | 0.0006 | 6.18E-09 | 0.0126 | 0.0007 | 0.0126 | 0.9571 |
| rs2909212 | G | A | 0.2966 | 0.0024 | 0.0004 | 2.31E-08 | 0.0086 | -0.0011 | 0.0086 | 0.8948 |
| rs2959652 | T | G | 0.9080 | -0.0037 | 0.0007 | 3.13E-08 | 0.0130 | 0.0011 | 0.0130 | 0.9321 |
| rs296847 | T | G | 0.6643 | 0.0024 | 0.0004 | 8.87E-09 | 0.0083 | 0.0042 | 0.0083 | 0.6082 |
| rs2982572 | T | C | 0.4208 | -0.0041 | 0.0004 | 1.83E-25 | 0.0079 | -0.0181 | 0.0079 | 0.0216 |
| rs308032 | T | G | 0.2368 | -0.0029 | 0.0005 | 1.03E-09 | 0.0100 | 0.0027 | 0.0100 | 0.7883 |
| rs3091842 | A | G | 0.0441 | -0.0086 | 0.0010 | 1.49E-18 | 0.0176 | -0.0305 | 0.0176 | 0.0837 |
| rs339969 | A | C | 0.6148 | 0.0027 | 0.0004 | 2.35E-11 | 0.0081 | -0.0019 | 0.0081 | 0.8176 |
| rs35026779 | T | C | 0.0997 | -0.0042 | 0.0007 | 9.54E-11 | 0.0139 | -0.0037 | 0.0139 | 0.7925 |
| rs35666081 | A | G | 0.4002 | 0.0022 | 0.0004 | 4.52E-08 | 0.0081 | 0.0001 | 0.0081 | 0.9913 |
| rs3757677 | G | A | 0.3721 | 0.0030 | 0.0004 | 6.84E-14 | 0.0082 | -0.0085 | 0.0082 | 0.2994 |
| rs3813498 | T | C | 0.8128 | 0.0051 | 0.0005 | 3.42E-24 | 0.0100 | 0.0067 | 0.0100 | 0.5004 |
| rs4072537 | C | T | 0.6811 | -0.0023 | 0.0004 | 3.95E-08 | 0.0098 | 0.0159 | 0.0098 | 0.1069 |
| rs4077450 | T | G | 0.8280 | -0.0042 | 0.0005 | 1.15E-15 | 0.0104 | 0.0021 | 0.0104 | 0.8402 |
| rs41278174 | A | G | 0.0273 | 0.0170 | 0.0012 | 1.31E-45 | 0.0269 | -0.0128 | 0.0269 | 0.6351 |
| rs4509682 | A | G | 0.3919 | -0.0031 | 0.0004 | 5.48E-15 | 0.0080 | -0.0112 | 0.0080 | 0.1623 |
| rs453639 | A | C | 0.6405 | -0.0107 | 0.0004 | 2.10E-150 | 0.0084 | 0.0030 | 0.0084 | 0.7240 |
| rs4706554 | A | G | 0.4763 | 0.0029 | 0.0004 | 2.22E-13 | 0.0078 | 0.0166 | 0.0078 | 0.0341 |
| rs4759844 | G | A | 0.3509 | 0.0038 | 0.0004 | 1.22E-20 | 0.0082 | 0.0042 | 0.0082 | 0.6078 |
| rs4795607 | T | C | 0.6140 | 0.0024 | 0.0004 | 1.58E-09 | 0.0081 | -0.0052 | 0.0081 | 0.5204 |
| rs4855880 | A | G | 0.5164 | -0.0022 | 0.0004 | 4.18E-08 | 0.0121 | 0.0165 | 0.0121 | 0.1711 |
| rs4869745 | T | C | 0.2871 | 0.0031 | 0.0004 | 6.73E-13 | 0.0087 | 0.0130 | 0.0087 | 0.1343 |
| rs4982711 | G | T | 0.5710 | 0.0023 | 0.0004 | 1.05E-08 | 0.0116 | 0.0108 | 0.0116 | 0.3550 |
| rs5435 | C | T | 0.6121 | -0.0022 | 0.0004 | 4.08E-08 | 0.0081 | -0.0005 | 0.0081 | 0.9498 |
| rs55923451 | A | G | 0.1791 | -0.0034 | 0.0005 | 5.39E-11 | 0.0101 | -0.0097 | 0.0101 | 0.3405 |
| rs56078331 | T | C | 0.0544 | 0.0084 | 0.0009 | 2.07E-22 | 0.0174 | 0.0018 | 0.0174 | 0.9170 |
| rs56328339 | T | C | 0.1628 | 0.0052 | 0.0005 | 1.05E-22 | 0.0106 | 0.0101 | 0.0106 | 0.3376 |
| rs596940 | C | T | 0.0716 | 0.0052 | 0.0008 | 1.16E-11 | 0.0153 | 0.0137 | 0.0153 | 0.3725 |
| rs60447213 | T | G | 0.0699 | -0.0299 | 0.0008 | 1.00E-200 | 0.0145 | -0.0147 | 0.0145 | 0.3108 |
| rs6136492 | T | C | 0.2259 | -0.0027 | 0.0005 | 5.88E-09 | 0.0093 | 0.0100 | 0.0093 | 0.2825 |
| rs61870274 | A | C | 0.0781 | 0.0059 | 0.0007 | 7.92E-16 | 0.0142 | 0.0049 | 0.0142 | 0.7324 |
| rs61909254 | C | T | 0.8598 | 0.0101 | 0.0006 | 8.49E-72 | 0.0114 | -0.0067 | 0.0114 | 0.5544 |
| rs62289280 | C | T | 0.3053 | -0.0029 | 0.0004 | 1.09E-11 | 0.0085 | -0.0053 | 0.0085 | 0.5351 |
| rs62383568 | C | T | 0.3497 | -0.0024 | 0.0004 | 3.35E-09 | 0.0084 | -0.0017 | 0.0084 | 0.8416 |
| rs6751217 | T | C | 0.6316 | 0.0029 | 0.0004 | 6.03E-13 | 0.0081 | -0.0070 | 0.0081 | 0.3863 |
| rs6780593 | A | G | 0.1528 | -0.0045 | 0.0005 | 8.55E-17 | 0.0109 | -0.0100 | 0.0109 | 0.3608 |
| rs6841258 | T | C | 0.1663 | 0.0031 | 0.0005 | 5.29E-09 | 0.0110 | -0.0110 | 0.0110 | 0.3189 |
| rs6911602 | A | G | 0.1318 | 0.0082 | 0.0006 | 5.07E-46 | 0.0112 | 0.0079 | 0.0112 | 0.4782 |
| rs6983239 | T | G | 0.2186 | 0.0039 | 0.0005 | 3.52E-16 | 0.0096 | -0.0043 | 0.0096 | 0.6509 |
| rs7017252 | T | C | 0.3830 | 0.0022 | 0.0004 | 3.53E-08 | 0.0081 | 0.0090 | 0.0081 | 0.2672 |
| rs7033278 | T | C | 0.2930 | 0.0035 | 0.0004 | 5.96E-16 | 0.0085 | -0.0052 | 0.0085 | 0.5456 |
| rs706300 | G | A | 0.8575 | -0.0054 | 0.0006 | 2.96E-22 | 0.0115 | 0.0051 | 0.0115 | 0.6542 |
| rs71413981 | A | G | 0.1637 | -0.0031 | 0.0005 | 6.93E-09 | 0.0112 | -0.0018 | 0.0112 | 0.8727 |
| rs72660359 | T | G | 0.0373 | 0.0148 | 0.0010 | 1.30E-45 | 0.0209 | 0.0031 | 0.0209 | 0.8819 |
| rs72716022 | T | C | 0.2021 | -0.0033 | 0.0005 | 1.09E-10 | 0.0101 | 0.0139 | 0.0101 | 0.1674 |
| rs72974112 | T | C | 0.2373 | -0.0029 | 0.0005 | 4.78E-10 | 0.0093 | -0.0031 | 0.0093 | 0.7404 |
| rs7324259 | T | G | 0.1259 | 0.0037 | 0.0006 | 4.73E-10 | 0.0125 | 0.0063 | 0.0125 | 0.6168 |
| rs7529925 | T | C | 0.7383 | 0.0031 | 0.0004 | 7.01E-12 | 0.0088 | 0.0007 | 0.0088 | 0.9398 |
| rs7555660 | T | C | 0.0853 | 0.0090 | 0.0007 | 4.91E-38 | 0.0146 | 0.0095 | 0.0146 | 0.5134 |
| rs7625643 | G | A | 0.4482 | 0.0026 | 0.0004 | 7.39E-11 | 0.0118 | -0.0061 | 0.0118 | 0.6062 |
| rs76819459 | G | A | 0.3270 | -0.0051 | 0.0004 | 1.15E-34 | 0.0084 | 0.0028 | 0.0084 | 0.7422 |
| rs77149268 | T | C | 0.1051 | 0.0066 | 0.0006 | 5.82E-25 | 0.0131 | -0.0065 | 0.0131 | 0.6212 |
| rs7726961 | T | C | 0.4261 | -0.0023 | 0.0004 | 6.85E-09 | 0.0079 | 0.0048 | 0.0079 | 0.5469 |
| rs77282753 | C | T | 0.0480 | -0.0069 | 0.0009 | 4.06E-14 | 0.0180 | 0.0149 | 0.0180 | 0.4094 |
| rs7860558 | C | T | 0.7013 | -0.0029 | 0.0004 | 3.13E-11 | 0.0088 | 0.0019 | 0.0088 | 0.8275 |
| rs79472736 | A | G | 0.0354 | -0.0070 | 0.0011 | 3.45E-11 | 0.0244 | -0.0053 | 0.0244 | 0.8290 |
| rs805288 | T | C | 0.2491 | 0.0025 | 0.0005 | 4.98E-08 | 0.0091 | -0.0189 | 0.0091 | 0.0374 |
| rs8075449 | T | C | 0.2590 | -0.0028 | 0.0004 | 5.03E-10 | 0.0091 | -0.0001 | 0.0091 | 0.9911 |
| rs838717 | A | G | 0.5656 | 0.0049 | 0.0004 | 3.04E-35 | 0.0078 | -0.0114 | 0.0078 | 0.1441 |
| rs872386 | A | G | 0.5310 | -0.0039 | 0.0004 | 2.60E-23 | 0.0079 | 0.0078 | 0.0079 | 0.3259 |
| rs878919 | C | T | 0.3970 | 0.0032 | 0.0004 | 3.10E-15 | 0.0079 | -0.0034 | 0.0079 | 0.6724 |
| rs896788 | T | C | 0.1552 | 0.0042 | 0.0005 | 6.52E-15 | 0.0107 | 0.0104 | 0.0107 | 0.3317 |
| rs912981 | C | A | 0.5810 | -0.0026 | 0.0004 | 3.22E-11 | 0.0079 | -0.0113 | 0.0079 | 0.1558 |
| rs9574586 | C | T | 0.3311 | -0.0023 | 0.0004 | 2.35E-08 | 0.0082 | 0.0110 | 0.0082 | 0.1789 |
| rs9689096 | C | A | 0.0639 | -0.0070 | 0.0008 | 4.31E-18 | 0.0199 | 0.0203 | 0.0199 | 0.3078 |
| rs9745989 | C | T | 0.6281 | 0.0025 | 0.0004 | 9.31E-10 | 0.0087 | 0.0056 | 0.0087 | 0.5221 |

The F-statistics for phosphate was 35.77. Abbreviations: **MR,** Mendelian randomization; **HF**, heart failure; **SNP**, single-nucleotide polymorphism; **EA**, effect allele; **OA**, other allele; **EAF**, effect allele frequency; **SE**, standard error.

**Table S3-3: Characteristics of instrumental variables for phosphate and AF in the MR analysis**

| **SNP** | **EA** | **OA** | **Exposure** | | | | **AF** | | | |
| --- | --- | --- | --- | --- | --- | --- | --- | --- | --- | --- |
|  |  |  | **EAF** | **Beta** | **SE** | **pval** | **EAF** | **Beta** | **SE** | **pval** |
| rs10051765 | C | T | 0.3324 | -0.0082 | 0.0004 | 3.03E-87 | 0.3511 | 0.0041 | 0.0071 | 0.5650 |
| rs10279171 | T | A | 0.7941 | 0.0029 | 0.0005 | 2.04E-09 | 0.7705 | 0.0085 | 0.0082 | 0.3006 |
| rs10282449 | A | T | 0.1663 | 0.0037 | 0.0005 | 2.15E-12 | 0.1756 | 0.0055 | 0.0089 | 0.5348 |
| rs10401230 | G | A | 0.4937 | -0.0022 | 0.0004 | 9.47E-09 | 0.4780 | 0.0021 | 0.0071 | 0.7643 |
| rs10743976 | C | A | 0.8512 | 0.0067 | 0.0005 | 7.00E-34 | 0.8393 | -0.0011 | 0.0098 | 0.9092 |
| rs11144134 | C | T | 0.0845 | 0.0043 | 0.0007 | 7.16E-10 | 0.0789 | 0.0035 | 0.0128 | 0.7825 |
| rs111723017 | C | G | 0.0875 | 0.0053 | 0.0007 | 1.89E-14 | 0.0980 | -0.0109 | 0.0110 | 0.3179 |
| rs113487566 | T | C | 0.0254 | -0.0102 | 0.0013 | 5.87E-16 | 0.0240 | -0.0150 | 0.0247 | 0.5444 |
| rs11684211 | A | G | 0.3533 | 0.0026 | 0.0004 | 3.81E-10 | 0.3485 | -0.0069 | 0.0070 | 0.3216 |
| rs11801716 | T | C | 0.0462 | -0.0054 | 0.0009 | 9.47E-09 | 0.0515 | -0.0051 | 0.0150 | 0.7307 |
| rs12044944 | T | C | 0.1925 | 0.0029 | 0.0005 | 4.29E-09 | 0.1952 | 0.0015 | 0.0085 | 0.8559 |
| rs12059422 | C | T | 0.0230 | 0.0146 | 0.0013 | 3.32E-29 | 0.0255 | 0.0429 | 0.0216 | 0.0466 |
| rs12132412 | G | A | 0.3884 | 0.0174 | 0.0004 | 1.00E-200 | 0.3877 | -0.0109 | 0.0071 | 0.1248 |
| rs12409158 | C | G | 0.0319 | -0.0064 | 0.0011 | 1.70E-08 | 0.0276 | -0.0364 | 0.0238 | 0.1261 |
| rs12518871 | T | C | 0.4095 | -0.0025 | 0.0004 | 2.57E-10 | 0.4089 | 0.0025 | 0.0068 | 0.7142 |
| rs12710029 | C | T | 0.7762 | -0.0029 | 0.0005 | 7.06E-10 | 0.7524 | -0.0018 | 0.0081 | 0.8239 |
| rs12727188 | G | T | 0.0894 | -0.0054 | 0.0007 | 6.82E-15 | 0.1065 | 0.0076 | 0.0112 | 0.4965 |
| rs12770024 | G | C | 0.1936 | 0.0037 | 0.0005 | 4.92E-14 | 0.1909 | 0.0020 | 0.0086 | 0.8129 |
| rs1286070 | C | T | 0.1721 | -0.0041 | 0.0005 | 2.43E-15 | 0.1701 | 0.0036 | 0.0087 | 0.6815 |
| rs12902894 | C | A | 0.4580 | 0.0044 | 0.0004 | 8.14E-29 | 0.4563 | 0.0020 | 0.0067 | 0.7646 |
| rs12941512 | T | C | 0.4422 | -0.0032 | 0.0004 | 2.07E-16 | 0.4497 | 0.0047 | 0.0068 | 0.4865 |
| rs12985272 | A | G | 0.0494 | 0.0064 | 0.0009 | 4.69E-12 | 0.0517 | 0.0140 | 0.0168 | 0.4026 |
| rs13232861 | G | A | 0.8125 | 0.0031 | 0.0005 | 3.76E-10 | 0.7922 | -0.0059 | 0.0085 | 0.4867 |
| rs13273161 | T | A | 0.3177 | 0.0030 | 0.0004 | 9.94E-13 | 0.3144 | -0.0181 | 0.0072 | 0.0124 |
| rs138278885 | CA | C | 0.1007 | -0.0057 | 0.0007 | 3.68E-18 | 0.1015 | -0.0041 | 0.0217 | 0.8494 |
| rs139796809 | T | C | 0.5570 | -0.0028 | 0.0004 | 1.53E-11 | 0.5511 | -0.0056 | 0.0127 | 0.6629 |
| rs144214505 | T | C | 0.0416 | -0.0057 | 0.0010 | 7.05E-09 | 0.0394 | -0.0068 | 0.0193 | 0.7260 |
| rs145330264 | C | A | 0.0054 | -0.0321 | 0.0028 | 5.75E-30 | 0.0401 | 0.0073 | 0.0199 | 0.7152 |
| rs145978363 | G | A | 0.0301 | -0.0072 | 0.0012 | 1.89E-09 | 0.0289 | 0.0186 | 0.0221 | 0.3986 |
| rs1468908 | G | A | 0.4920 | 0.0050 | 0.0004 | 6.28E-37 | 0.4753 | -0.0202 | 0.0067 | 0.0024 |
| rs1483842 | C | T | 0.4808 | -0.0028 | 0.0004 | 1.42E-12 | 0.4908 | -0.0007 | 0.0067 | 0.9187 |
| rs1570603 | A | G | 0.5720 | -0.0022 | 0.0004 | 3.69E-08 | 0.5695 | 0.0091 | 0.0067 | 0.1760 |
| rs17036160 | T | C | 0.1180 | -0.0040 | 0.0006 | 5.67E-11 | 0.1230 | 0.0344 | 0.0103 | 0.0008 |
| rs17288460 | A | G | 0.1803 | -0.0033 | 0.0005 | 7.84E-11 | 0.1817 | 0.0201 | 0.0096 | 0.0360 |
| rs17884869 | A | G | 0.0250 | 0.0079 | 0.0013 | 2.58E-10 | 0.0383 | -0.0328 | 0.0207 | 0.1134 |
| rs1889208 | A | G | 0.3359 | 0.0033 | 0.0004 | 3.76E-15 | 0.3416 | -0.0115 | 0.0071 | 0.1043 |
| rs209961 | C | T | 0.7219 | 0.0037 | 0.0004 | 1.38E-17 | 0.7109 | 0.0071 | 0.0075 | 0.3470 |
| rs2177470 | A | T | 0.6819 | -0.0027 | 0.0004 | 2.60E-10 | 0.6806 | 0.0116 | 0.0073 | 0.1132 |
| rs2303917 | A | G | 0.1077 | -0.0037 | 0.0006 | 6.18E-09 | 0.1194 | -0.0254 | 0.0106 | 0.0162 |
| rs2420055 | C | T | 0.8426 | -0.0035 | 0.0005 | 4.21E-11 | 0.8182 | 0.0047 | 0.0091 | 0.6095 |
| rs273587 | T | A | 0.6778 | -0.0030 | 0.0004 | 7.53E-13 | 0.6818 | -0.0156 | 0.0072 | 0.0294 |
| rs28542318 | T | A | 0.8344 | 0.0032 | 0.0005 | 1.75E-09 | 0.8234 | -0.0061 | 0.0102 | 0.5460 |
| rs2909212 | G | A | 0.2966 | 0.0024 | 0.0004 | 2.31E-08 | 0.3002 | 0.0011 | 0.0073 | 0.8768 |
| rs2959652 | T | G | 0.9080 | -0.0037 | 0.0007 | 3.13E-08 | 0.8772 | 0.0033 | 0.0108 | 0.7596 |
| rs296847 | T | G | 0.6643 | 0.0024 | 0.0004 | 8.87E-09 | 0.6554 | 0.0003 | 0.0071 | 0.9632 |
| rs2970818 | A | T | 0.1038 | 0.0206 | 0.0006 | 1.00E-200 | 0.1112 | -0.0053 | 0.0109 | 0.6251 |
| rs2982572 | T | C | 0.4208 | -0.0041 | 0.0004 | 1.83E-25 | 0.4284 | -0.0239 | 0.0067 | 0.0004 |
| rs308032 | T | G | 0.2368 | -0.0029 | 0.0005 | 1.03E-09 | 0.2208 | 0.0027 | 0.0085 | 0.7493 |
| rs3091842 | A | G | 0.0441 | -0.0086 | 0.0010 | 1.49E-18 | 0.0526 | -0.0220 | 0.0158 | 0.1648 |
| rs3213473 | A | T | 0.0343 | 0.0132 | 0.0011 | 6.75E-31 | 0.0335 | -0.0204 | 0.0263 | 0.4376 |
| rs339969 | A | C | 0.6148 | 0.0027 | 0.0004 | 2.35E-11 | 0.6191 | 0.0099 | 0.0069 | 0.1508 |
| rs35026779 | T | C | 0.0997 | -0.0042 | 0.0007 | 9.54E-11 | 0.0915 | 0.0266 | 0.0114 | 0.0202 |
| rs35666081 | A | G | 0.4002 | 0.0022 | 0.0004 | 4.52E-08 | 0.3877 | 0.0023 | 0.0068 | 0.7328 |
| rs3757677 | G | A | 0.3721 | 0.0030 | 0.0004 | 6.84E-14 | 0.3554 | -0.0088 | 0.0070 | 0.2083 |
| rs3813498 | T | C | 0.8128 | 0.0051 | 0.0005 | 3.42E-24 | 0.7985 | 0.0013 | 0.0085 | 0.8785 |
| rs4072537 | C | T | 0.6811 | -0.0023 | 0.0004 | 3.95E-08 | 0.6847 | 0.0009 | 0.0082 | 0.9143 |
| rs4077450 | T | G | 0.8280 | -0.0042 | 0.0005 | 1.15E-15 | 0.8057 | -0.0133 | 0.0087 | 0.1287 |
| rs41278174 | A | G | 0.0273 | 0.0170 | 0.0012 | 1.31E-45 | 0.0257 | -0.0116 | 0.0235 | 0.6231 |
| rs4509682 | A | G | 0.3919 | -0.0031 | 0.0004 | 5.48E-15 | 0.3874 | -0.0307 | 0.0068 | 0.0000 |
| rs453639 | A | C | 0.6405 | -0.0107 | 0.0004 | 2.10E-150 | 0.6316 | 0.0116 | 0.0072 | 0.1065 |
| rs4706554 | A | G | 0.4763 | 0.0029 | 0.0004 | 2.22E-13 | 0.4643 | -0.0132 | 0.0067 | 0.0474 |
| rs4759844 | G | A | 0.3509 | 0.0038 | 0.0004 | 1.22E-20 | 0.3556 | -0.0128 | 0.0070 | 0.0680 |
| rs4795607 | T | C | 0.6140 | 0.0024 | 0.0004 | 1.58E-09 | 0.6049 | 0.0023 | 0.0069 | 0.7362 |
| rs4855880 | A | G | 0.5164 | -0.0022 | 0.0004 | 4.18E-08 | 0.5112 | -0.0054 | 0.0107 | 0.6148 |
| rs4869745 | T | C | 0.2871 | 0.0031 | 0.0004 | 6.73E-13 | 0.2831 | 0.0125 | 0.0074 | 0.0901 |
| rs4938642 | C | G | 0.0740 | 0.0041 | 0.0007 | 4.74E-08 | 0.0672 | 0.0086 | 0.0136 | 0.5286 |
| rs4982711 | G | T | 0.5710 | 0.0023 | 0.0004 | 1.05E-08 | 0.5563 | -0.0025 | 0.0092 | 0.7816 |
| rs5435 | C | T | 0.6121 | -0.0022 | 0.0004 | 4.08E-08 | 0.6160 | -0.0101 | 0.0070 | 0.1461 |
| rs55923451 | A | G | 0.1791 | -0.0034 | 0.0005 | 5.39E-11 | 0.1812 | 0.0044 | 0.0087 | 0.6154 |
| rs56078331 | T | C | 0.0544 | 0.0084 | 0.0009 | 2.07E-22 | 0.0545 | -0.0226 | 0.0151 | 0.1359 |
| rs56328339 | T | C | 0.1628 | 0.0052 | 0.0005 | 1.05E-22 | 0.1622 | 0.0034 | 0.0092 | 0.7134 |
| rs596940 | C | T | 0.0716 | 0.0052 | 0.0008 | 1.16E-11 | 0.0719 | 0.0071 | 0.0129 | 0.5816 |
| rs60447213 | T | G | 0.0699 | -0.0299 | 0.0008 | 1.00E-200 | 0.0755 | -0.0118 | 0.0126 | 0.3467 |
| rs6136492 | T | C | 0.2259 | -0.0027 | 0.0005 | 5.88E-09 | 0.2316 | -0.0129 | 0.0079 | 0.1029 |
| rs61870274 | A | C | 0.0781 | 0.0059 | 0.0007 | 7.92E-16 | 0.0810 | 0.0114 | 0.0122 | 0.3467 |
| rs61909254 | C | T | 0.8598 | 0.0101 | 0.0006 | 8.49E-72 | 0.8440 | 0.0018 | 0.0097 | 0.8519 |
| rs62289280 | C | T | 0.3053 | -0.0029 | 0.0004 | 1.09E-11 | 0.2966 | -0.0163 | 0.0073 | 0.0254 |
| rs62383568 | C | T | 0.3497 | -0.0024 | 0.0004 | 3.35E-09 | 0.3331 | 0.0142 | 0.0071 | 0.0463 |
| rs6751217 | T | C | 0.6316 | 0.0029 | 0.0004 | 6.03E-13 | 0.6221 | -0.0112 | 0.0068 | 0.1023 |
| rs6780593 | A | G | 0.1528 | -0.0045 | 0.0005 | 8.55E-17 | 0.1525 | -0.0010 | 0.0093 | 0.9137 |
| rs6841258 | T | C | 0.1663 | 0.0031 | 0.0005 | 5.29E-09 | 0.1590 | -0.0009 | 0.0092 | 0.9214 |
| rs6911602 | A | G | 0.1318 | 0.0082 | 0.0006 | 5.07E-46 | 0.1373 | 0.0145 | 0.0096 | 0.1307 |
| rs6983239 | T | G | 0.2186 | 0.0039 | 0.0005 | 3.52E-16 | 0.2153 | -0.0094 | 0.0081 | 0.2437 |
| rs7017252 | T | C | 0.3830 | 0.0022 | 0.0004 | 3.53E-08 | 0.3787 | 0.0111 | 0.0069 | 0.1058 |
| rs7033278 | T | C | 0.2930 | 0.0035 | 0.0004 | 5.96E-16 | 0.3044 | 0.0007 | 0.0072 | 0.9235 |
| rs706300 | G | A | 0.8575 | -0.0054 | 0.0006 | 2.96E-22 | 0.8400 | 0.0108 | 0.0096 | 0.2636 |
| rs71368113 | G | C | 0.0709 | -0.0045 | 0.0008 | 4.44E-08 | 0.0772 | -0.0178 | 0.0154 | 0.2468 |
| rs71413981 | A | G | 0.1637 | -0.0031 | 0.0005 | 6.93E-09 | 0.1548 | 0.0134 | 0.0095 | 0.1618 |
| rs72660359 | T | G | 0.0373 | 0.0148 | 0.0010 | 1.30E-45 | 0.0412 | 0.0217 | 0.0182 | 0.2326 |
| rs72716022 | T | C | 0.2021 | -0.0033 | 0.0005 | 1.09E-10 | 0.2009 | -0.0098 | 0.0087 | 0.2587 |
| rs72974112 | T | C | 0.2373 | -0.0029 | 0.0005 | 4.78E-10 | 0.2525 | 0.0035 | 0.0078 | 0.6531 |
| rs7324259 | T | G | 0.1259 | 0.0037 | 0.0006 | 4.73E-10 | 0.1259 | 0.0043 | 0.0106 | 0.6845 |
| rs74551305 | C | T | 0.0560 | 0.0074 | 0.0009 | 1.23E-16 | NA | -0.0266 | 0.0306 | 0.3847 |
| rs7529925 | T | C | 0.7383 | 0.0031 | 0.0004 | 7.01E-12 | 0.7260 | -0.0047 | 0.0076 | 0.5318 |
| rs7555660 | T | C | 0.0853 | 0.0090 | 0.0007 | 4.91E-38 | 0.0845 | 0.0149 | 0.0123 | 0.2260 |
| rs7625643 | G | A | 0.4482 | 0.0026 | 0.0004 | 7.39E-11 | 0.4741 | 0.0338 | 0.0101 | 0.0009 |
| rs76527351 | G | A | 0.2257 | -0.0044 | 0.0005 | 7.59E-21 | 0.1757 | 0.0647 | 0.0219 | 0.0032 |
| rs7672820 | C | G | 0.1863 | 0.0043 | 0.0005 | 6.26E-18 | 0.1797 | -0.0029 | 0.0087 | 0.7372 |
| rs76819459 | G | A | 0.3270 | -0.0051 | 0.0004 | 1.15E-34 | 0.3236 | -0.0045 | 0.0071 | 0.5261 |
| rs77149268 | T | C | 0.1051 | 0.0066 | 0.0006 | 5.82E-25 | 0.1006 | -0.0206 | 0.0112 | 0.0648 |
| rs7726961 | T | C | 0.4261 | -0.0023 | 0.0004 | 6.85E-09 | 0.4250 | -0.0164 | 0.0067 | 0.0149 |
| rs77282753 | C | T | 0.0480 | -0.0069 | 0.0009 | 4.06E-14 | 0.0493 | -0.0067 | 0.0152 | 0.6586 |
| rs7860558 | C | T | 0.7013 | -0.0029 | 0.0004 | 3.13E-11 | 0.6878 | -0.0007 | 0.0073 | 0.9204 |
| rs79472736 | A | G | 0.0354 | -0.0070 | 0.0011 | 3.45E-11 | 0.0339 | 0.0194 | 0.0209 | 0.3537 |
| rs805288 | T | C | 0.2491 | 0.0025 | 0.0005 | 4.98E-08 | 0.2654 | -0.0203 | 0.0081 | 0.0117 |
| rs8075449 | T | C | 0.2590 | -0.0028 | 0.0004 | 5.03E-10 | 0.2501 | -0.0025 | 0.0076 | 0.7406 |
| rs838717 | A | G | 0.5656 | 0.0049 | 0.0004 | 3.04E-35 | 0.5500 | -0.0042 | 0.0067 | 0.5332 |
| rs872386 | A | G | 0.5310 | -0.0039 | 0.0004 | 2.60E-23 | 0.5378 | 0.0250 | 0.0067 | 0.0002 |
| rs878919 | C | T | 0.3970 | 0.0032 | 0.0004 | 3.10E-15 | 0.4089 | -0.0120 | 0.0068 | 0.0774 |
| rs896788 | T | C | 0.1552 | 0.0042 | 0.0005 | 6.52E-15 | 0.1588 | 0.0107 | 0.0090 | 0.2332 |
| rs912981 | C | A | 0.5810 | -0.0026 | 0.0004 | 3.22E-11 | 0.5866 | 0.0067 | 0.0067 | 0.3181 |
| rs9574586 | C | T | 0.3311 | -0.0023 | 0.0004 | 2.35E-08 | 0.3483 | 0.0007 | 0.0070 | 0.9158 |
| rs9689096 | C | A | 0.0639 | -0.0070 | 0.0008 | 4.31E-18 | 0.0574 | 0.0610 | 0.0148 | 0.0000 |
| rs9745989 | C | T | 0.6281 | 0.0025 | 0.0004 | 9.31E-10 | 0.6420 | 0.0114 | 0.0074 | 0.1237 |

The F-statistics for phosphate was 33.52. **Abbreviations:** **MR,** Mendelian randomization; **AF**, atrial fibrillation; **SNP**, single-nucleotide polymorphism; **EA**, effect allele; **OA**, other allele; **EAF**, effect allele frequency; **SE**, standard error.

**Table S3-4: Characteristics of instrumental variables for phosphate and EH in the MR analysis**

| **SNP** | **EA** | **OA** | **Exposure** | | | | **EH** | | | |
| --- | --- | --- | --- | --- | --- | --- | --- | --- | --- | --- |
|  |  |  | **EAF** | **Beta** | **SE** | **pval** | **EAF** | **Beta** | **SE** | **pval** |
| rs10051765 | C | T | 0.3324 | -0.0082 | 0.0004 | 3.03E-87 | 0.4006 | 0.0173 | 0.0100 | 0.0849 |
| rs10279171 | T | A | 0.7941 | 0.0029 | 0.0005 | 2.04E-09 | 0.8679 | 0.0295 | 0.0145 | 0.0422 |
| rs10282449 | A | T | 0.1663 | 0.0037 | 0.0005 | 2.15E-12 | 0.1303 | -0.0089 | 0.0147 | 0.5467 |
| rs10401230 | G | A | 0.4937 | -0.0022 | 0.0004 | 9.47E-09 | 0.4658 | 0.0066 | 0.0099 | 0.5028 |
| rs10743976 | C | A | 0.8512 | 0.0067 | 0.0005 | 7.00E-34 | 0.8819 | 0.0107 | 0.0152 | 0.4828 |
| rs11144134 | C | T | 0.0845 | 0.0043 | 0.0007 | 7.16E-10 | 0.0636 | 0.0635 | 0.0203 | 0.0017 |
| rs111723017 | C | G | 0.0875 | 0.0053 | 0.0007 | 1.89E-14 | 0.1204 | 0.0062 | 0.0151 | 0.6838 |
| rs113487566 | T | C | 0.0254 | -0.0102 | 0.0013 | 5.87E-16 | 0.0590 | -0.0088 | 0.0210 | 0.6759 |
| rs11684211 | A | G | 0.3533 | 0.0026 | 0.0004 | 3.81E-10 | 0.2691 | -0.0099 | 0.0111 | 0.3706 |
| rs11801716 | T | C | 0.0462 | -0.0054 | 0.0009 | 9.47E-09 | 0.0849 | 0.0234 | 0.0177 | 0.1869 |
| rs12044944 | T | C | 0.1925 | 0.0029 | 0.0005 | 4.29E-09 | 0.1394 | -0.0121 | 0.0143 | 0.3953 |
| rs12059422 | C | T | 0.0230 | 0.0146 | 0.0013 | 3.32E-29 | 0.0337 | -0.0068 | 0.0273 | 0.8033 |
| rs12132412 | G | A | 0.3884 | 0.0174 | 0.0004 | 1.00E-200 | 0.4044 | 0.0062 | 0.0100 | 0.5372 |
| rs12409158 | C | G | 0.0319 | -0.0064 | 0.0011 | 1.70E-08 | 0.0147 | -0.0292 | 0.0410 | 0.4763 |
| rs12518871 | T | C | 0.4095 | -0.0025 | 0.0004 | 2.57E-10 | 0.4464 | 0.0047 | 0.0099 | 0.6333 |
| rs12710029 | C | T | 0.7762 | -0.0029 | 0.0005 | 7.06E-10 | 0.7286 | -0.0071 | 0.0112 | 0.5272 |
| rs12727188 | G | T | 0.0894 | -0.0054 | 0.0007 | 6.82E-15 | 0.1207 | -0.0016 | 0.0150 | 0.9140 |
| rs12770024 | G | C | 0.1936 | 0.0037 | 0.0005 | 4.92E-14 | 0.1716 | 0.0115 | 0.0130 | 0.3769 |
| rs1286070 | C | T | 0.1721 | -0.0041 | 0.0005 | 2.43E-15 | 0.1541 | 0.0091 | 0.0136 | 0.5046 |
| rs12941512 | T | C | 0.4422 | -0.0032 | 0.0004 | 2.07E-16 | 0.5190 | 0.0050 | 0.0099 | 0.6141 |
| rs12985272 | A | G | 0.0494 | 0.0064 | 0.0009 | 4.69E-12 | 0.0365 | -0.0591 | 0.0267 | 0.0267 |
| rs13232861 | G | A | 0.8125 | 0.0031 | 0.0005 | 3.76E-10 | 0.8446 | 0.0402 | 0.0136 | 0.0031 |
| rs13273161 | T | A | 0.3177 | 0.0030 | 0.0004 | 9.94E-13 | 0.3594 | -0.0119 | 0.0112 | 0.2859 |
| rs138278885 | CA | C | 0.1007 | -0.0057 | 0.0007 | 3.68E-18 | 0.0883 | -0.0282 | 0.0173 | 0.1028 |
| rs139796809 | T | C | 0.5570 | -0.0028 | 0.0004 | 1.53E-11 | 0.5510 | 0.0204 | 0.0101 | 0.0435 |
| rs144214505 | T | C | 0.0416 | -0.0057 | 0.0010 | 7.05E-09 | 0.0366 | 0.0063 | 0.0264 | 0.8120 |
| rs145330264 | C | A | 0.0054 | -0.0321 | 0.0028 | 5.75E-30 | 0.0009 | -0.0875 | 0.1681 | 0.6028 |
| rs145978363 | G | A | 0.0301 | -0.0072 | 0.0012 | 1.89E-09 | 0.0178 | -0.0030 | 0.0376 | 0.9367 |
| rs1468908 | G | A | 0.4920 | 0.0050 | 0.0004 | 6.28E-37 | 0.5382 | -0.0050 | 0.0099 | 0.6099 |
| rs1483842 | C | T | 0.4808 | -0.0028 | 0.0004 | 1.42E-12 | 0.4857 | 0.0227 | 0.0098 | 0.0208 |
| rs1570603 | A | G | 0.5720 | -0.0022 | 0.0004 | 3.69E-08 | 0.5824 | -0.0225 | 0.0099 | 0.0234 |
| rs17036160 | T | C | 0.1180 | -0.0040 | 0.0006 | 5.67E-11 | 0.1714 | -0.0269 | 0.0130 | 0.0389 |
| rs17288460 | A | G | 0.1803 | -0.0033 | 0.0005 | 7.84E-11 | 0.1764 | -0.0059 | 0.0129 | 0.6454 |
| rs17884869 | A | G | 0.0250 | 0.0079 | 0.0013 | 2.58E-10 | 0.0361 | -0.0203 | 0.0264 | 0.4423 |
| rs1889208 | A | G | 0.3359 | 0.0033 | 0.0004 | 3.76E-15 | 0.3855 | -0.0051 | 0.0101 | 0.6105 |
| rs209961 | C | T | 0.7219 | 0.0037 | 0.0004 | 1.38E-17 | 0.7848 | 0.0144 | 0.0120 | 0.2278 |
| rs2177470 | A | T | 0.6819 | -0.0027 | 0.0004 | 2.60E-10 | 0.7253 | 0.0115 | 0.0111 | 0.2979 |
| rs2420055 | C | T | 0.8426 | -0.0035 | 0.0005 | 4.21E-11 | 0.8674 | -0.0068 | 0.0145 | 0.6384 |
| rs273587 | T | A | 0.6778 | -0.0030 | 0.0004 | 7.53E-13 | 0.6247 | -0.0338 | 0.0101 | 0.0008 |
| rs28542318 | T | A | 0.8344 | 0.0032 | 0.0005 | 1.75E-09 | 0.7492 | -0.0088 | 0.0119 | 0.4587 |
| rs2909212 | G | A | 0.2966 | 0.0024 | 0.0004 | 2.31E-08 | 0.2808 | 0.0223 | 0.0109 | 0.0413 |
| rs2959652 | T | G | 0.9080 | -0.0037 | 0.0007 | 3.13E-08 | 0.8945 | 0.0048 | 0.0160 | 0.7647 |
| rs296847 | T | G | 0.6643 | 0.0024 | 0.0004 | 8.87E-09 | 0.6396 | 0.0176 | 0.0102 | 0.0853 |
| rs2970818 | A | T | 0.1038 | 0.0206 | 0.0006 | 1.00E-200 | 0.0622 | 0.0248 | 0.0203 | 0.2202 |
| rs2982572 | T | C | 0.4208 | -0.0041 | 0.0004 | 1.83E-25 | 0.4624 | 0.0139 | 0.0098 | 0.1559 |
| rs308032 | T | G | 0.2368 | -0.0029 | 0.0005 | 1.03E-09 | 0.1671 | -0.0331 | 0.0132 | 0.0124 |
| rs3091842 | A | G | 0.0441 | -0.0086 | 0.0010 | 1.49E-18 | 0.0884 | 0.0043 | 0.0174 | 0.8028 |
| rs339969 | A | C | 0.6148 | 0.0027 | 0.0004 | 2.35E-11 | 0.7195 | -0.0044 | 0.0109 | 0.6886 |
| rs35026779 | T | C | 0.0997 | -0.0042 | 0.0007 | 9.54E-11 | 0.0981 | 0.0026 | 0.0166 | 0.8742 |
| rs35666081 | A | G | 0.4002 | 0.0022 | 0.0004 | 4.52E-08 | 0.4776 | 0.0150 | 0.0098 | 0.1262 |
| rs3813498 | T | C | 0.8128 | 0.0051 | 0.0005 | 3.42E-24 | 0.7761 | 0.0148 | 0.0118 | 0.2096 |
| rs4072537 | C | T | 0.6811 | -0.0023 | 0.0004 | 3.95E-08 | 0.6386 | 0.0042 | 0.0103 | 0.6813 |
| rs4077450 | T | G | 0.8280 | -0.0042 | 0.0005 | 1.15E-15 | 0.7980 | -0.0160 | 0.0122 | 0.1900 |
| rs41278174 | A | G | 0.0273 | 0.0170 | 0.0012 | 1.31E-45 | 0.0332 | 0.0121 | 0.0272 | 0.6560 |
| rs4509682 | A | G | 0.3919 | -0.0031 | 0.0004 | 5.48E-15 | 0.3745 | -0.0035 | 0.0101 | 0.7271 |
| rs453639 | A | C | 0.6405 | -0.0107 | 0.0004 | 2.10E-150 | 0.6661 | 0.0205 | 0.0106 | 0.0526 |
| rs4706554 | A | G | 0.4763 | 0.0029 | 0.0004 | 2.22E-13 | 0.4130 | -0.0029 | 0.0099 | 0.7738 |
| rs4759844 | G | A | 0.3509 | 0.0038 | 0.0004 | 1.22E-20 | 0.2865 | 0.0246 | 0.0109 | 0.0232 |
| rs4795607 | T | C | 0.6140 | 0.0024 | 0.0004 | 1.58E-09 | 0.5965 | -0.0180 | 0.0100 | 0.0726 |
| rs4820324 | C | G | 0.5810 | 0.0030 | 0.0004 | 3.78E-14 | 0.5820 | 0.0160 | 0.0099 | 0.1070 |
| rs4869745 | T | C | 0.2871 | 0.0031 | 0.0004 | 6.73E-13 | 0.2945 | 0.0189 | 0.0108 | 0.0805 |
| rs4938642 | C | G | 0.0740 | 0.0041 | 0.0007 | 4.74E-08 | 0.0274 | 0.0569 | 0.0303 | 0.0600 |
| rs4982711 | G | T | 0.5710 | 0.0023 | 0.0004 | 1.05E-08 | 0.5677 | -0.0230 | 0.0099 | 0.0202 |
| rs5435 | C | T | 0.6121 | -0.0022 | 0.0004 | 4.08E-08 | 0.6211 | 0.0263 | 0.0101 | 0.0096 |
| rs55923451 | A | G | 0.1791 | -0.0034 | 0.0005 | 5.39E-11 | 0.1409 | -0.0288 | 0.0141 | 0.0412 |
| rs56078331 | T | C | 0.0544 | 0.0084 | 0.0009 | 2.07E-22 | 0.0410 | 0.0360 | 0.0248 | 0.1459 |
| rs56328339 | T | C | 0.1628 | 0.0052 | 0.0005 | 1.05E-22 | 0.2083 | -0.0031 | 0.0121 | 0.7961 |
| rs596940 | C | T | 0.0716 | 0.0052 | 0.0008 | 1.16E-11 | 0.0756 | -0.0136 | 0.0186 | 0.4638 |
| rs60447213 | T | G | 0.0699 | -0.0299 | 0.0008 | 1.00E-200 | 0.1088 | -0.0102 | 0.0160 | 0.5217 |
| rs6136492 | T | C | 0.2259 | -0.0027 | 0.0005 | 5.88E-09 | 0.1898 | -0.0156 | 0.0125 | 0.2138 |
| rs61870274 | A | C | 0.0781 | 0.0059 | 0.0007 | 7.92E-16 | 0.0811 | 0.0154 | 0.0179 | 0.3895 |
| rs61909254 | C | T | 0.8598 | 0.0101 | 0.0006 | 8.49E-72 | 0.8420 | 0.0292 | 0.0136 | 0.0315 |
| rs62289280 | C | T | 0.3053 | -0.0029 | 0.0004 | 1.09E-11 | 0.2725 | -0.0118 | 0.0110 | 0.2827 |
| rs62383568 | C | T | 0.3497 | -0.0024 | 0.0004 | 3.35E-09 | 0.3249 | -0.0087 | 0.0105 | 0.4065 |
| rs6751217 | T | C | 0.6316 | 0.0029 | 0.0004 | 6.03E-13 | 0.6198 | -0.0084 | 0.0101 | 0.4067 |
| rs6780593 | A | G | 0.1528 | -0.0045 | 0.0005 | 8.55E-17 | 0.1766 | -0.0134 | 0.0129 | 0.2984 |
| rs6841258 | T | C | 0.1663 | 0.0031 | 0.0005 | 5.29E-09 | 0.1307 | -0.0053 | 0.0146 | 0.7172 |
| rs6911602 | A | G | 0.1318 | 0.0082 | 0.0006 | 5.07E-46 | 0.1112 | -0.0277 | 0.0156 | 0.0762 |
| rs6983239 | T | G | 0.2186 | 0.0039 | 0.0005 | 3.52E-16 | 0.1871 | 0.0001 | 0.0126 | 0.9947 |
| rs7017252 | T | C | 0.3830 | 0.0022 | 0.0004 | 3.53E-08 | 0.2948 | -0.0083 | 0.0108 | 0.4395 |
| rs7033278 | T | C | 0.2930 | 0.0035 | 0.0004 | 5.96E-16 | 0.3152 | -0.0067 | 0.0106 | 0.5288 |
| rs706300 | G | A | 0.8575 | -0.0054 | 0.0006 | 2.96E-22 | 0.8816 | 0.0028 | 0.0152 | 0.8541 |
| rs71368113 | G | C | 0.0709 | -0.0045 | 0.0008 | 4.44E-08 | 0.0578 | 0.0206 | 0.0212 | 0.3312 |
| rs71413981 | A | G | 0.1637 | -0.0031 | 0.0005 | 6.93E-09 | 0.1374 | 0.0107 | 0.0143 | 0.4564 |
| rs72660359 | T | G | 0.0373 | 0.0148 | 0.0010 | 1.30E-45 | 0.0355 | 0.0279 | 0.0268 | 0.2976 |
| rs72716022 | T | C | 0.2021 | -0.0033 | 0.0005 | 1.09E-10 | 0.1877 | -0.0136 | 0.0126 | 0.2824 |
| rs72974112 | T | C | 0.2373 | -0.0029 | 0.0005 | 4.78E-10 | 0.1812 | 0.0109 | 0.0129 | 0.3972 |
| rs7324259 | T | G | 0.1259 | 0.0037 | 0.0006 | 4.73E-10 | 0.1637 | 0.0224 | 0.0135 | 0.0965 |
| rs7529925 | T | C | 0.7383 | 0.0031 | 0.0004 | 7.01E-12 | 0.7227 | -0.0004 | 0.0110 | 0.9742 |
| rs7555660 | T | C | 0.0853 | 0.0090 | 0.0007 | 4.91E-38 | 0.0730 | 0.0046 | 0.0191 | 0.8088 |
| rs7625643 | G | A | 0.4482 | 0.0026 | 0.0004 | 7.39E-11 | 0.4593 | 0.0124 | 0.0099 | 0.2096 |
| rs7672820 | C | G | 0.1863 | 0.0043 | 0.0005 | 6.26E-18 | 0.1881 | 0.0048 | 0.0126 | 0.7031 |
| rs76819459 | G | A | 0.3270 | -0.0051 | 0.0004 | 1.15E-34 | 0.3092 | -0.0161 | 0.0107 | 0.1317 |
| rs77149268 | T | C | 0.1051 | 0.0066 | 0.0006 | 5.82E-25 | 0.0670 | -0.0074 | 0.0197 | 0.7061 |
| rs7726961 | T | C | 0.4261 | -0.0023 | 0.0004 | 6.85E-09 | 0.4069 | 0.0011 | 0.0100 | 0.9125 |
| rs77282753 | C | T | 0.0480 | -0.0069 | 0.0009 | 4.06E-14 | 0.0530 | -0.0035 | 0.0220 | 0.8728 |
| rs7860558 | C | T | 0.7013 | -0.0029 | 0.0004 | 3.13E-11 | 0.7083 | -0.0034 | 0.0109 | 0.7516 |
| rs79472736 | A | G | 0.0354 | -0.0070 | 0.0011 | 3.45E-11 | 0.0335 | -0.0396 | 0.0273 | 0.1468 |
| rs805288 | T | C | 0.2491 | 0.0025 | 0.0005 | 4.98E-08 | 0.3839 | -0.0229 | 0.0112 | 0.0419 |
| rs8075449 | T | C | 0.2590 | -0.0028 | 0.0004 | 5.03E-10 | 0.3386 | -0.0085 | 0.0104 | 0.4151 |
| rs838717 | A | G | 0.5656 | 0.0049 | 0.0004 | 3.04E-35 | 0.5845 | -0.0128 | 0.0100 | 0.2001 |
| rs872386 | A | G | 0.5310 | -0.0039 | 0.0004 | 2.60E-23 | 0.5866 | -0.0010 | 0.0100 | 0.9183 |
| rs896788 | T | C | 0.1552 | 0.0042 | 0.0005 | 6.52E-15 | 0.2142 | 0.0134 | 0.0120 | 0.2612 |
| rs912981 | C | A | 0.5810 | -0.0026 | 0.0004 | 3.22E-11 | 0.6544 | 0.0072 | 0.0103 | 0.4877 |
| rs9574586 | C | T | 0.3311 | -0.0023 | 0.0004 | 2.35E-08 | 0.3287 | 0.0085 | 0.0104 | 0.4131 |
| rs9689096 | C | A | 0.0639 | -0.0070 | 0.0008 | 4.31E-18 | 0.0322 | 0.0807 | 0.0280 | 0.0039 |
| rs9745989 | C | T | 0.6281 | 0.0025 | 0.0004 | 9.31E-10 | 0.6886 | 0.0384 | 0.0107 | 0.0003 |

The F-statistics for phosphate was 34.23. **Abbreviations:** **MR,** Mendelian randomization; **EH**, essential hypertension; **SNP**, single-nucleotide polymorphism; **EA**, effect allele; **OA**, other allele; **EAF**, effect allele frequency; **SE**, standard error.

**Table S3-5: Characteristics of instrumental variables for phosphate and VHD in the MR analysis**

| **SNP** | **EA** | **OA** | **Exposure** | | | | **VHD** | | | |
| --- | --- | --- | --- | --- | --- | --- | --- | --- | --- | --- |
|  |  |  | **EAF** | **Beta** | **SE** | **pval** | **EAF** | **Beta** | **SE** | **pval** |
| rs10051765 | C | T | 0.3324 | -0.0082 | 0.0004 | 3.03E-87 | 0.4005 | -0.0093 | 0.0093 | 0.3202 |
| rs10279171 | T | A | 0.7941 | 0.0029 | 0.0005 | 2.04E-09 | 0.8678 | -0.0224 | 0.0135 | 0.0966 |
| rs10282449 | A | T | 0.1663 | 0.0037 | 0.0005 | 2.15E-12 | 0.1306 | 0.0122 | 0.0136 | 0.3704 |
| rs10401230 | G | A | 0.4937 | -0.0022 | 0.0004 | 9.47E-09 | 0.4660 | -0.0002 | 0.0092 | 0.9809 |
| rs10743976 | C | A | 0.8512 | 0.0067 | 0.0005 | 7.00E-34 | 0.8820 | -0.0026 | 0.0142 | 0.8544 |
| rs11144134 | C | T | 0.0845 | 0.0043 | 0.0007 | 7.16E-10 | 0.0636 | 0.0026 | 0.0188 | 0.8898 |
| rs111723017 | C | G | 0.0875 | 0.0053 | 0.0007 | 1.89E-14 | 0.1200 | 0.0060 | 0.0141 | 0.6689 |
| rs113487566 | T | C | 0.0254 | -0.0102 | 0.0013 | 5.87E-16 | 0.0590 | -0.0246 | 0.0196 | 0.2096 |
| rs11684211 | A | G | 0.3533 | 0.0026 | 0.0004 | 3.81E-10 | 0.2692 | -0.0035 | 0.0103 | 0.7323 |
| rs11801716 | T | C | 0.0462 | -0.0054 | 0.0009 | 9.47E-09 | 0.0850 | 0.0099 | 0.0165 | 0.5492 |
| rs12044944 | T | C | 0.1925 | 0.0029 | 0.0005 | 4.29E-09 | 0.1396 | 0.0009 | 0.0132 | 0.9470 |
| rs12059422 | C | T | 0.0230 | 0.0146 | 0.0013 | 3.32E-29 | 0.0337 | 0.0207 | 0.0254 | 0.4151 |
| rs12132412 | G | A | 0.3884 | 0.0174 | 0.0004 | 1.00E-200 | 0.4041 | 0.0291 | 0.0093 | 0.0018 |
| rs12409158 | C | G | 0.0319 | -0.0064 | 0.0011 | 1.70E-08 | 0.0146 | -0.0160 | 0.0381 | 0.6740 |
| rs12518871 | T | C | 0.4095 | -0.0025 | 0.0004 | 2.57E-10 | 0.4465 | 0.0087 | 0.0092 | 0.3464 |
| rs12710029 | C | T | 0.7762 | -0.0029 | 0.0005 | 7.06E-10 | 0.7282 | -0.0046 | 0.0104 | 0.6567 |
| rs12727188 | G | T | 0.0894 | -0.0054 | 0.0007 | 6.82E-15 | 0.1204 | 0.0091 | 0.0140 | 0.5147 |
| rs12770024 | G | C | 0.1936 | 0.0037 | 0.0005 | 4.92E-14 | 0.1713 | -0.0298 | 0.0121 | 0.0139 |
| rs1286070 | C | T | 0.1721 | -0.0041 | 0.0005 | 2.43E-15 | 0.1540 | -0.0097 | 0.0127 | 0.4445 |
| rs12941512 | T | C | 0.4422 | -0.0032 | 0.0004 | 2.07E-16 | 0.5192 | 0.0083 | 0.0092 | 0.3696 |
| rs12985272 | A | G | 0.0494 | 0.0064 | 0.0009 | 4.69E-12 | 0.0365 | 0.0053 | 0.0247 | 0.8294 |
| rs13232861 | G | A | 0.8125 | 0.0031 | 0.0005 | 3.76E-10 | 0.8444 | 0.0164 | 0.0126 | 0.1941 |
| rs13273161 | T | A | 0.3177 | 0.0030 | 0.0004 | 9.94E-13 | 0.3597 | -0.0219 | 0.0104 | 0.0364 |
| rs138278885 | CA | C | 0.1007 | -0.0057 | 0.0007 | 3.68E-18 | 0.0882 | -0.0517 | 0.0161 | 0.0013 |
| rs139796809 | T | C | 0.5570 | -0.0028 | 0.0004 | 1.53E-11 | 0.5509 | -0.0020 | 0.0094 | 0.8317 |
| rs144214505 | T | C | 0.0416 | -0.0057 | 0.0010 | 7.05E-09 | 0.0363 | -0.0030 | 0.0247 | 0.9027 |
| rs145330264 | C | A | 0.0054 | -0.0321 | 0.0028 | 5.75E-30 | 0.0009 | -0.1517 | 0.1553 | 0.3286 |
| rs145978363 | G | A | 0.0301 | -0.0072 | 0.0012 | 1.89E-09 | 0.0177 | 0.0070 | 0.0350 | 0.8413 |
| rs1468908 | G | A | 0.4920 | 0.0050 | 0.0004 | 6.28E-37 | 0.5384 | 0.0048 | 0.0092 | 0.6025 |
| rs1483842 | C | T | 0.4808 | -0.0028 | 0.0004 | 1.42E-12 | 0.4858 | 0.0025 | 0.0091 | 0.7812 |
| rs1570603 | A | G | 0.5720 | -0.0022 | 0.0004 | 3.69E-08 | 0.5824 | 0.0081 | 0.0092 | 0.3794 |
| rs17036160 | T | C | 0.1180 | -0.0040 | 0.0006 | 5.67E-11 | 0.1708 | -0.0281 | 0.0121 | 0.0202 |
| rs17288460 | A | G | 0.1803 | -0.0033 | 0.0005 | 7.84E-11 | 0.1764 | 0.0092 | 0.0120 | 0.4445 |
| rs17884869 | A | G | 0.0250 | 0.0079 | 0.0013 | 2.58E-10 | 0.0361 | -0.0046 | 0.0245 | 0.8513 |
| rs1889208 | A | G | 0.3359 | 0.0033 | 0.0004 | 3.76E-15 | 0.3856 | 0.0041 | 0.0094 | 0.6594 |
| rs209961 | C | T | 0.7219 | 0.0037 | 0.0004 | 1.38E-17 | 0.7847 | 0.0088 | 0.0111 | 0.4269 |
| rs2177470 | A | T | 0.6819 | -0.0027 | 0.0004 | 2.60E-10 | 0.7256 | 0.0091 | 0.0103 | 0.3776 |
| rs2420055 | C | T | 0.8426 | -0.0035 | 0.0005 | 4.21E-11 | 0.8672 | -0.0033 | 0.0134 | 0.8081 |
| rs273587 | T | A | 0.6778 | -0.0030 | 0.0004 | 7.53E-13 | 0.6247 | -0.0139 | 0.0094 | 0.1395 |
| rs28542318 | T | A | 0.8344 | 0.0032 | 0.0005 | 1.75E-09 | 0.7487 | 0.0112 | 0.0111 | 0.3140 |
| rs2909212 | G | A | 0.2966 | 0.0024 | 0.0004 | 2.31E-08 | 0.2806 | 0.0132 | 0.0102 | 0.1956 |
| rs2959652 | T | G | 0.9080 | -0.0037 | 0.0007 | 3.13E-08 | 0.8945 | 0.0118 | 0.0149 | 0.4276 |
| rs296847 | T | G | 0.6643 | 0.0024 | 0.0004 | 8.87E-09 | 0.6396 | 0.0017 | 0.0095 | 0.8621 |
| rs2970818 | A | T | 0.1038 | 0.0206 | 0.0006 | 1.00E-200 | 0.0624 | 0.0238 | 0.0188 | 0.2062 |
| rs2982572 | T | C | 0.4208 | -0.0041 | 0.0004 | 1.83E-25 | 0.4623 | -0.0004 | 0.0091 | 0.9654 |
| rs308032 | T | G | 0.2368 | -0.0029 | 0.0005 | 1.03E-09 | 0.1667 | -0.0228 | 0.0123 | 0.0638 |
| rs3091842 | A | G | 0.0441 | -0.0086 | 0.0010 | 1.49E-18 | 0.0886 | -0.0088 | 0.0161 | 0.5849 |
| rs339969 | A | C | 0.6148 | 0.0027 | 0.0004 | 2.35E-11 | 0.7195 | -0.0009 | 0.0102 | 0.9296 |
| rs35026779 | T | C | 0.0997 | -0.0042 | 0.0007 | 9.54E-11 | 0.0981 | -0.0050 | 0.0154 | 0.7471 |
| rs35666081 | A | G | 0.4002 | 0.0022 | 0.0004 | 4.52E-08 | 0.4774 | -0.0002 | 0.0091 | 0.9843 |
| rs3813498 | T | C | 0.8128 | 0.0051 | 0.0005 | 3.42E-24 | 0.7761 | 0.0170 | 0.0110 | 0.1217 |
| rs4072537 | C | T | 0.6811 | -0.0023 | 0.0004 | 3.95E-08 | 0.6389 | 0.0131 | 0.0096 | 0.1727 |
| rs4077450 | T | G | 0.8280 | -0.0042 | 0.0005 | 1.15E-15 | 0.7979 | -0.0153 | 0.0114 | 0.1790 |
| rs41278174 | A | G | 0.0273 | 0.0170 | 0.0012 | 1.31E-45 | 0.0332 | 0.0105 | 0.0254 | 0.6802 |
| rs4509682 | A | G | 0.3919 | -0.0031 | 0.0004 | 5.48E-15 | 0.3748 | -0.0204 | 0.0094 | 0.0305 |
| rs453639 | A | C | 0.6405 | -0.0107 | 0.0004 | 2.10E-150 | 0.6657 | 0.0045 | 0.0098 | 0.6462 |
| rs4706554 | A | G | 0.4763 | 0.0029 | 0.0004 | 2.22E-13 | 0.4133 | -0.0108 | 0.0092 | 0.2437 |
| rs4759844 | G | A | 0.3509 | 0.0038 | 0.0004 | 1.22E-20 | 0.2861 | 0.0061 | 0.0101 | 0.5485 |
| rs4795607 | T | C | 0.6140 | 0.0024 | 0.0004 | 1.58E-09 | 0.5964 | 0.0158 | 0.0093 | 0.0895 |
| rs4820324 | C | G | 0.5810 | 0.0030 | 0.0004 | 3.78E-14 | 0.5820 | 0.0040 | 0.0092 | 0.6676 |
| rs4869745 | T | C | 0.2871 | 0.0031 | 0.0004 | 6.73E-13 | 0.2946 | 0.0149 | 0.0101 | 0.1373 |
| rs4938642 | C | G | 0.0740 | 0.0041 | 0.0007 | 4.74E-08 | 0.0274 | 0.0298 | 0.0281 | 0.2887 |
| rs4982711 | G | T | 0.5710 | 0.0023 | 0.0004 | 1.05E-08 | 0.5681 | 0.0115 | 0.0092 | 0.2102 |
| rs5435 | C | T | 0.6121 | -0.0022 | 0.0004 | 4.08E-08 | 0.6205 | 0.0017 | 0.0094 | 0.8598 |
| rs55923451 | A | G | 0.1791 | -0.0034 | 0.0005 | 5.39E-11 | 0.1407 | -0.0151 | 0.0131 | 0.2498 |
| rs56078331 | T | C | 0.0544 | 0.0084 | 0.0009 | 2.07E-22 | 0.0411 | 0.0180 | 0.0229 | 0.4313 |
| rs56328339 | T | C | 0.1628 | 0.0052 | 0.0005 | 1.05E-22 | 0.2086 | 0.0146 | 0.0112 | 0.1936 |
| rs596940 | C | T | 0.0716 | 0.0052 | 0.0008 | 1.16E-11 | 0.0756 | -0.0050 | 0.0173 | 0.7706 |
| rs60447213 | T | G | 0.0699 | -0.0299 | 0.0008 | 1.00E-200 | 0.1086 | -0.0002 | 0.0148 | 0.9911 |
| rs6136492 | T | C | 0.2259 | -0.0027 | 0.0005 | 5.88E-09 | 0.1898 | -0.0103 | 0.0116 | 0.3764 |
| rs61870274 | A | C | 0.0781 | 0.0059 | 0.0007 | 7.92E-16 | 0.0813 | -0.0018 | 0.0167 | 0.9124 |
| rs61909254 | C | T | 0.8598 | 0.0101 | 0.0006 | 8.49E-72 | 0.8422 | 0.0290 | 0.0126 | 0.0220 |
| rs62289280 | C | T | 0.3053 | -0.0029 | 0.0004 | 1.09E-11 | 0.2725 | -0.0003 | 0.0102 | 0.9795 |
| rs62383568 | C | T | 0.3497 | -0.0024 | 0.0004 | 3.35E-09 | 0.3253 | -0.0119 | 0.0097 | 0.2228 |
| rs6751217 | T | C | 0.6316 | 0.0029 | 0.0004 | 6.03E-13 | 0.6196 | -0.0047 | 0.0094 | 0.6167 |
| rs6780593 | A | G | 0.1528 | -0.0045 | 0.0005 | 8.55E-17 | 0.1770 | -0.0239 | 0.0119 | 0.0451 |
| rs6841258 | T | C | 0.1663 | 0.0031 | 0.0005 | 5.29E-09 | 0.1302 | -0.0140 | 0.0136 | 0.3016 |
| rs6911602 | A | G | 0.1318 | 0.0082 | 0.0006 | 5.07E-46 | 0.1114 | 0.0026 | 0.0145 | 0.8597 |
| rs6983239 | T | G | 0.2186 | 0.0039 | 0.0005 | 3.52E-16 | 0.1872 | 0.0240 | 0.0117 | 0.0408 |
| rs7017252 | T | C | 0.3830 | 0.0022 | 0.0004 | 3.53E-08 | 0.2948 | 0.0074 | 0.0100 | 0.4582 |
| rs7033278 | T | C | 0.2930 | 0.0035 | 0.0004 | 5.96E-16 | 0.3158 | -0.0191 | 0.0099 | 0.0525 |
| rs706300 | G | A | 0.8575 | -0.0054 | 0.0006 | 2.96E-22 | 0.8816 | 0.0206 | 0.0141 | 0.1438 |
| rs71368113 | G | C | 0.0709 | -0.0045 | 0.0008 | 4.44E-08 | 0.0579 | -0.0021 | 0.0196 | 0.9161 |
| rs71413981 | A | G | 0.1637 | -0.0031 | 0.0005 | 6.93E-09 | 0.1376 | -0.0129 | 0.0133 | 0.3347 |
| rs72660359 | T | G | 0.0373 | 0.0148 | 0.0010 | 1.30E-45 | 0.0357 | 0.0497 | 0.0247 | 0.0446 |
| rs72716022 | T | C | 0.2021 | -0.0033 | 0.0005 | 1.09E-10 | 0.1882 | -0.0029 | 0.0117 | 0.8069 |
| rs72974112 | T | C | 0.2373 | -0.0029 | 0.0005 | 4.78E-10 | 0.1812 | 0.0128 | 0.0119 | 0.2845 |
| rs7324259 | T | G | 0.1259 | 0.0037 | 0.0006 | 4.73E-10 | 0.1635 | -0.0030 | 0.0126 | 0.8092 |
| rs7529925 | T | C | 0.7383 | 0.0031 | 0.0004 | 7.01E-12 | 0.7224 | 0.0069 | 0.0102 | 0.5003 |
| rs7555660 | T | C | 0.0853 | 0.0090 | 0.0007 | 4.91E-38 | 0.0733 | 0.0305 | 0.0177 | 0.0844 |
| rs7625643 | G | A | 0.4482 | 0.0026 | 0.0004 | 7.39E-11 | 0.4593 | 0.0038 | 0.0092 | 0.6804 |
| rs7672820 | C | G | 0.1863 | 0.0043 | 0.0005 | 6.26E-18 | 0.1878 | 0.0117 | 0.0117 | 0.3206 |
| rs76819459 | G | A | 0.3270 | -0.0051 | 0.0004 | 1.15E-34 | 0.3092 | -0.0219 | 0.0099 | 0.0273 |
| rs77149268 | T | C | 0.1051 | 0.0066 | 0.0006 | 5.82E-25 | 0.0670 | 0.0169 | 0.0183 | 0.3544 |
| rs7726961 | T | C | 0.4261 | -0.0023 | 0.0004 | 6.85E-09 | 0.4069 | -0.0099 | 0.0093 | 0.2875 |
| rs77282753 | C | T | 0.0480 | -0.0069 | 0.0009 | 4.06E-14 | 0.0530 | 0.0323 | 0.0205 | 0.1148 |
| rs7860558 | C | T | 0.7013 | -0.0029 | 0.0004 | 3.13E-11 | 0.7075 | -0.0214 | 0.0101 | 0.0343 |
| rs79472736 | A | G | 0.0354 | -0.0070 | 0.0011 | 3.45E-11 | 0.0335 | 0.0068 | 0.0252 | 0.7873 |
| rs805288 | T | C | 0.2491 | 0.0025 | 0.0005 | 4.98E-08 | 0.3851 | -0.0119 | 0.0104 | 0.2546 |
| rs8075449 | T | C | 0.2590 | -0.0028 | 0.0004 | 5.03E-10 | 0.3387 | 0.0021 | 0.0097 | 0.8310 |
| rs838717 | A | G | 0.5656 | 0.0049 | 0.0004 | 3.04E-35 | 0.5842 | -0.0217 | 0.0093 | 0.0192 |
| rs872386 | A | G | 0.5310 | -0.0039 | 0.0004 | 2.60E-23 | 0.5867 | -0.0065 | 0.0093 | 0.4817 |
| rs896788 | T | C | 0.1552 | 0.0042 | 0.0005 | 6.52E-15 | 0.2141 | 0.0072 | 0.0111 | 0.5196 |
| rs912981 | C | A | 0.5810 | -0.0026 | 0.0004 | 3.22E-11 | 0.6542 | 0.0145 | 0.0096 | 0.1319 |
| rs9574586 | C | T | 0.3311 | -0.0023 | 0.0004 | 2.35E-08 | 0.3282 | 0.0068 | 0.0097 | 0.4841 |
| rs9689096 | C | A | 0.0639 | -0.0070 | 0.0008 | 4.31E-18 | 0.0322 | 0.0758 | 0.0260 | 0.0035 |
| rs9745989 | C | T | 0.6281 | 0.0025 | 0.0004 | 9.31E-10 | 0.6891 | 0.0217 | 0.0100 | 0.0292 |

The F-statistics for phosphate was 34.23. **Abbreviations:** **MR,** Mendelian randomization; **VHD**, valve heart diseases; **SNP**, single-nucleotide polymorphism; **EA**, effect allele; **OA**, other allele; **EAF**, effect allele frequency; **SE**, standard error.

**Table S3-6: Characteristics of instrumental variables for phosphate and non-rheumatic valve diseases in the MR analysis**

| **SNP** | **EA** | **OA** | **Exposure** | | | | **Non-rheumatic valve diseases** | | | |
| --- | --- | --- | --- | --- | --- | --- | --- | --- | --- | --- |
|  |  |  | **EAF** | **Beta** | **SE** | **pval** | **EAF** | **Beta** | **SE** | **pval** |
| rs10051765 | C | T | 0.3324 | -0.0082 | 0.0004 | 3.03E-87 | 0.4007 | -0.0092 | 0.0163 | 0.5731 |
| rs10279171 | T | A | 0.7941 | 0.0029 | 0.0005 | 2.04E-09 | 0.8679 | -0.0289 | 0.0236 | 0.2209 |
| rs10282449 | A | T | 0.1663 | 0.0037 | 0.0005 | 2.15E-12 | 0.1306 | 0.0217 | 0.0239 | 0.3641 |
| rs10401230 | G | A | 0.4937 | -0.0022 | 0.0004 | 9.47E-09 | 0.4663 | 0.0229 | 0.0161 | 0.1543 |
| rs10743976 | C | A | 0.8512 | 0.0067 | 0.0005 | 7.00E-34 | 0.8823 | 0.0298 | 0.0249 | 0.2303 |
| rs11144134 | C | T | 0.0845 | 0.0043 | 0.0007 | 7.16E-10 | 0.0635 | -0.0295 | 0.0331 | 0.3724 |
| rs111723017 | C | G | 0.0875 | 0.0053 | 0.0007 | 1.89E-14 | 0.1198 | -0.0119 | 0.0246 | 0.6300 |
| rs113487566 | T | C | 0.0254 | -0.0102 | 0.0013 | 5.87E-16 | 0.0592 | -0.0729 | 0.0340 | 0.0323 |
| rs11684211 | A | G | 0.3533 | 0.0026 | 0.0004 | 3.81E-10 | 0.2692 | -0.0002 | 0.0180 | 0.9892 |
| rs11801716 | T | C | 0.0462 | -0.0054 | 0.0009 | 9.47E-09 | 0.0852 | 0.0225 | 0.0288 | 0.4350 |
| rs12044944 | T | C | 0.1925 | 0.0029 | 0.0005 | 4.29E-09 | 0.1400 | 0.0423 | 0.0231 | 0.0667 |
| rs12059422 | C | T | 0.0230 | 0.0146 | 0.0013 | 3.32E-29 | 0.0336 | 0.0118 | 0.0446 | 0.7922 |
| rs12132412 | G | A | 0.3884 | 0.0174 | 0.0004 | 1.00E-200 | 0.4034 | 0.0374 | 0.0163 | 0.0219 |
| rs12409158 | C | G | 0.0319 | -0.0064 | 0.0011 | 1.70E-08 | 0.0146 | -0.0049 | 0.0665 | 0.9416 |
| rs12518871 | T | C | 0.4095 | -0.0025 | 0.0004 | 2.57E-10 | 0.4464 | 0.0109 | 0.0161 | 0.5007 |
| rs12710029 | C | T | 0.7762 | -0.0029 | 0.0005 | 7.06E-10 | 0.7284 | 0.0011 | 0.0181 | 0.9495 |
| rs12727188 | G | T | 0.0894 | -0.0054 | 0.0007 | 6.82E-15 | 0.1201 | -0.0090 | 0.0246 | 0.7155 |
| rs12770024 | G | C | 0.1936 | 0.0037 | 0.0005 | 4.92E-14 | 0.1716 | -0.0420 | 0.0212 | 0.0475 |
| rs1286070 | C | T | 0.1721 | -0.0041 | 0.0005 | 2.43E-15 | 0.1539 | -0.0209 | 0.0222 | 0.3473 |
| rs12941512 | T | C | 0.4422 | -0.0032 | 0.0004 | 2.07E-16 | 0.5191 | -0.0018 | 0.0161 | 0.9135 |
| rs12985272 | A | G | 0.0494 | 0.0064 | 0.0009 | 4.69E-12 | 0.0365 | -0.0045 | 0.0432 | 0.9168 |
| rs13232861 | G | A | 0.8125 | 0.0031 | 0.0005 | 3.76E-10 | 0.8443 | 0.0319 | 0.0221 | 0.1498 |
| rs13273161 | T | A | 0.3177 | 0.0030 | 0.0004 | 9.94E-13 | 0.3604 | -0.0175 | 0.0182 | 0.3356 |
| rs138278885 | CA | C | 0.1007 | -0.0057 | 0.0007 | 3.68E-18 | 0.0882 | -0.1148 | 0.0281 | 0.0000 |
| rs139796809 | T | C | 0.5570 | -0.0028 | 0.0004 | 1.53E-11 | 0.5515 | 0.0296 | 0.0164 | 0.0715 |
| rs144214505 | T | C | 0.0416 | -0.0057 | 0.0010 | 7.05E-09 | 0.0365 | 0.0463 | 0.0425 | 0.2760 |
| rs145330264 | C | A | 0.0054 | -0.0321 | 0.0028 | 5.75E-30 | 0.0221 | 0.0173 | 0.0553 | 0.7549 |
| rs145978363 | G | A | 0.0301 | -0.0072 | 0.0012 | 1.89E-09 | 0.0176 | -0.0335 | 0.0613 | 0.5844 |
| rs1468908 | G | A | 0.4920 | 0.0050 | 0.0004 | 6.28E-37 | 0.5386 | 0.0156 | 0.0161 | 0.3320 |
| rs1483842 | C | T | 0.4808 | -0.0028 | 0.0004 | 1.42E-12 | 0.4855 | 0.0034 | 0.0160 | 0.8294 |
| rs1570603 | A | G | 0.5720 | -0.0022 | 0.0004 | 3.69E-08 | 0.5824 | 0.0085 | 0.0161 | 0.5964 |
| rs17036160 | T | C | 0.1180 | -0.0040 | 0.0006 | 5.67E-11 | 0.1708 | -0.0665 | 0.0213 | 0.0018 |
| rs17288460 | A | G | 0.1803 | -0.0033 | 0.0005 | 7.84E-11 | 0.1763 | -0.0091 | 0.0209 | 0.6649 |
| rs17884869 | A | G | 0.0250 | 0.0079 | 0.0013 | 2.58E-10 | 0.0363 | 0.0176 | 0.0427 | 0.6806 |
| rs1889208 | A | G | 0.3359 | 0.0033 | 0.0004 | 3.76E-15 | 0.3859 | 0.0199 | 0.0164 | 0.2241 |
| rs209961 | C | T | 0.7219 | 0.0037 | 0.0004 | 1.38E-17 | 0.7851 | 0.0303 | 0.0194 | 0.1187 |
| rs2177470 | A | T | 0.6819 | -0.0027 | 0.0004 | 2.60E-10 | 0.7257 | 0.0102 | 0.0180 | 0.5699 |
| rs2420055 | C | T | 0.8426 | -0.0035 | 0.0005 | 4.21E-11 | 0.8669 | -0.0536 | 0.0234 | 0.0219 |
| rs273587 | T | A | 0.6778 | -0.0030 | 0.0004 | 7.53E-13 | 0.6251 | -0.0150 | 0.0165 | 0.3640 |
| rs28542318 | T | A | 0.8344 | 0.0032 | 0.0005 | 1.75E-09 | 0.7485 | 0.0272 | 0.0194 | 0.1599 |
| rs2909212 | G | A | 0.2966 | 0.0024 | 0.0004 | 2.31E-08 | 0.2797 | -0.0161 | 0.0178 | 0.3646 |
| rs2959652 | T | G | 0.9080 | -0.0037 | 0.0007 | 3.13E-08 | 0.8946 | 0.0607 | 0.0260 | 0.0196 |
| rs296847 | T | G | 0.6643 | 0.0024 | 0.0004 | 8.87E-09 | 0.6396 | -0.0057 | 0.0166 | 0.7290 |
| rs2970818 | A | T | 0.1038 | 0.0206 | 0.0006 | 1.00E-200 | 0.0624 | 0.0865 | 0.0329 | 0.0086 |
| rs2982572 | T | C | 0.4208 | -0.0041 | 0.0004 | 1.83E-25 | 0.4618 | -0.0407 | 0.0160 | 0.0107 |
| rs308032 | T | G | 0.2368 | -0.0029 | 0.0005 | 1.03E-09 | 0.1670 | -0.0258 | 0.0214 | 0.2288 |
| rs3091842 | A | G | 0.0441 | -0.0086 | 0.0010 | 1.49E-18 | 0.0885 | -0.0447 | 0.0283 | 0.1144 |
| rs339969 | A | C | 0.6148 | 0.0027 | 0.0004 | 2.35E-11 | 0.7196 | -0.0111 | 0.0177 | 0.5305 |
| rs35026779 | T | C | 0.0997 | -0.0042 | 0.0007 | 9.54E-11 | 0.0983 | -0.0059 | 0.0269 | 0.8275 |
| rs35666081 | A | G | 0.4002 | 0.0022 | 0.0004 | 4.52E-08 | 0.4771 | -0.0054 | 0.0160 | 0.7379 |
| rs3813498 | T | C | 0.8128 | 0.0051 | 0.0005 | 3.42E-24 | 0.7759 | 0.0453 | 0.0192 | 0.0179 |
| rs4072537 | C | T | 0.6811 | -0.0023 | 0.0004 | 3.95E-08 | 0.6385 | 0.0180 | 0.0168 | 0.2829 |
| rs4077450 | T | G | 0.8280 | -0.0042 | 0.0005 | 1.15E-15 | 0.7978 | -0.0318 | 0.0199 | 0.1099 |
| rs41278174 | A | G | 0.0273 | 0.0170 | 0.0012 | 1.31E-45 | 0.0330 | -0.0875 | 0.0445 | 0.0495 |
| rs4509682 | A | G | 0.3919 | -0.0031 | 0.0004 | 5.48E-15 | 0.3749 | -0.0345 | 0.0164 | 0.0357 |
| rs453639 | A | C | 0.6405 | -0.0107 | 0.0004 | 2.10E-150 | 0.6653 | -0.0321 | 0.0172 | 0.0624 |
| rs4706554 | A | G | 0.4763 | 0.0029 | 0.0004 | 2.22E-13 | 0.4134 | -0.0171 | 0.0161 | 0.2891 |
| rs4759844 | G | A | 0.3509 | 0.0038 | 0.0004 | 1.22E-20 | 0.2858 | 0.0225 | 0.0177 | 0.2019 |
| rs4795607 | T | C | 0.6140 | 0.0024 | 0.0004 | 1.58E-09 | 0.5962 | 0.0142 | 0.0164 | 0.3852 |
| rs4820324 | C | G | 0.5810 | 0.0030 | 0.0004 | 3.78E-14 | 0.5821 | 0.0174 | 0.0161 | 0.2802 |
| rs4869745 | T | C | 0.2871 | 0.0031 | 0.0004 | 6.73E-13 | 0.2945 | 0.0362 | 0.0176 | 0.0394 |
| rs4938642 | C | G | 0.0740 | 0.0041 | 0.0007 | 4.74E-08 | 0.0272 | -0.0002 | 0.0499 | 0.9969 |
| rs4982711 | G | T | 0.5710 | 0.0023 | 0.0004 | 1.05E-08 | 0.5683 | 0.0396 | 0.0161 | 0.0138 |
| rs5435 | C | T | 0.6121 | -0.0022 | 0.0004 | 4.08E-08 | 0.6206 | 0.0048 | 0.0165 | 0.7690 |
| rs55923451 | A | G | 0.1791 | -0.0034 | 0.0005 | 5.39E-11 | 0.1408 | -0.0301 | 0.0230 | 0.1901 |
| rs56078331 | T | C | 0.0544 | 0.0084 | 0.0009 | 2.07E-22 | 0.0410 | 0.0789 | 0.0404 | 0.0509 |
| rs56328339 | T | C | 0.1628 | 0.0052 | 0.0005 | 1.05E-22 | 0.2083 | 0.0179 | 0.0197 | 0.3634 |
| rs596940 | C | T | 0.0716 | 0.0052 | 0.0008 | 1.16E-11 | 0.0758 | 0.0082 | 0.0302 | 0.7870 |
| rs60447213 | T | G | 0.0699 | -0.0299 | 0.0008 | 1.00E-200 | 0.1084 | 0.0023 | 0.0261 | 0.9302 |
| rs6136492 | T | C | 0.2259 | -0.0027 | 0.0005 | 5.88E-09 | 0.1897 | -0.0193 | 0.0203 | 0.3423 |
| rs61870274 | A | C | 0.0781 | 0.0059 | 0.0007 | 7.92E-16 | 0.0813 | 0.0137 | 0.0291 | 0.6384 |
| rs61909254 | C | T | 0.8598 | 0.0101 | 0.0006 | 8.49E-72 | 0.8420 | 0.0789 | 0.0221 | 0.0004 |
| rs62289280 | C | T | 0.3053 | -0.0029 | 0.0004 | 1.09E-11 | 0.2725 | 0.0115 | 0.0179 | 0.5208 |
| rs62383568 | C | T | 0.3497 | -0.0024 | 0.0004 | 3.35E-09 | 0.3252 | -0.0396 | 0.0170 | 0.0202 |
| rs6751217 | T | C | 0.6316 | 0.0029 | 0.0004 | 6.03E-13 | 0.6198 | -0.0171 | 0.0164 | 0.2985 |
| rs6780593 | A | G | 0.1528 | -0.0045 | 0.0005 | 8.55E-17 | 0.1774 | -0.0124 | 0.0208 | 0.5511 |
| rs6841258 | T | C | 0.1663 | 0.0031 | 0.0005 | 5.29E-09 | 0.1301 | -0.0423 | 0.0239 | 0.0768 |
| rs6911602 | A | G | 0.1318 | 0.0082 | 0.0006 | 5.07E-46 | 0.1112 | 0.0007 | 0.0254 | 0.9775 |
| rs6983239 | T | G | 0.2186 | 0.0039 | 0.0005 | 3.52E-16 | 0.1869 | 0.0192 | 0.0204 | 0.3476 |
| rs7017252 | T | C | 0.3830 | 0.0022 | 0.0004 | 3.53E-08 | 0.2944 | -0.0065 | 0.0176 | 0.7126 |
| rs7033278 | T | C | 0.2930 | 0.0035 | 0.0004 | 5.96E-16 | 0.3164 | -0.0370 | 0.0172 | 0.0313 |
| rs706300 | G | A | 0.8575 | -0.0054 | 0.0006 | 2.96E-22 | 0.8816 | 0.0241 | 0.0248 | 0.3328 |
| rs71368113 | G | C | 0.0709 | -0.0045 | 0.0008 | 4.44E-08 | 0.0579 | 0.0461 | 0.0343 | 0.1791 |
| rs71413981 | A | G | 0.1637 | -0.0031 | 0.0005 | 6.93E-09 | 0.1378 | -0.0271 | 0.0233 | 0.2453 |
| rs72660359 | T | G | 0.0373 | 0.0148 | 0.0010 | 1.30E-45 | 0.0354 | 0.0297 | 0.0436 | 0.4964 |
| rs72716022 | T | C | 0.2021 | -0.0033 | 0.0005 | 1.09E-10 | 0.1885 | 0.0178 | 0.0205 | 0.3856 |
| rs72974112 | T | C | 0.2373 | -0.0029 | 0.0005 | 4.78E-10 | 0.1811 | 0.0428 | 0.0210 | 0.0416 |
| rs7324259 | T | G | 0.1259 | 0.0037 | 0.0006 | 4.73E-10 | 0.1636 | 0.0047 | 0.0220 | 0.8295 |
| rs7529925 | T | C | 0.7383 | 0.0031 | 0.0004 | 7.01E-12 | 0.7223 | 0.0214 | 0.0178 | 0.2278 |
| rs7555660 | T | C | 0.0853 | 0.0090 | 0.0007 | 4.91E-38 | 0.0730 | 0.0522 | 0.0311 | 0.0935 |
| rs7625643 | G | A | 0.4482 | 0.0026 | 0.0004 | 7.39E-11 | 0.4593 | 0.0380 | 0.0160 | 0.0177 |
| rs7672820 | C | G | 0.1863 | 0.0043 | 0.0005 | 6.26E-18 | 0.1876 | 0.0183 | 0.0206 | 0.3734 |
| rs76819459 | G | A | 0.3270 | -0.0051 | 0.0004 | 1.15E-34 | 0.3097 | -0.0248 | 0.0173 | 0.1520 |
| rs77149268 | T | C | 0.1051 | 0.0066 | 0.0006 | 5.82E-25 | 0.0668 | -0.0105 | 0.0320 | 0.7440 |
| rs7726961 | T | C | 0.4261 | -0.0023 | 0.0004 | 6.85E-09 | 0.4069 | -0.0283 | 0.0163 | 0.0820 |
| rs77282753 | C | T | 0.0480 | -0.0069 | 0.0009 | 4.06E-14 | 0.0530 | 0.0835 | 0.0355 | 0.0187 |
| rs7860558 | C | T | 0.7013 | -0.0029 | 0.0004 | 3.13E-11 | 0.7083 | -0.0088 | 0.0177 | 0.6177 |
| rs79472736 | A | G | 0.0354 | -0.0070 | 0.0011 | 3.45E-11 | 0.0334 | 0.0309 | 0.0443 | 0.4849 |
| rs805288 | T | C | 0.2491 | 0.0025 | 0.0005 | 4.98E-08 | 0.3859 | 0.0134 | 0.0182 | 0.4618 |
| rs8075449 | T | C | 0.2590 | -0.0028 | 0.0004 | 5.03E-10 | 0.3386 | -0.0202 | 0.0169 | 0.2331 |
| rs838717 | A | G | 0.5656 | 0.0049 | 0.0004 | 3.04E-35 | 0.5845 | -0.0112 | 0.0162 | 0.4881 |
| rs872386 | A | G | 0.5310 | -0.0039 | 0.0004 | 2.60E-23 | 0.5873 | 0.0140 | 0.0162 | 0.3887 |
| rs896788 | T | C | 0.1552 | 0.0042 | 0.0005 | 6.52E-15 | 0.2137 | -0.0195 | 0.0195 | 0.3175 |
| rs912981 | C | A | 0.5810 | -0.0026 | 0.0004 | 3.22E-11 | 0.6534 | -0.0107 | 0.0169 | 0.5283 |
| rs9574586 | C | T | 0.3311 | -0.0023 | 0.0004 | 2.35E-08 | 0.3282 | 0.0137 | 0.0169 | 0.4182 |
| rs9689096 | C | A | 0.0639 | -0.0070 | 0.0008 | 4.31E-18 | 0.0319 | 0.0903 | 0.0460 | 0.0496 |
| rs9745989 | C | T | 0.6281 | 0.0025 | 0.0004 | 9.31E-10 | 0.6884 | 0.0338 | 0.0174 | 0.0522 |

The F-statistics for phosphate was 34.23. **Abbreviations:** **MR,** Mendelian randomization; **SNP**, single-nucleotide polymorphism; **EA**, effect allele; **OA**, other allele; **EAF**, effect allele frequency; **SE**, standard error.

**Table S4: Evaluation of heterogeneity and horizontal pleiotropy using different methods.**

| Outcome | nSNP | Cochran’s Q  statistic of IVW | Cochran’s Q  P of IVW | Cochran’s Q  statistic of MR Egger | Cochran’s Q  P of MR Egger | MR-Egger intercept P |
| --- | --- | --- | --- | --- | --- | --- |
|  |  |  |  |  |  |  |
| Coronary heart disease | 107 | 124.71 | 1.04E-01 | 124.53 | 9.38E-02 | 0.70 |
| Heart failure | 95 | 82.03 | 8.06E-01 | 82.02 | 7.85E-01 | 0.95 |
| Atrial fibrillation | 112 | 242.94 | 1.65E-11 | 242.94 | 1.11E-11 | 0.99 |
| Essential hypertension | 107 | 196.99 | 1.96E-07 | 196.49 | 1.60E-07 | 0.60 |
| VHD including RF | 107 | 142.25 | 1.08E-02 | 142.22 | 9.13E-03 | 0.88 |
| Non-rheumatic valve diseases | 107 | 190.82 | 8.47E-07 | 190.68 | 6.40E-07 | 0.79 |

**Abbreviations:** **SNPs**, single-nucleotide polymorphisms; **MR-Egger**, Mendelian randomization-Egger. **VHD**, valvular heart disease; **RF**, rheumatic fever; **IVW**, inverse variance weighted.

**Table S5: Associations of genetically predicted phosphate with cardiovascular diseases in the MR-PRESSO analysis.**

| **Outcome** | **nSNPs** | **Outliers** | **p-dis** | **p-glo** | **Outlier-****corrected** | |
| --- | --- | --- | --- | --- | --- | --- |
|  |  |  |  |  | **OR (95% CI)** | **pval** |
| Coronary heart disease | 107 | NA | - | - | - | - |
| Heart failure | 95 | NA | - | - | - | - |
| Atrial fibrillation | 108 | 6 | 0.999 | <0.001 | 0.81 (0.55-1.16) | 0.2469 |
| Essential hypertension | 106 | 1 | 0.835 | <0.001 | 1.43(0.79-2.58) | 0.2357 |
| VHD including RF | 107 | NA | - | - | - | - |
| Non-rheumatic valve diseases | 106 | 1 | 0.831 | <0.001 | 5.29(2.11-13.29) | 0.0006 |

Outliers for Atrial fibrillation: rs17036160, rs2982572, rs4509682, rs7625643, rs872386, rs9689096; the outlier for Essential hypertension: rs9745989; the outlier for non-rheumatic valve diseases: rs138278885. **Abbreviations: MR-PRESSO**, MR-pleiotropy residual sum and outlier; **nSNPs**, number of singe nucleotide polymorphisms; **p-glo**, p value for global test; **p-dis**, p value for distortion test; **OR**, odds ratio; **CI**, confidence interval; **NA**, not available; **VHD**, valvular heart disease; **RF**, rheumatic fever.
