# Supplementary material for: Genetically predicted phosphate and cardiovascular disease: A Mendelian randomization study
Source: Front Cardiovasc Med. 2022 Oct 5;9:973338. doi: 10.3389/fcvm.2022.973338 (PMC9579538; doi:10.3389/fcvm.2022.973338)

**Figure S1**: Scatter plot to visualize causal effect of phosphate on common cardiovascular diseases. (A) Phosphate on coronary heart disease. (B) Phosphate on heart failure. (C) Phosphate on atrial fibrillation. (D) Phosphate on essential hypertension. (E) Phosphate on valvular heart disease including rheumatic fever. (F) Phosphate on non-rheumatic valve heart diseases. The slope of the straight line indicates the magnitude of the causal association. **Abbreviations:** **MR**, Mendelian randomization; **MR-PRESSO**, MR pleiotropy residual sum and outlier; **MR-RAPS**, MR using robust adjusted profile score.

**A**


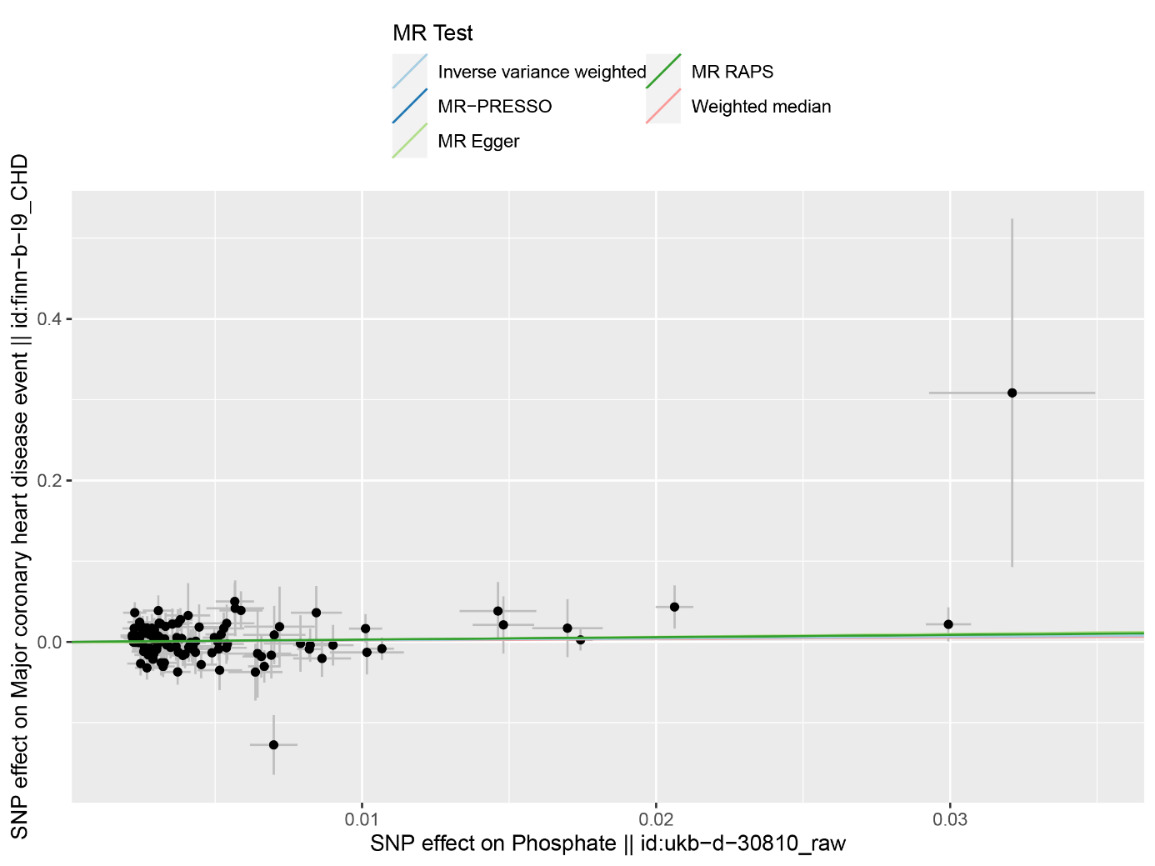


**B**


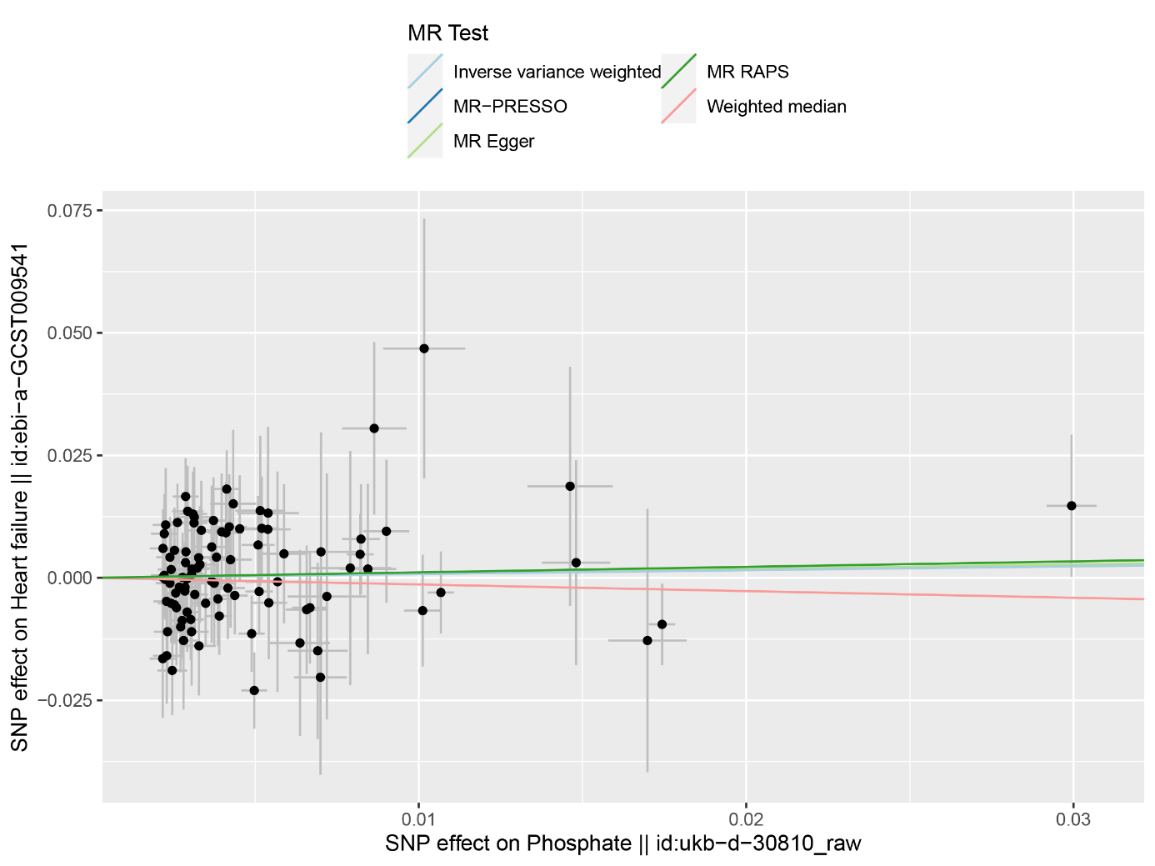


**C**


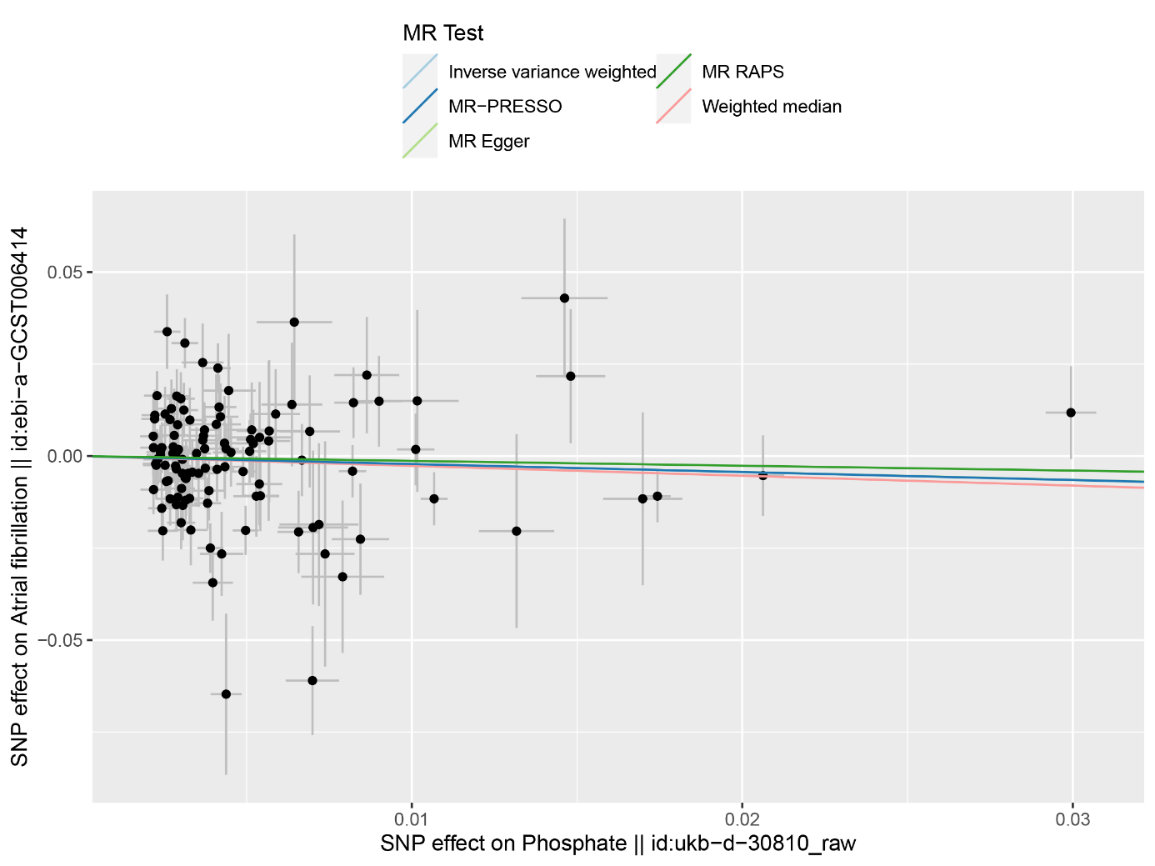


**D**


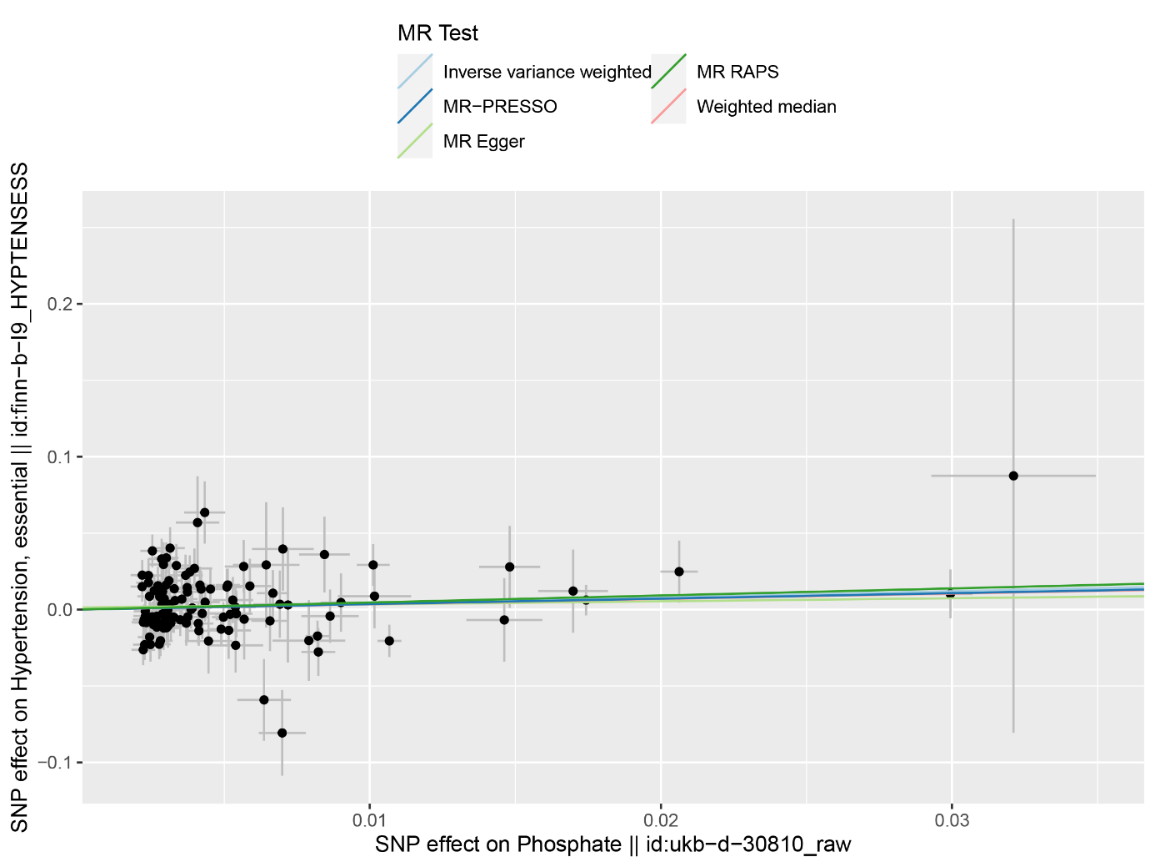


**E**


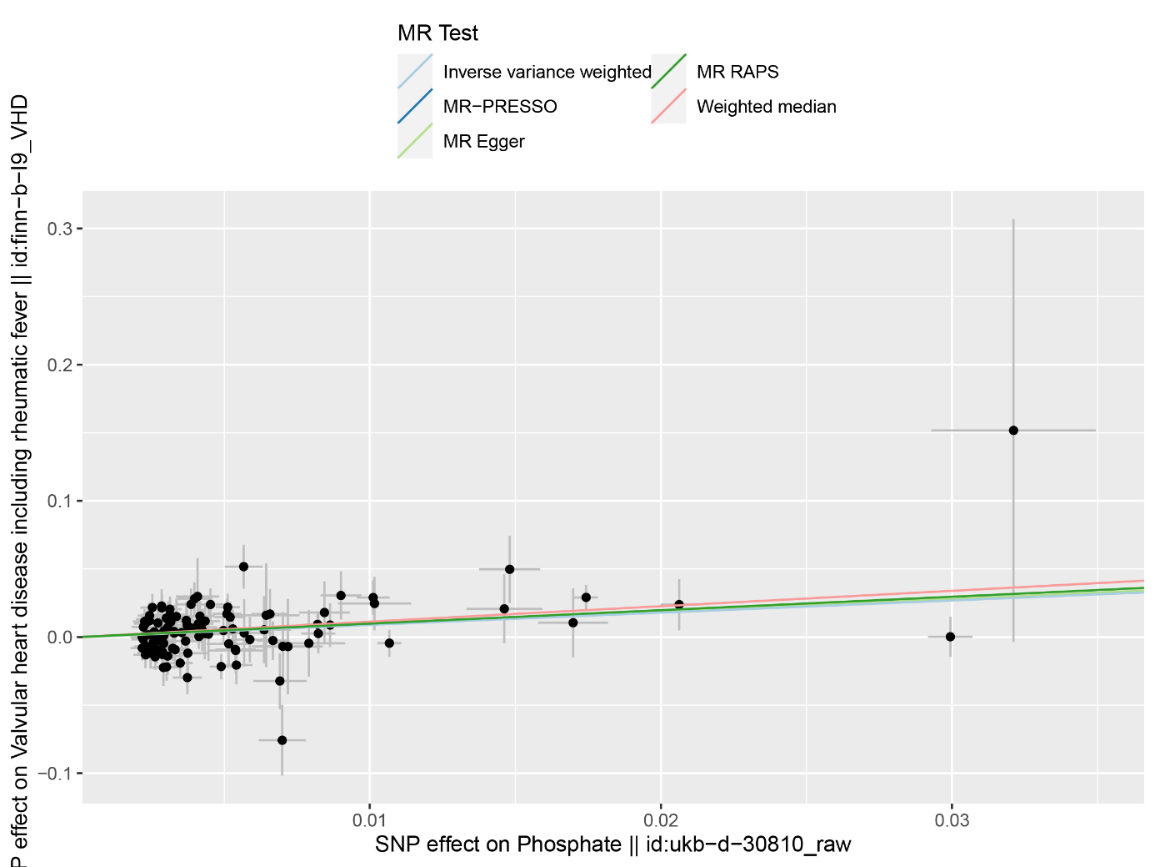


**F**


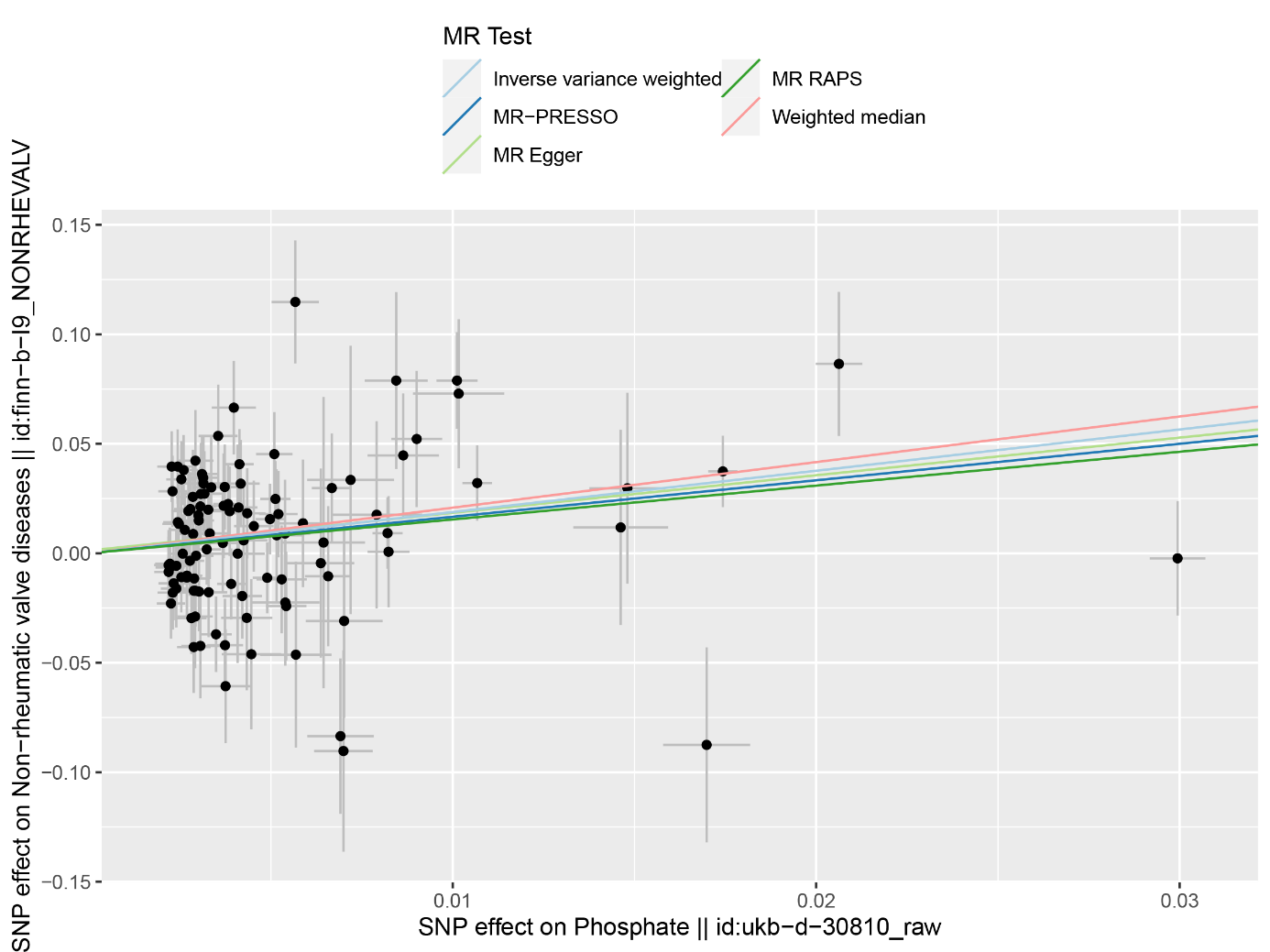


**Figure S2**: Forest plot to visualize the relationship between each genetic variant for phosphate and cardiovascular diseases. (A) Phosphate and coronary heart disease. (B) Phosphate and heart failure. (C) Phosphate and atrial fibrillation. (D) Phosphate and essential hypertension. (E) Phosphate and valvular heart disease including rheumatic fever. (F) Phosphate and non-rheumatic valve heart diseases. **Abbreviations: MR**, Mendelian randomization.

**A**


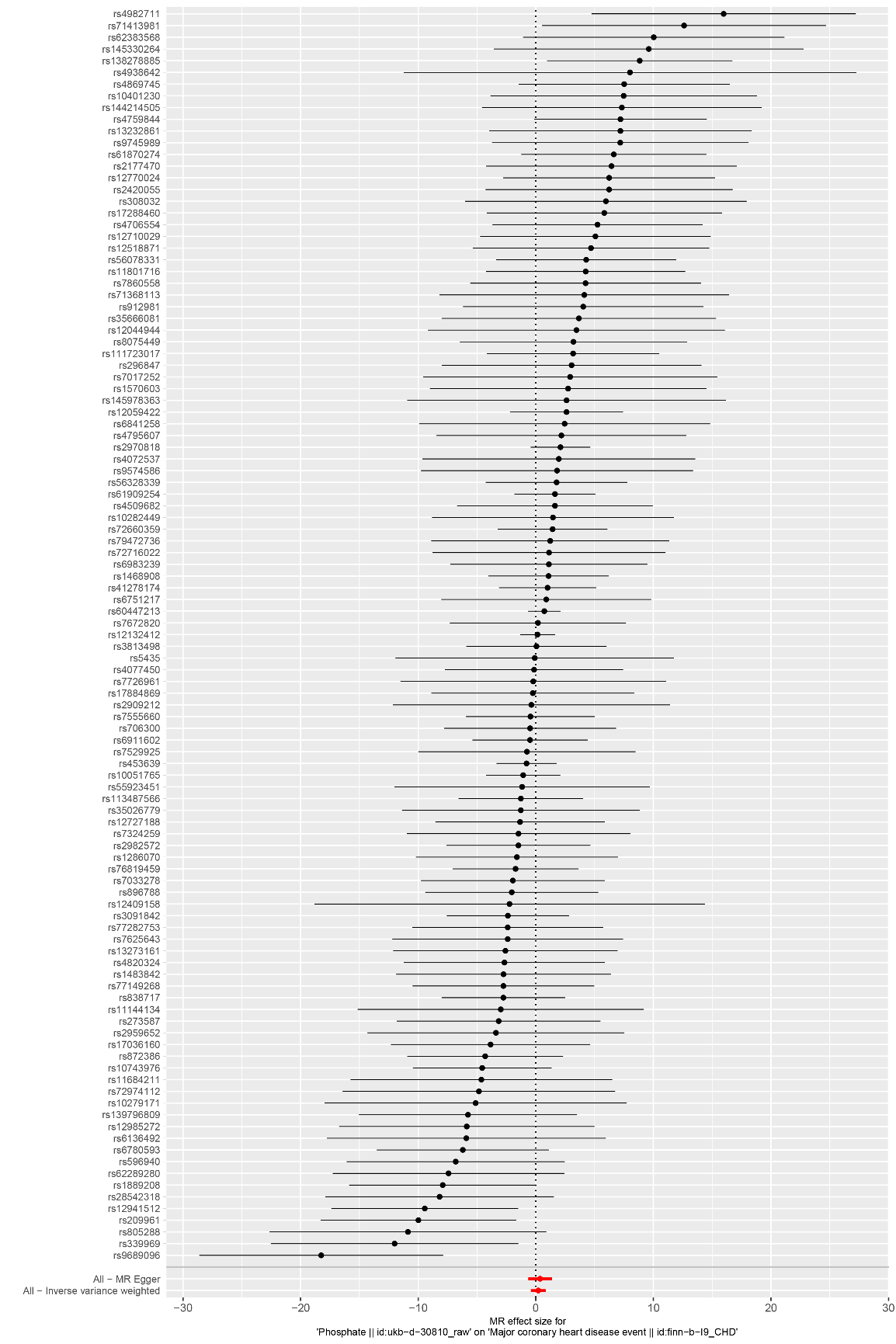


**B**


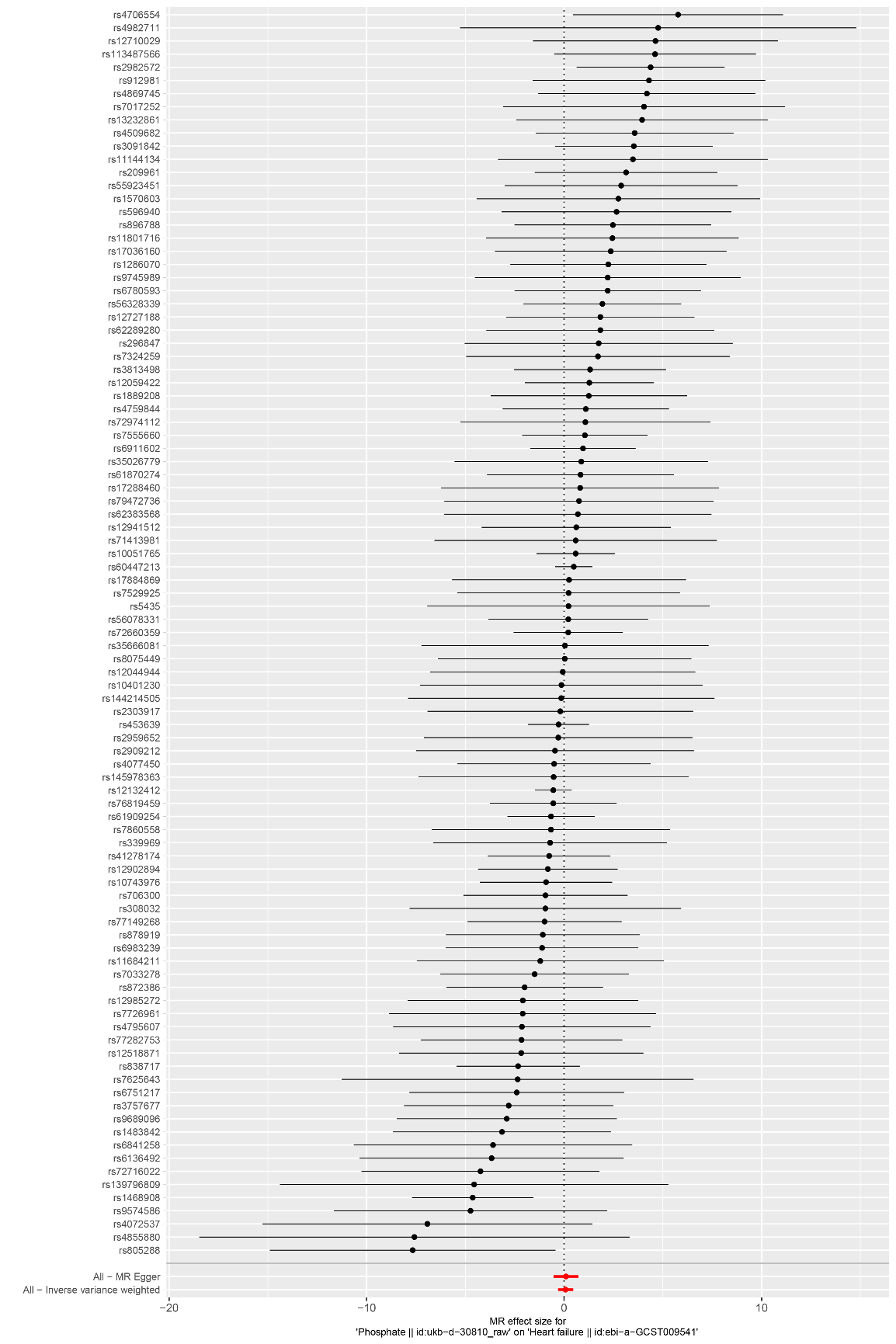


**C**


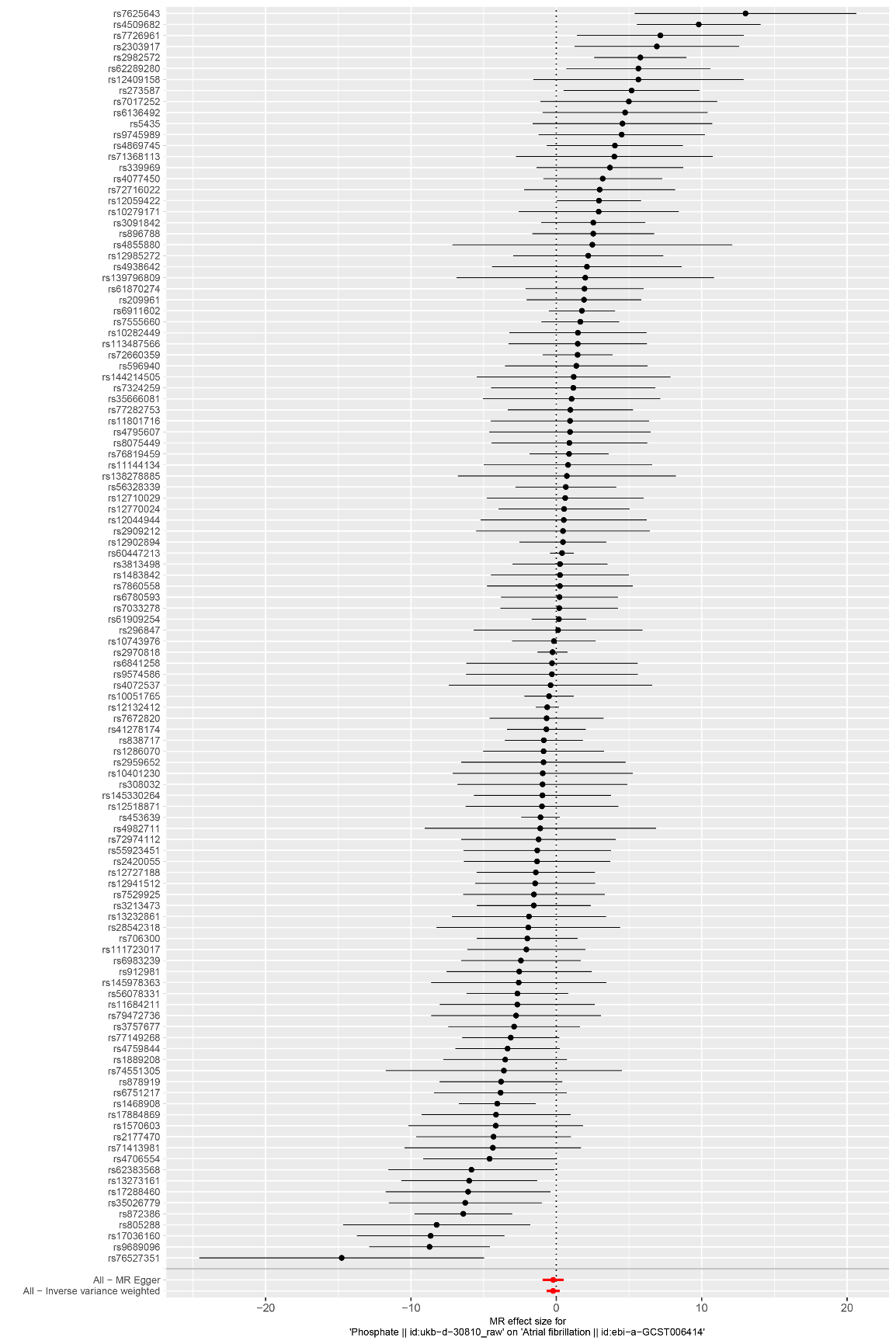


**D**


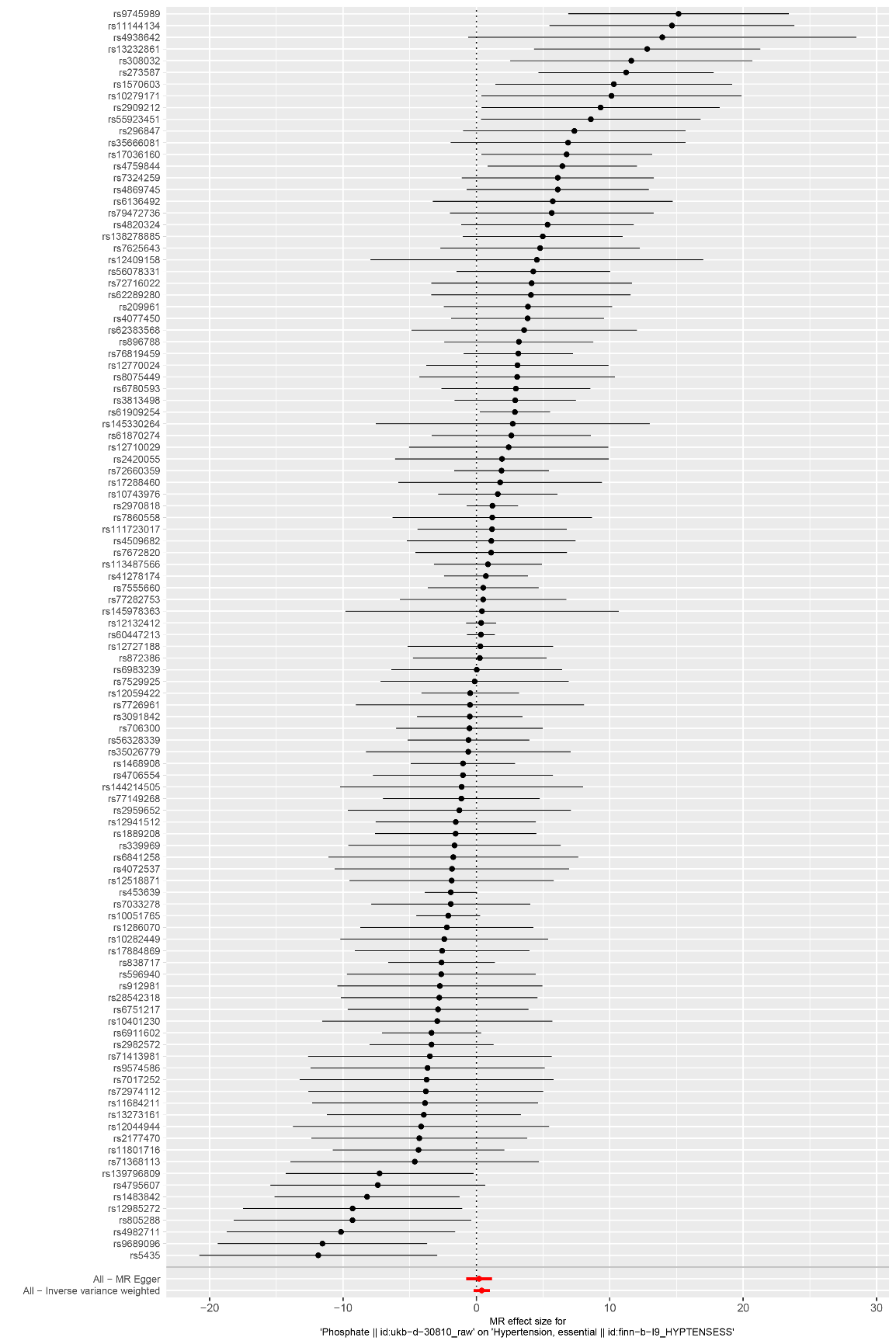


**E**


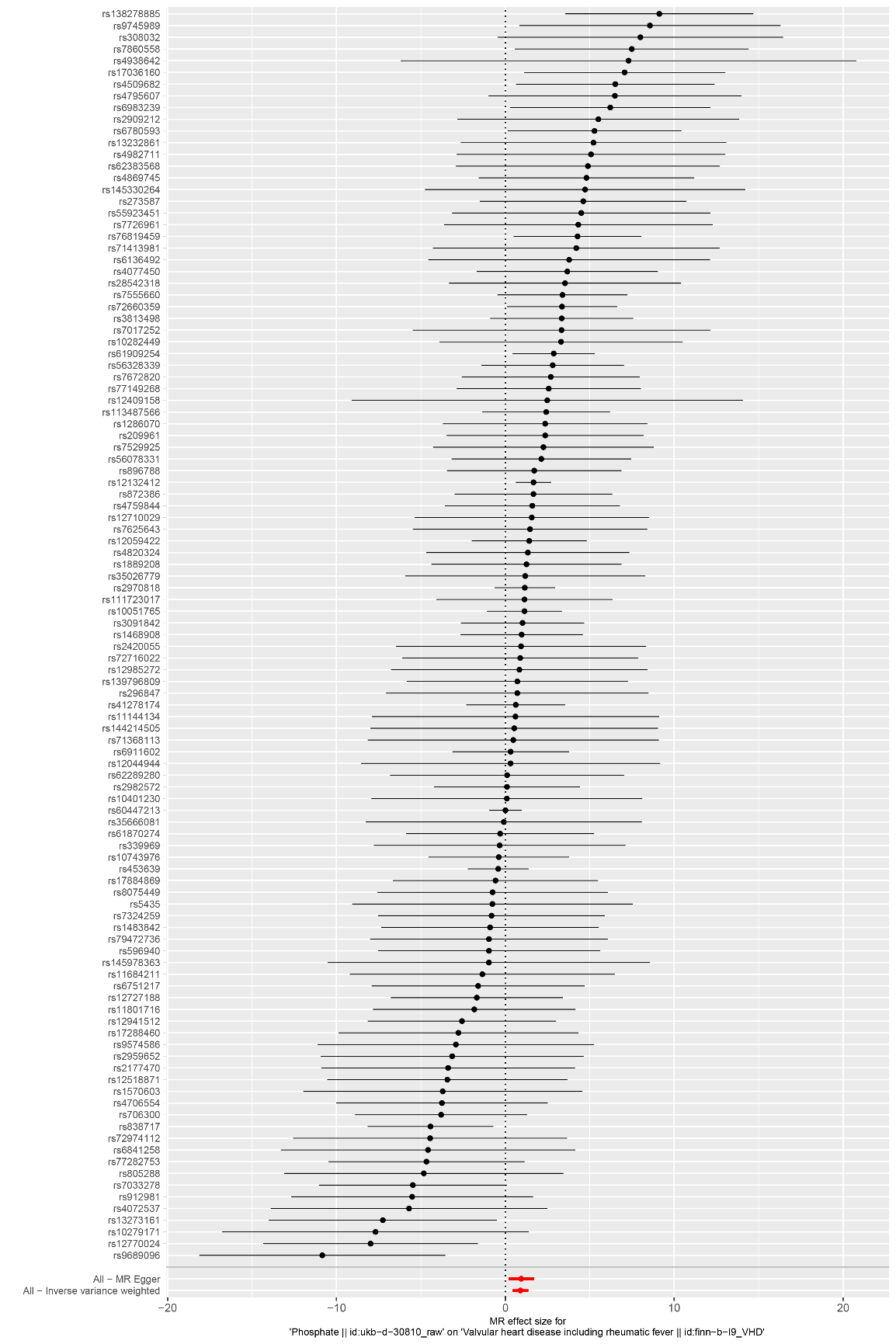


**F**


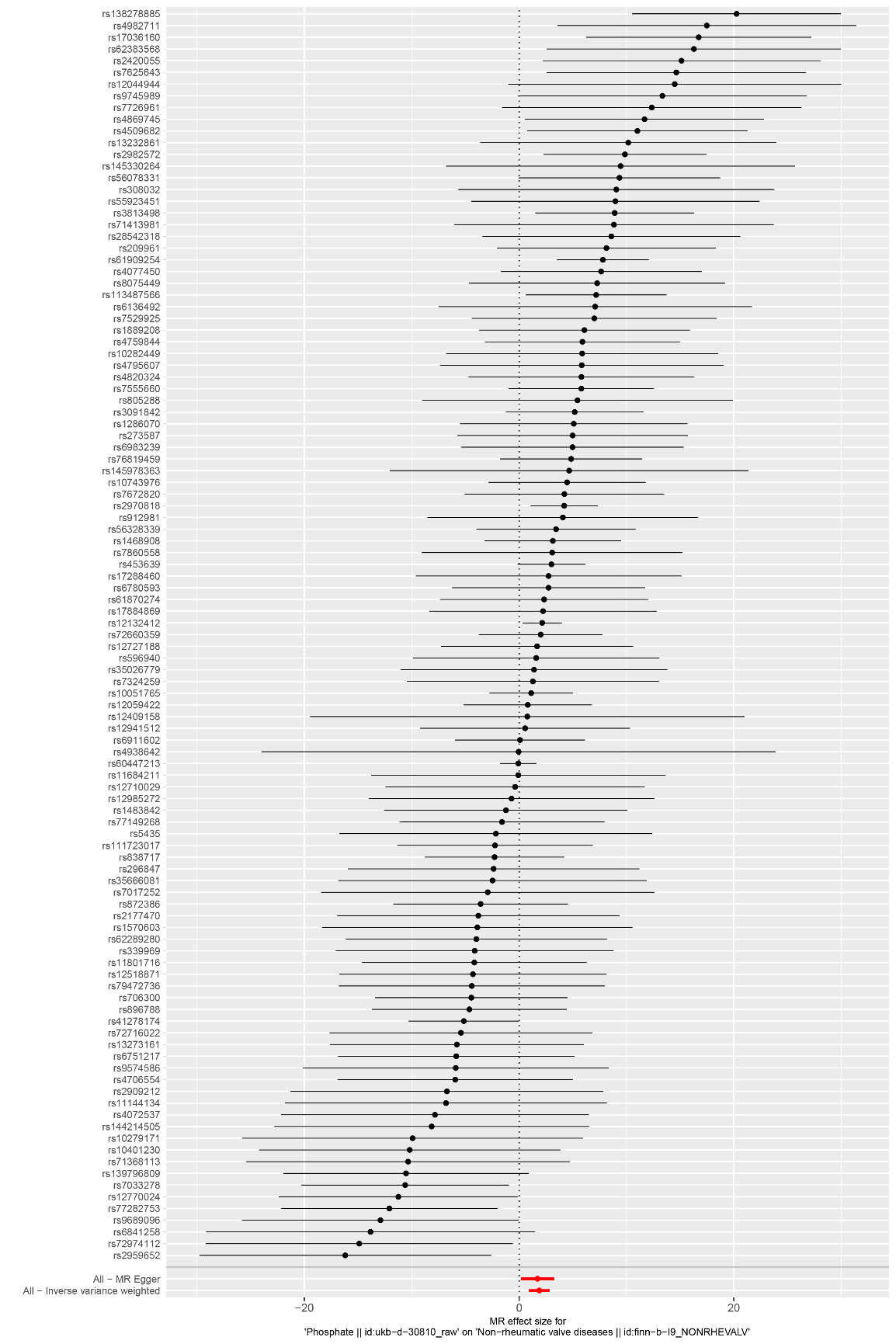


**Figure S3:** Funnel plots to visualize heterogeneity of MR estimates for the effect of phosphate on cardiovascular diseases. (A) Phosphate on coronary heart disease. (B) Phosphate on heart failure. (C) Phosphate on atrial fibrillation. (D) Phosphate on essential hypertension. (E) Phosphate on valvular heart disease including rheumatic fever. (F) Phosphate on non-rheumatic valve heart diseases. **Abbreviations:** **MR**, Mendelian randomization.

**A**


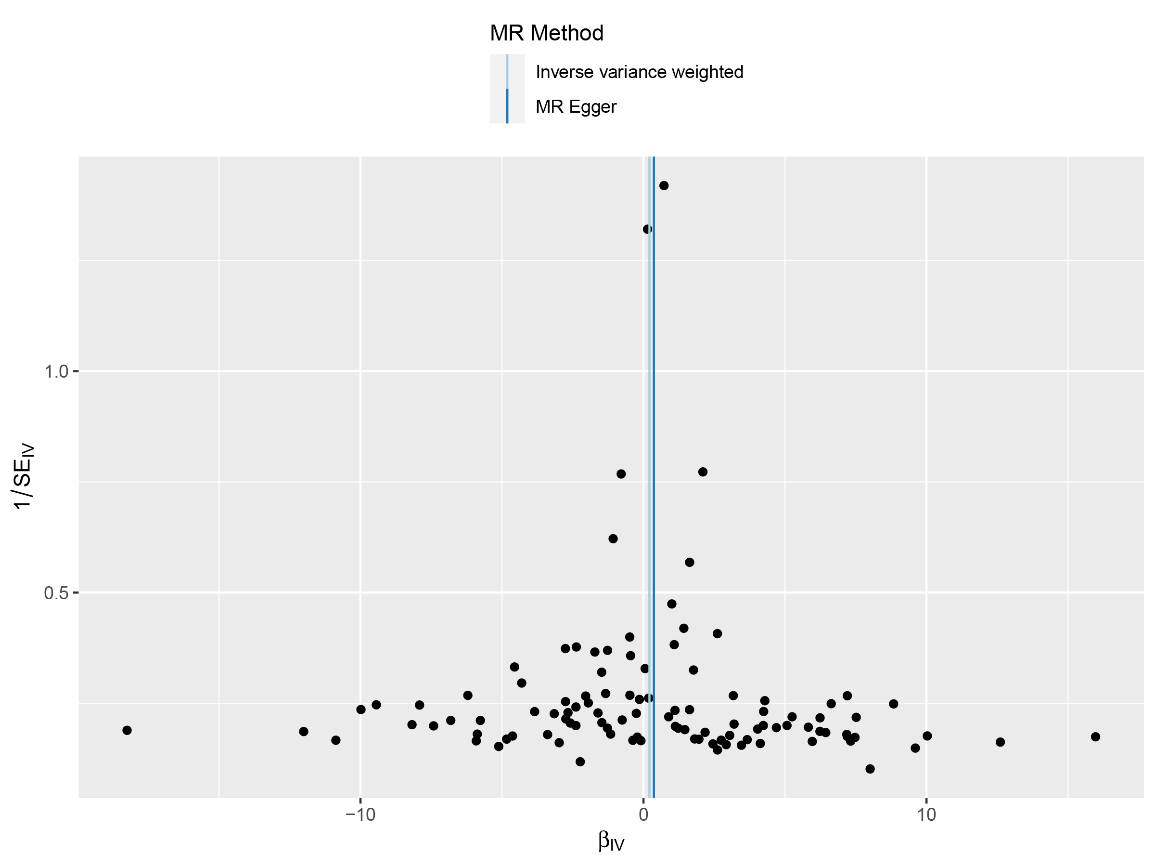


**B**


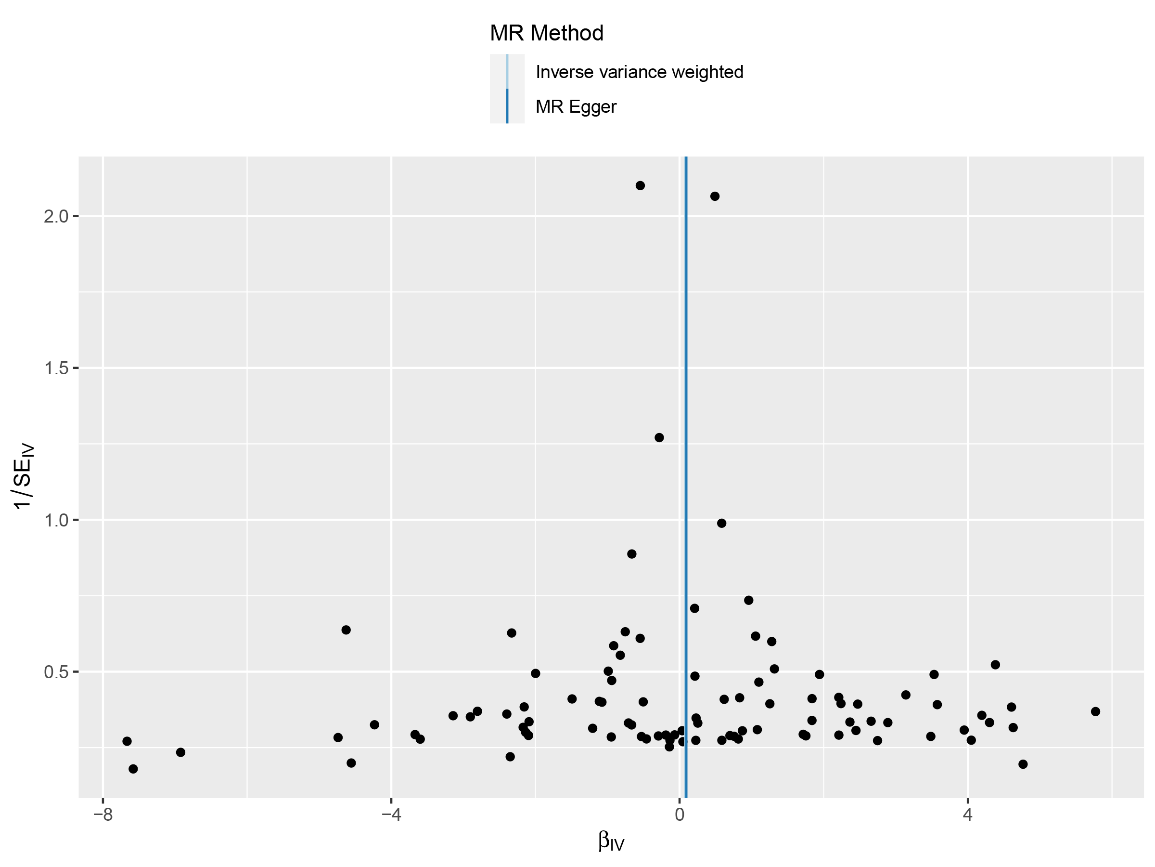


**C**


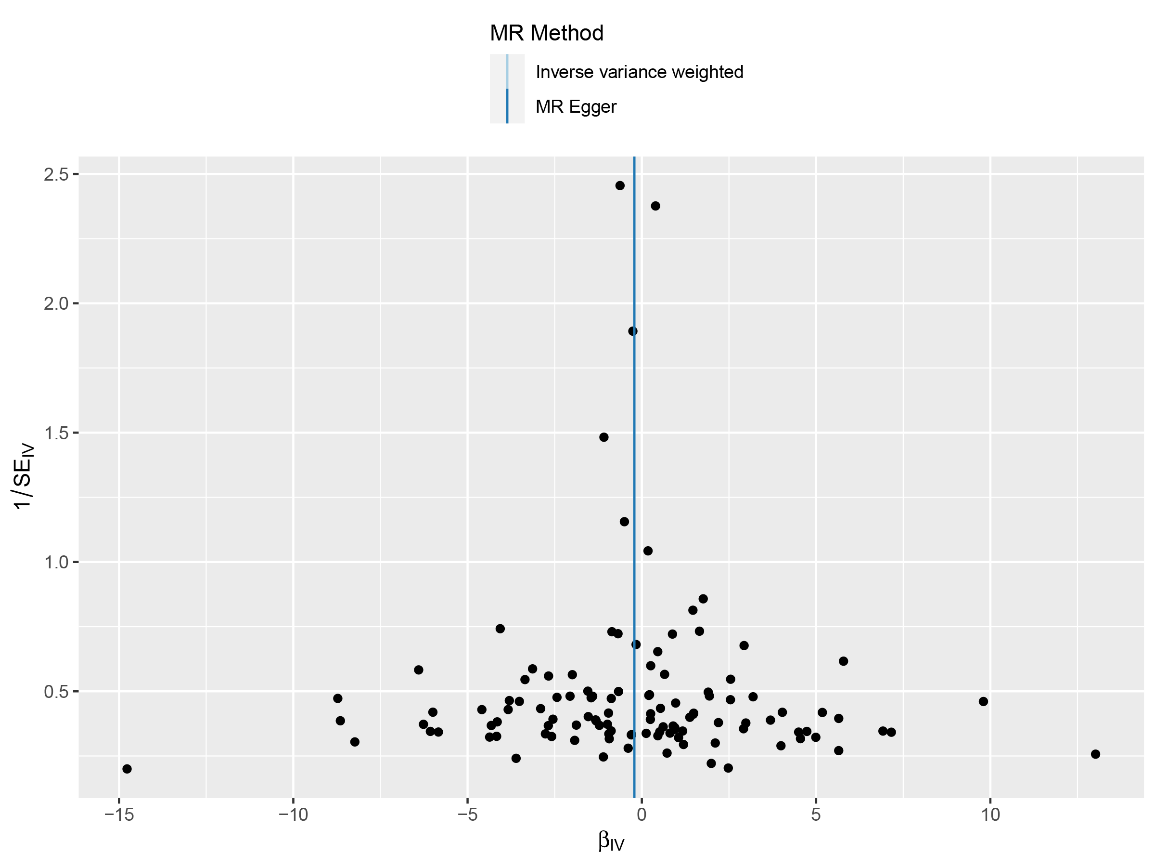


**D**


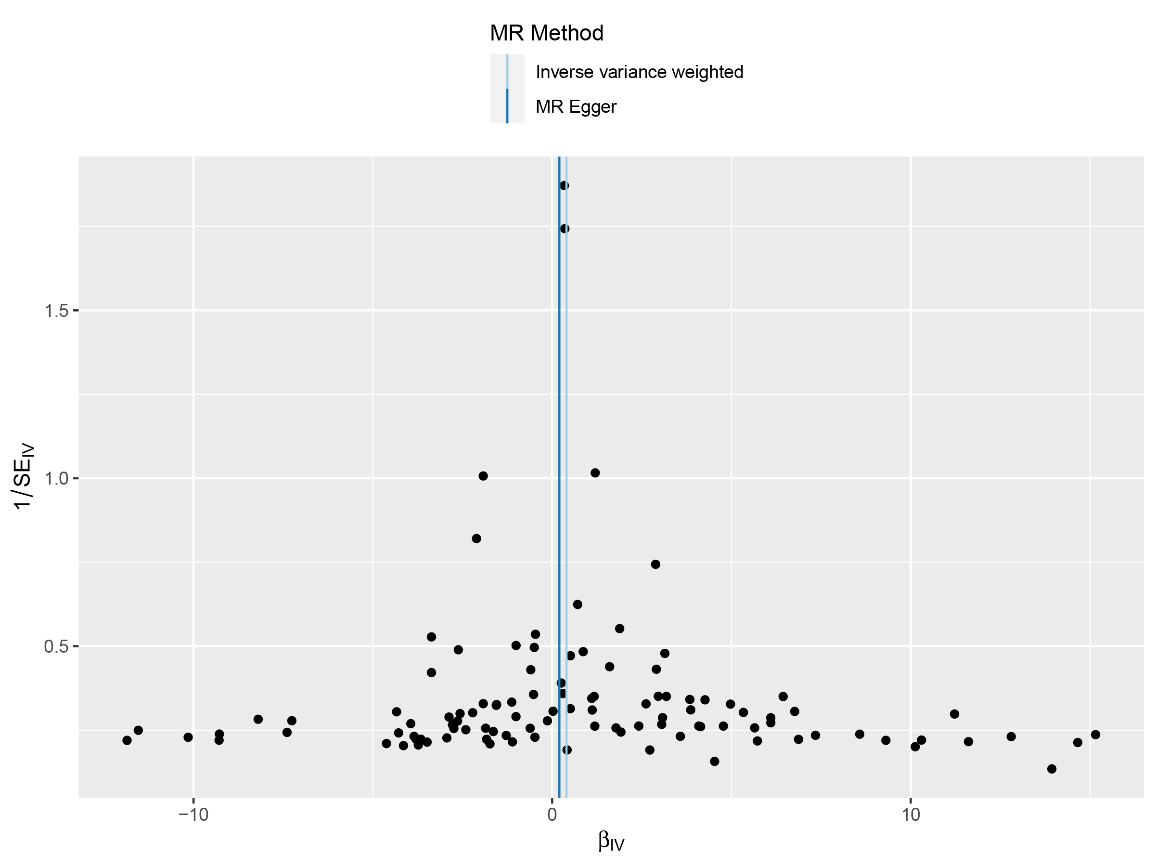


**E**


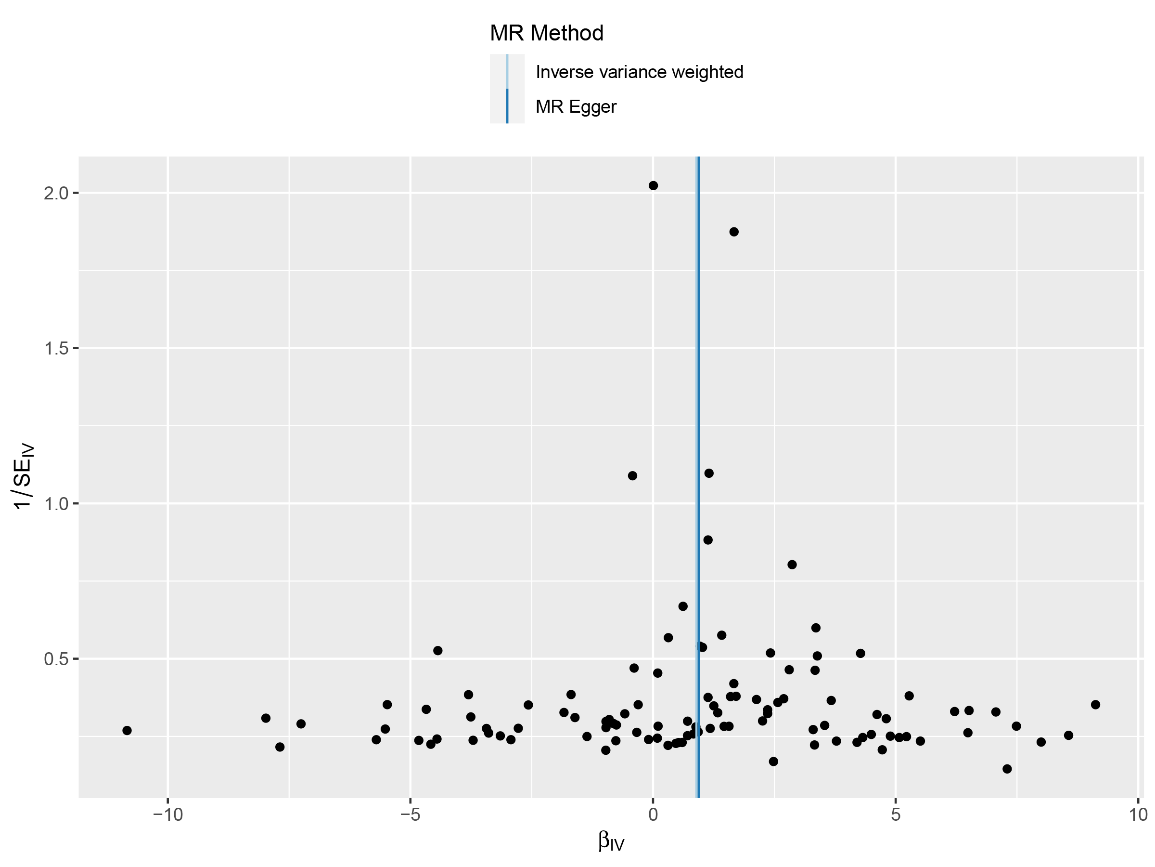


**F**


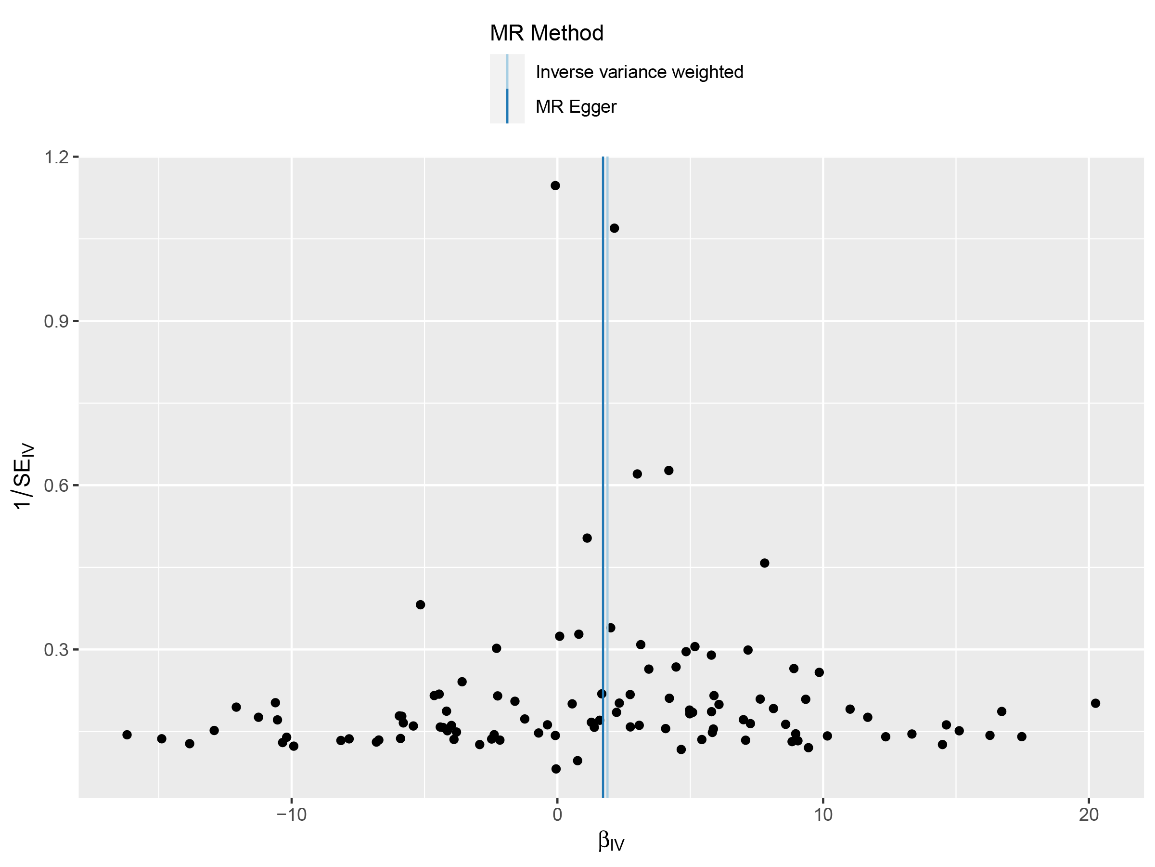


**Figure S4:** Leave-one-out analysis of the sensitivity of each genetic variant for phosphate and cardiovascular diseases. (A) Coronary heart disease. (B)Heart failure. (C) Atrial fibrillation. (D) Essential hypertension. (E) Valvular heart disease including rheumatic fever. (F) Non-rheumatic valve heart diseases. **Abbreviations:** **MR**, Mendelian randomization.

**A**


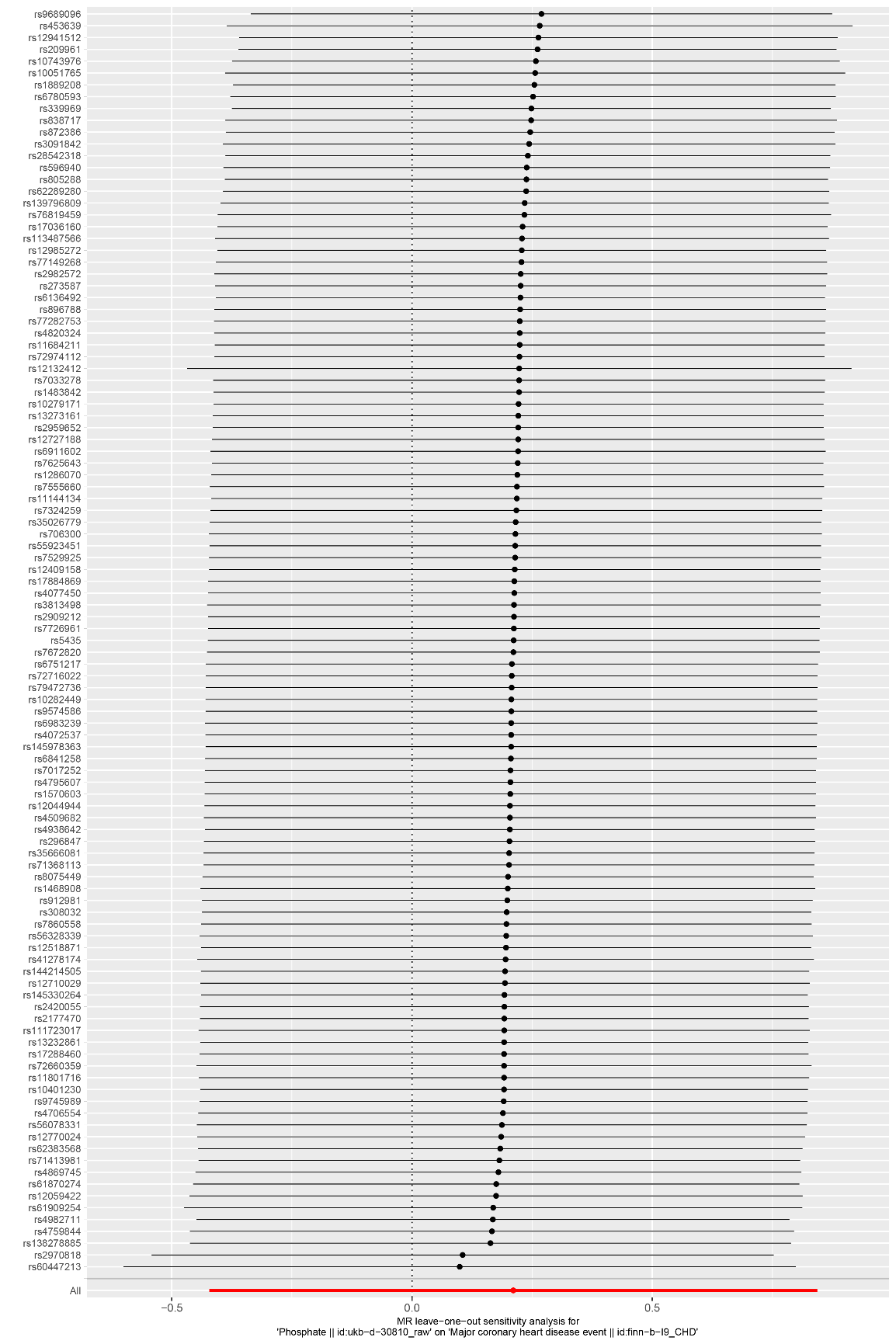


**B**


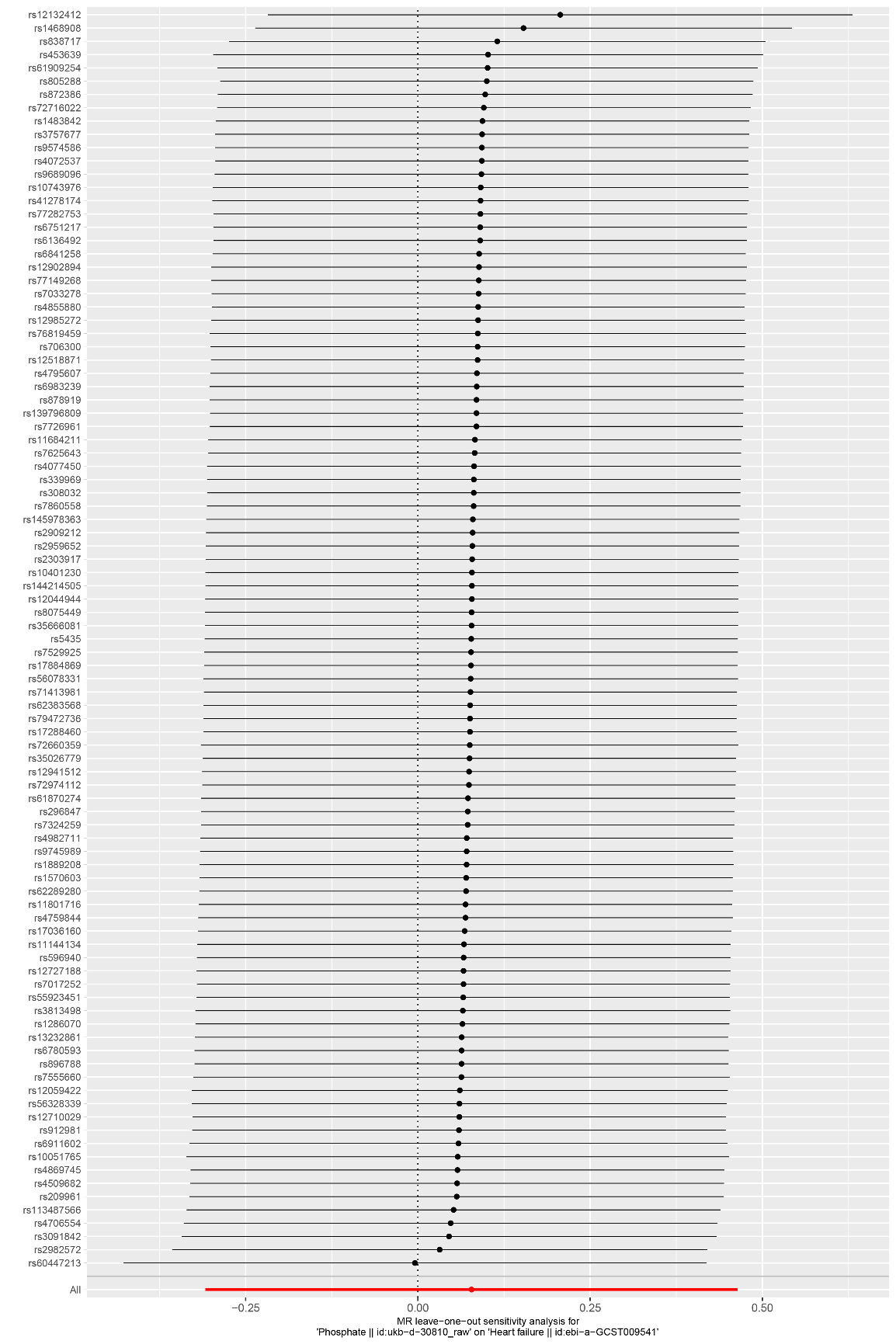


**C**


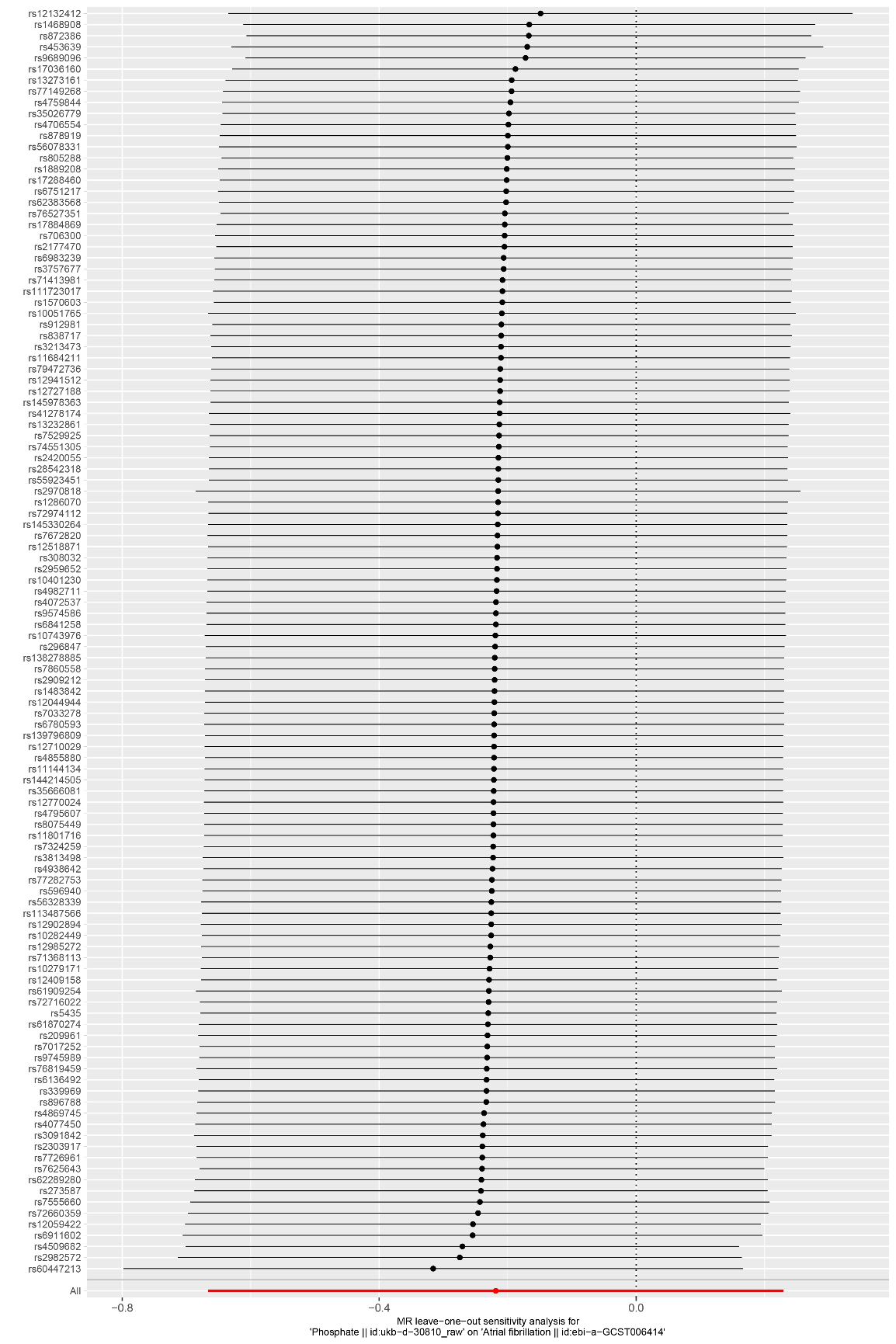


**D**


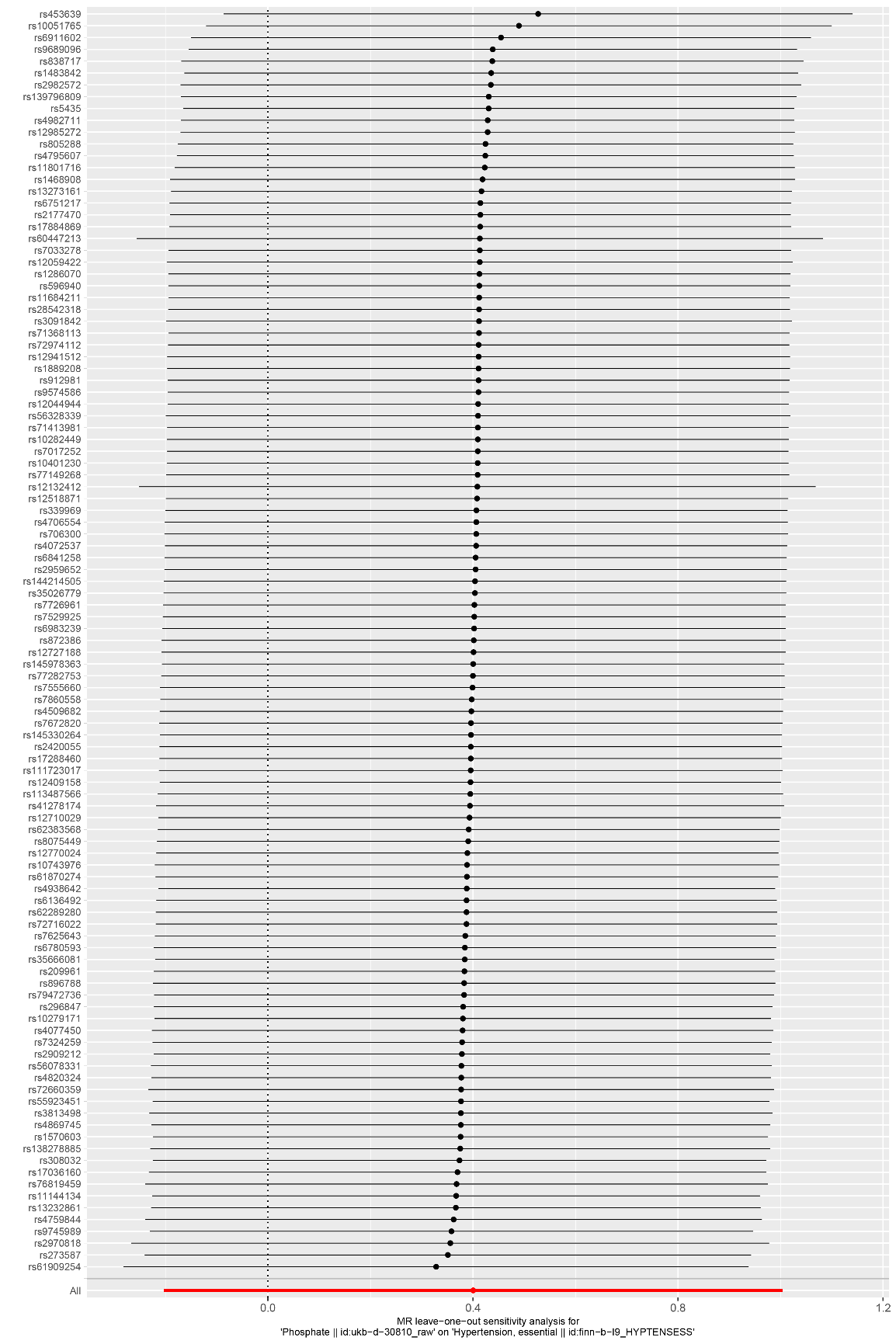


**E**


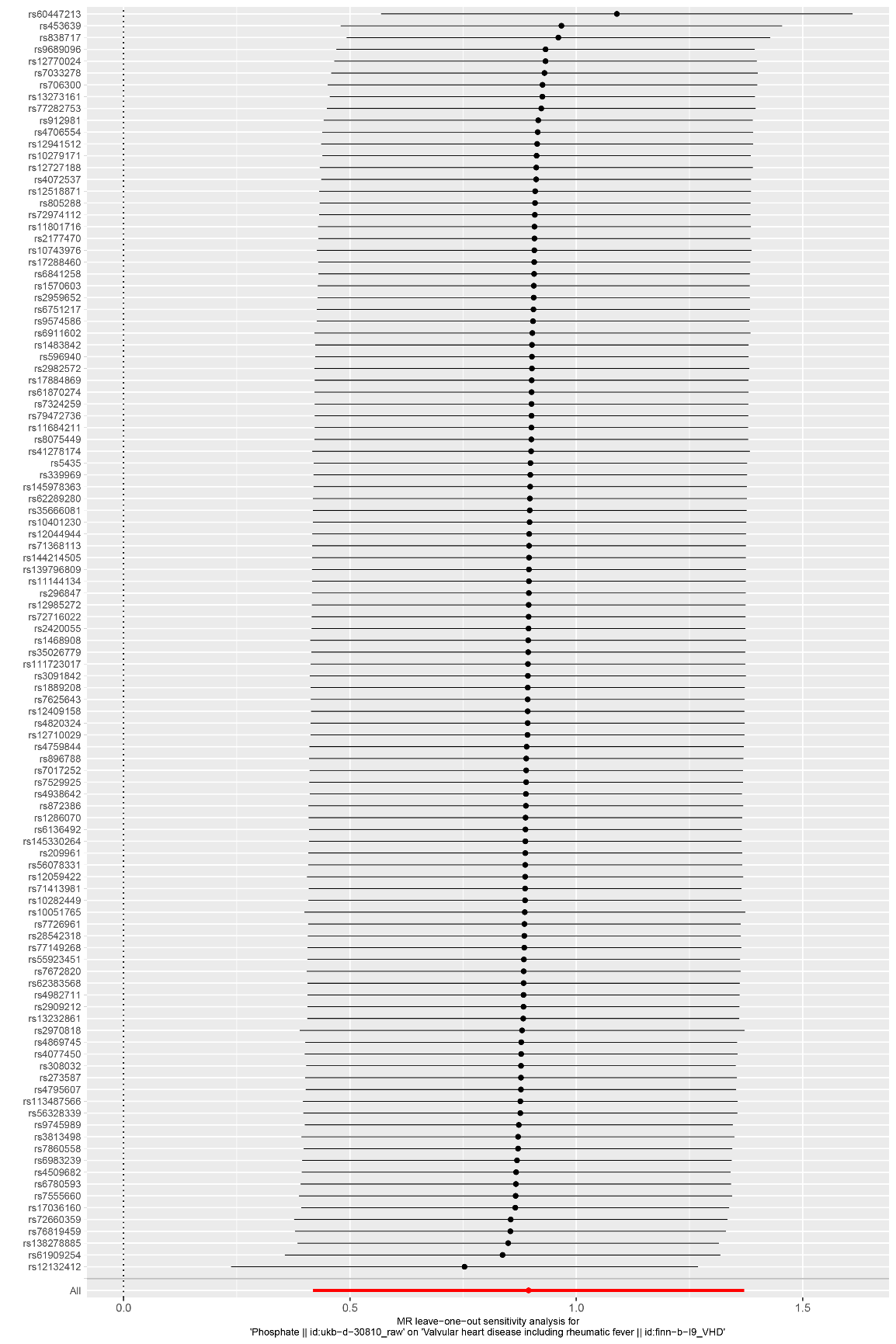


**F**


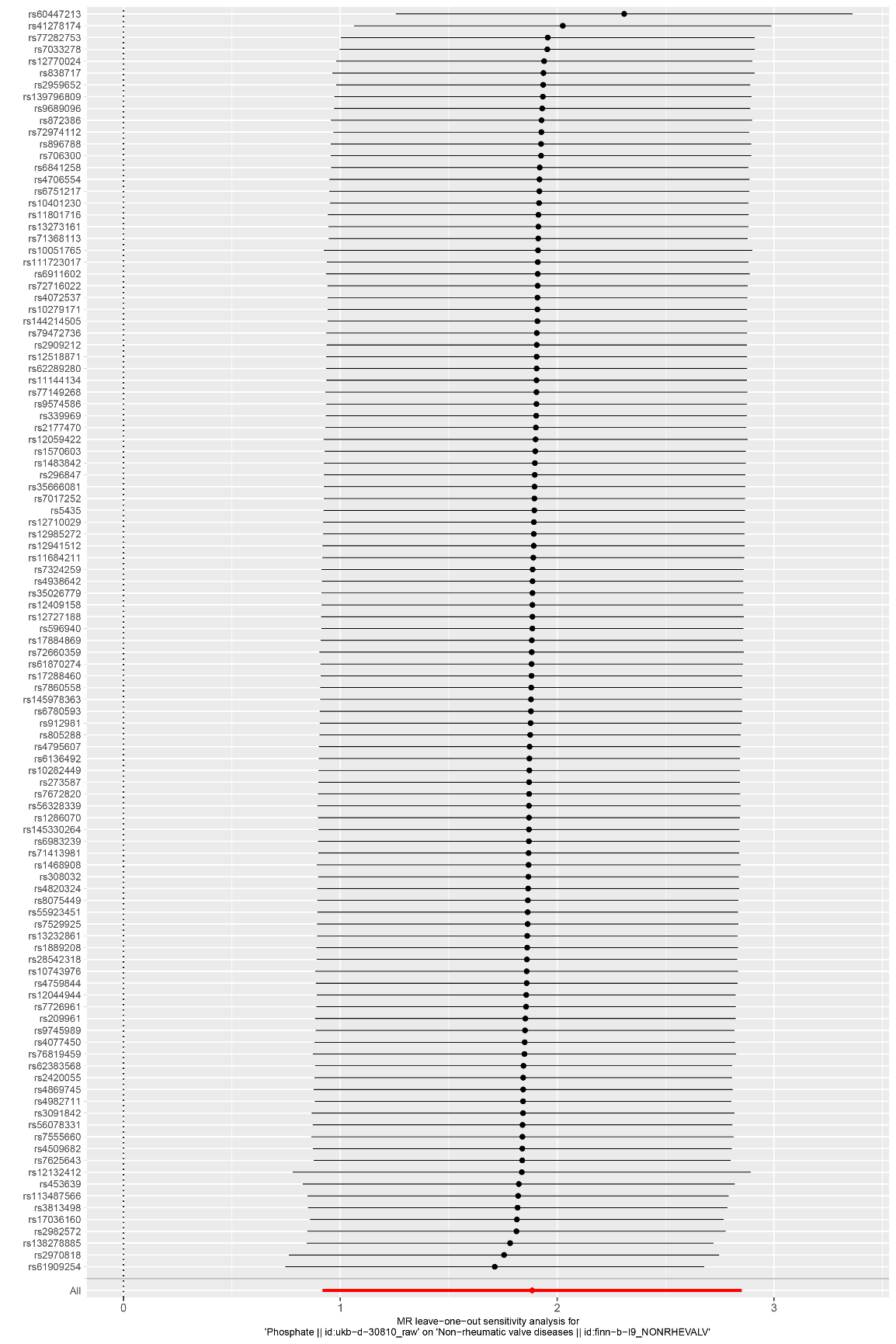

Supplement: Supplementary file 2 [file Data_Sheet_2.docx]
